# Optimization of the MACE endpoint composition to increase power in studies of lipid-lowering therapies—a model-based meta-analysis

**DOI:** 10.3389/fcvm.2023.1242845

**Published:** 2024-01-08

**Authors:** Alina Volkova, Boris Shulgin, Gabriel Helmlinger, Kirill Peskov, Victor Sokolov

**Affiliations:** ^1^ Modeling and Simulation Decisions FZ—LLC, Dubai, United Arab Emirates; ^2^ Sirius University of Science and Technology, Sirius, Russia; ^3^ Research Center of Model-Informed Drug Development, Sechenov First Moscow State Medical University, Moscow, Russia; ^4^ Biorchestra Co., Ltd., Cambridge, MA, United States

**Keywords:** MACE, cardiovascular disease, dyslipidemia biomarkers, lipid-lowering medication, model-based meta-analysis, sample size

## Abstract

**Aims:**

To develop a model-informed methodology for the optimization of the Major Adverse Cardiac Events (MACE) composite endpoint, based on a model-based meta-analysis across anti-hypercholesterolemia trials of statin and anti-PCSK9 drugs.

**Methods and results:**

Mixed-effects meta-regression modeling of stand-alone MACE outcomes was performed, with therapy type, population demographics, baseline and change over time in lipid biomarkers as predictors. Randomized clinical trials up to June 28, 2022, of either statins or anti-PCSK9 therapies were identified through a systematic review process in PubMed and ClinicalTrials.gov databases. In total, 54 studies (270,471 patients) were collected, reporting 15 different single cardiovascular events. Treatment-mediated decrease in low density lipoprotein cholesterol, baseline levels of remnant and high-density lipoprotein cholesterol as well as non-lipid population characteristics and type of therapy were identified as significant covariates for 10 of the 15 outcomes. The required sample size per composite 3- and 4-point MACE endpoint was calculated based on the estimated treatment effects in a population and frequencies of the incorporated events in the control group, trial duration, and uncertainty in model parameters.

**Conclusion:**

A quantitative tool was developed and used to benchmark different compositions of 3- and 4-point MACE for statins and anti-PCSK9 therapies, based on the minimum population size required to achieve statistical significance in relative risk reduction, following meta-regression modeling of the single MACE components. The approach we developed may be applied towards the optimization of the design of future trials in dyslipidemia disorders as well as in other therapeutic areas.

## 1 Introduction

Cardiovascular (CV) diseases, including coronary heart disease (CHD) and stroke—the leading causes of mortality in developed countries—are strongly associated with the development of atherosclerosis (1). Extensive preclinical and clinical data have revealed biomarkers for dyslipidemia to be causal in the onset and progression of cardiovascular diseases, with low-density lipoprotein cholesterol (LDLc) being a primary target in multiple treatment options (2, 3). Statins, or 3-hydroxy-3-methylglutaryl-coenzyme inhibitors, are the gold standard therapy for the management of plasma LDLc levels and dramatically reduce the risk of life-threatening cardiovascular events (3, 4). However, the use of statins is associated with unwanted side effects, including muscle pain and headaches, in a relatively large fraction of patients (3). Furthermore, not all patients under statin treatment achieve the target goal of LDLc reduction, and even when achieved, up to 40% of subjects continue suffering from cardiovascular events (5). In early 2000, proprotein convertase subtilisin/kexin type 9 (PCSK9), a protein associated with the turn-over of the low-density lipoprotein receptors, was identified as a promising target for hypercholesterolemia treatment (6). Currently, two groups of PCSK9 inhibitors are used in clinical practice: monoclonal antibodies (mAbs) such as evolocumab and alirocumab, and small interfering RNA (siRNA) such as inclisiran (7). However, the FDA approved anti-PCSK9 therapies only as an add-on to statin therapy, whereas the need to develop more cost-effective and safe-to-use cholesterol-lowering drugs still persists (8).

Regardless of the underlying mechanism of action, the efficacy and safety in late-stage trials of cholesterol-lowering compounds is assessed by the frequency of major adverse cardiac events (MACE) (9–13). The lack of a standardized combination of individual events comprising the MACE endpoint makes it vary from trial to trial. A three-point MACE outcome is the most commonly used composite endpoint and consists of nonfatal myocardial infarction (nfMI), nonfatal stroke (nfST), and cardiovascular mortality (CVM) (14). Some trials may also utilize a four-point MACE by including hospitalization for unstable angina (UA) or coronary revascularization procedures (CR) (14, 15). Less frequently, the composite MACE endpoint may consist of more than 4 components, with the inclusion of total (all cause) mortality (TM), coronary mortality (CM), transient ischemic attack (TIA), heart failure (HF), and other endpoints (16, 17). The use of composite endpoints is substantiated by the need to improve statistical efficiency and reduce the sample size of a trial, a challenge when considering the typically low rates in these events (18). However, MACE is also associated with several limitations. Firstly, introducing additional components to an endpoint inflates the resources required to measure it and complicates subsequent analyses and interpretation, since not all components have comparable importance vs. the clinical trial outcome (16). Moreover, individual components may follow diverging directionality within a single metric, masking the treatment benefit (19). Thus, finding the proper balance between statistical efficiency, clinical relevance and compatibility of components when deriving a composite endpoint such as MACE is essential for trial success and establishing patient benefits.

In the present work, we propose a model-informed approach for the optimization of MACE endpoint, based on a model-based meta-analysis of anti-hypercholesterolemia trials involving statin and anti-PCSK9 drugs. Using meta-regression modeling, we quantified the association between 15 individual MACE components and dyslipidemia biomarkers, based on 54 clinical trials of statins and PCSK9 inhibitors, which amounted to 270,471 subjects in total. The models and companion methodology we developed allow for evidence-based decision-making in choosing the appropriate composition of MACE endpoints, with respect to sample size, patient characteristics and therapy type in the trial.

## 2 Materials and methods

### 2.1 Study eligibility and data collection

A systematic literature search was performed in the ClinicalTrials.gov and PubMed databases, in accordance with PRISMA guidelines (20), to identify all randomized controlled trials reporting MACE and cholesterol measurements in populations with dyslipidemia treated either with statins or PCSK9 inhibitors (including anti-PCSK9 mAbs and anti-PCSK9 siRNA). Placebo and standard of care were used as a comparator arm. Clinical studies with different statins doses and similar background therapy in both arms were also included. The PRISMA checklist is available in the Supplementary Table S1. The following search terms were applied within the PubMed and ClinicalTrials.gov databases: (statin OR evolocumab OR alirocumab OR inclisiran) AND (cholesterol) AND (MACE) AND (randomized controlled trial). The exact queries are available in the Supplementary File S1. Two authors (A.V., V.S.) independently screened all abstracts and summaries of randomized controlled trials for eligibility. Disagreements were resolved through discussion between the two reviewers or arbitrated by a third reviewer. Records identified through systematic screenings were merged, and duplicates were then removed. If an abstract or summary was deemed valid for inclusion into the analysis, the original publication was investigated in detail and relevant content was added to the database. Studies with less than 100 participants per treatment group, or with a mean follow-up for cardiovascular events of less than 1.5 years were excluded from the analysis. Results were digitized only from publications presented in English language. References from the identified research papers and review articles were assessed to identify additional relevant manuscripts and reports (9, 21–26). The last updates to the collective database were implemented on June 28, 2022.

### 2.2 Outcome measures

Selected study reports and manuscripts were processed into a Microsoft Excel database with a pre-defined set of study design properties, population characteristics, and biomarker measurements (Supplementary Table S2). The following features of the study design were digitized: type of therapy (statins or anti-PCSK9), comparator type (active or SoC), dose and dose schedule in each cohort of a study, duration of a study follow-up. Population demographics were characterized by the prevention type, the presence of patients with severe renal disease, smoking status, proportion of diabetic patients, proportion of patients with hypertension, proportion of males, age, baseline measurements of body mass index (BMI), as well as baseline measurements of dyslipidemia biomarkers, including total cholesterol (TC), LDLc, high-density lipoprotein cholesterol (HDLс), triglycerides (TG), remnant cholesterol (remC) and non-high-density lipoprotein cholesterol (non-HDLc) in mg/dl. When prevention type was not explicitly indicated in the published sources, a mixed category was assigned to a study. Unknown values of TC, remC and non-HDLc were calculated based on Friedewald equation (27) using the following formulae:

$$TC=LDLc+HDLc+\frac{TG}{5},$$

remC=TC−HDLc−LDLc,

nonHDLc=TC−HDLc.

In addition, baseline- and comparator-adjusted measurements of TC (ΔTC), LDLc (ΔLDLc), HDLc (ΔHDLc), TG (ΔTG), remC (ΔremC) and non-HDLc (Δnon-HDLc) were introduced into the database as

$$\Delta \mathrm{Biom}=(\mathrm{Biom}-\mathrm{Bio}m_{\mathrm{BL}}{)}_{\mathrm{TRT}}-(\mathrm{Biom}-\mathrm{Bio}m_{\mathrm{BL}}{)}_{\mathrm{COMP}}.$$

where Biom [mg/dl] is the measurement of a biomarker at the latest available time point in a study; $\mathrm{Bio}m_{\mathrm{BL}}$ [mg/dl] is the baseline measurement of a biomarker; the TRT subscript denotes the values taken from the treatment arm; the COMP subscript represents the numbers from the comparator arm.

Missing covariate values (no more than 13% across all measurements, Supplementary Table S3) were imputed using the multiple imputation approach, assuming that the values were missing at random (28, 29). One hundred datasets were generated based on the method of multiple imputations by predictive mean matching, to account for the uncertainty and variability in the missing data (30). All subsequent inferences from the analysis were made based on a pooled evaluation of the sampled datasets.

Finally, we extracted the total number of subjects per trial arm and the number of either composite MACE or one of the following standalone events: mortality from different causes (TM, CM, CVM), stroke (ST) and its subtypes (non-fatal, fatal (fST), hemorrhagic (hST), ischemic (iST)), myocardial infarction (MI) and its subtypes [non-fatal, fatal (fMI)], and others (TIA, HF, UA, CR); 16 types of outcomes in total.

### 2.3 Meta-analysis and meta-regression

Risk ratios (RR) with 95% confidence intervals (CI) for single and composite MACE were calculated from the total number of events as well as the total number of subjects per arm per trial. The RR estimates were then used as dependent variables in the random-effects meta-analysis and meta-regression modeling (31).

The following key statistical metrics were evaluated per outcome, in addition to visual inspection of the funnel plots: percentage of variability across studies attributable to heterogeneity beyond chance (*I*^2^ statistic), as well as the risk and the direction of a publication bias estimated by the Egger test. Meta-regression models were developed in four successive steps on top of the base models with therapy type as a default predictor. First, forward covariate search was performed by sequentially introducing all available covariates (see Section 2.2) to the base model. Those covariates with a Wald test *p*-value <0.05 were then tested together in combinations with all possible interactions. Next, full models were developed by pooling the statistically significant covariates and interactions from the two previous steps into a single regression model. Finally, a backward covariate search was performed and the final model was selected based on the following five statistical and heuristic criteria: (i) same quality of the data description compared to the full model (Wald test, *p*-value <0.05), (ii) minimum number of covariates, (iii) significance of the model parameters, (iv) visual inspection of goodness-of-fit plots, and (v) biological interpretation of the results.

### 2.4 Sample size estimation based on the final meta-regression model

The proposed methodology for sample size calculation of clinical trials and associated optimization of MACE composition through the meta-regression modeling are based on the model-predicted average of the effect size, event frequency in the control group, and associated uncertainty, calculated for multiple stand-alone MACE components in a study with pre-defined duration and population.

The statistical significance of the treatment benefit relative to the control for the logarithm of RR is defined by the upper bound of the 95% CI for the meta-analysis average being less than zero. As 95% CI of the mean depends on the number of subjects in a trial, it is possible to estimate population size required to achieve statistical significance in the treatment effect for a particular outcome (see Supplementary File S2):

$$N(t)=1.96^{2}*\frac{2-4*k_{i}(t)*e^{\theta_{i}}+2*e^{\theta_{i}}}{k_{i}(t)*e^{\theta_{i}}*\theta_{i}^{2}},$$

where $\theta_{i}$ is the log(RR) estimate for the $i^{\mathrm{th}}$ event, and $k_{i}(t)$ is the proportion of patients who experienced the $i^{\mathrm{th}}$ event in the control arm that increases over time (Supplementary Figure S1).

Consequently, for the composite MACE, $\theta_{i}$ and $k_{i}(t)$ in the above equation were replaced by the following formulae (see also the Supplementary File S3): $\theta_{sum}(t)=log\left(\frac{\sum k_{i}(t)*e^{\theta_{i}}}{\sum k_{i}(t)}\right)$ and $k_{sum}(t)=\sum k_{i}(t)$, for the selected number and types of single events.

Furthermore, since both $\theta_{i}$ and $k_{i}(t)$ were derived from mathematical models, it was necessary to consider the uncertainty in these estimates when calculating N(t). As such, $\theta_{i}$ and $k_{i}(t)$ were sampled from the model-derived uncertainty distributions $\Theta_{i}∼N(\mu_{\theta_{i}},\sigma_{\theta_{i}}^{2})$ and $K_{i}(t)∼N(\mu_{k_{i}(t)},\sigma_{k_{i}(t)}^{2})$, where μ is the mean and σ is the standard error of the estimates. Taken together, the proposed method allowed to quantify the degree of confidence in the calculated N(t), for all types of in silico scenarios.

Data programming and processing were performed in the R software, version 4.0.2 (The R Foundation, Vienna, Austria). The meta-analysis and meta-regression modeling were performed in the metafor package, version 3.0.2 (32). The multiple imputation method was implemented using the mice package, version 3.14.0 (33).

## 3 Results

### 3.1 Study selection, patient population, trial characteristics, risk of bias

**Figure 1:**
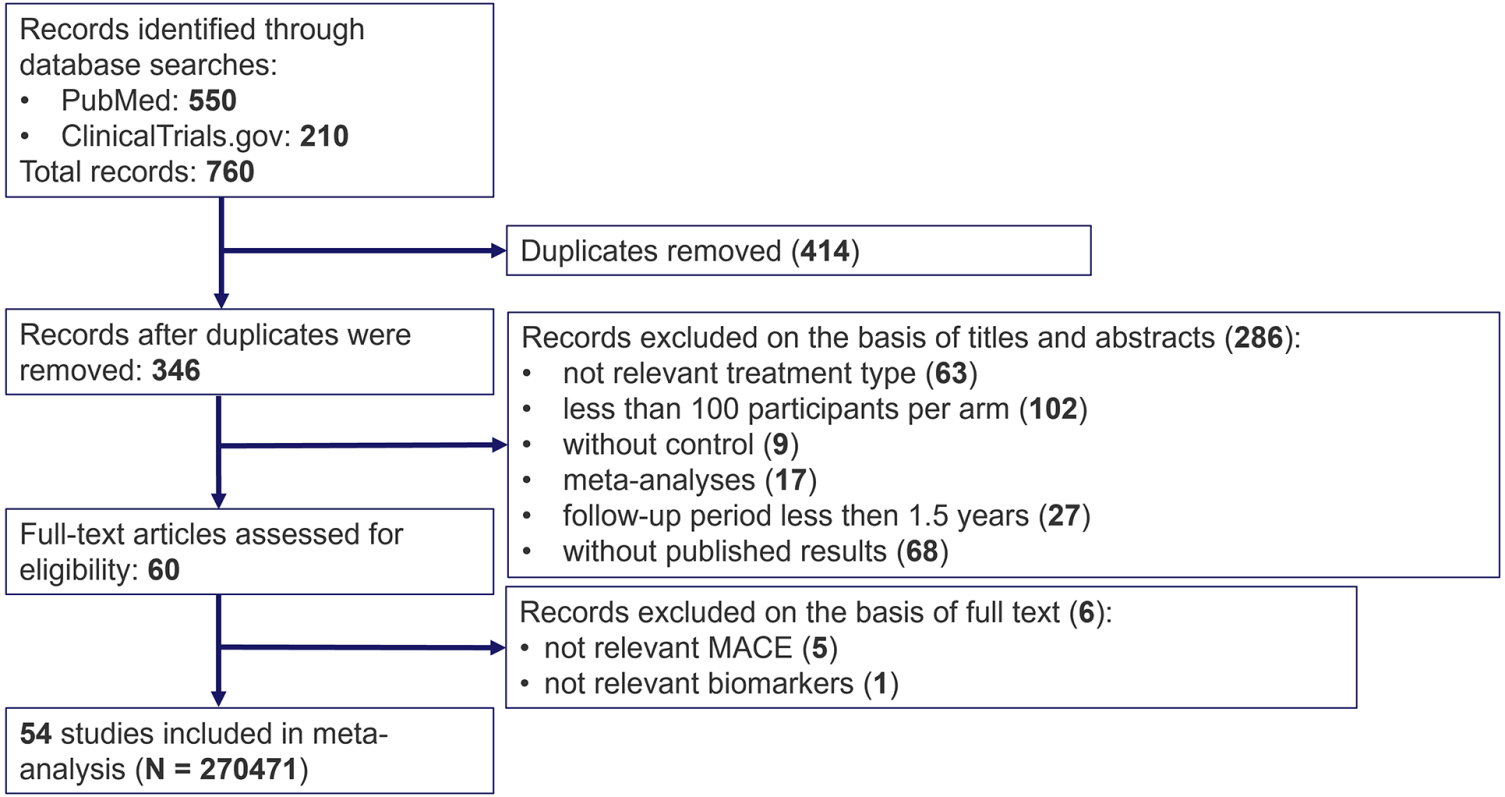
PRISMA flow diagram for search and selection of randomized clinical trials on cardiovascular outcomes under treatment with statins and PCSK9 inhibitors.

The systematic search in the PubMed and ClinicalTrial.gov databases resulted in 760 potentially relevant records of lipid-lowering clinical trials. 346 entries remained after removing duplicates. 286 studies were excluded based on the screening of titles and abstracts for the following reasons: irrelevant therapy (not statins or PCSK9 inhibitors), less than 100 patients per study arm, no comparator treatment, follow-up period of less than 1.5 years, no published results (for ClinicalTrial.gov), other meta-analyses or systematic reviews. 6 more manuscripts were discarded due to the absence of measurements of dyslipidemia biomarkers or MACE incidence after the review of the full text of the 60 eligible articles (references of the excluded articles can be requested from the corresponding author). As a result, 54 clinical trials fully satisfied the inclusion criteria and were used in the subsequent analyses. The flowchart for the selection and classification of articles is shown in Figure 1.

The final dataset comprised a total of 270,471 patients [mean age, 61.2 years; 30.3% women; mean baseline LDLc level of 136.6 mg/dl (3.53 mmol/L)]. A full summary of the included studies is available in the Supplementary Table S2. The database predominantly consists of clinical trials of statins (47 statin trials vs. 7 PCSK9 inhibitor trials). 15 trials included patients without a previous history of MACE, 21 trials were secondary prevention studies, and the remaining trials featured mixed patient population. 6 out of 54 trials included patients with severe renal disease. Baseline and demographic characteristics of patients, follow-up duration and treatment-mediated changes in dyslipidemia markers across all trials were assessed through a graphical analysis (Supplementary Figure S2). A strong correlation (Pearson's correlation coefficient >0.9) between baseline as well as treatment response in TC, non-HDLc and LDLc was observed. Study follow-up duration varied from 1.5 to 10 years, with a mean of 3.87 years. However, the association between the incidence of CV events and follow-up duration assessed by a weighted linear regression was marginally significant only for fMI (*p*-value = 0.028, *R*^2^ = 28%) (Supplementary Figure S3).

Publication bias, as evaluated through the visual inspection of funnel plots and quantified by the Egger test was significant (*p*-value <0.001) only for the composite MACE outcome (Supplementary Figure S4). Since publication bias interferes with the reliability of the results of a meta-analysis, composite endpoint was not considered in the next steps of the analysis. Finally, a series of leave-one-out diagnostics plots (34) revealed several outliers and potentially influential cases (Supplementary Figure S5), but their removal led to no substantial differences in the resulting effect size (data not shown).

### 3.2 Meta-analysis and meta-regression of treatment effects

**Figure 2:**
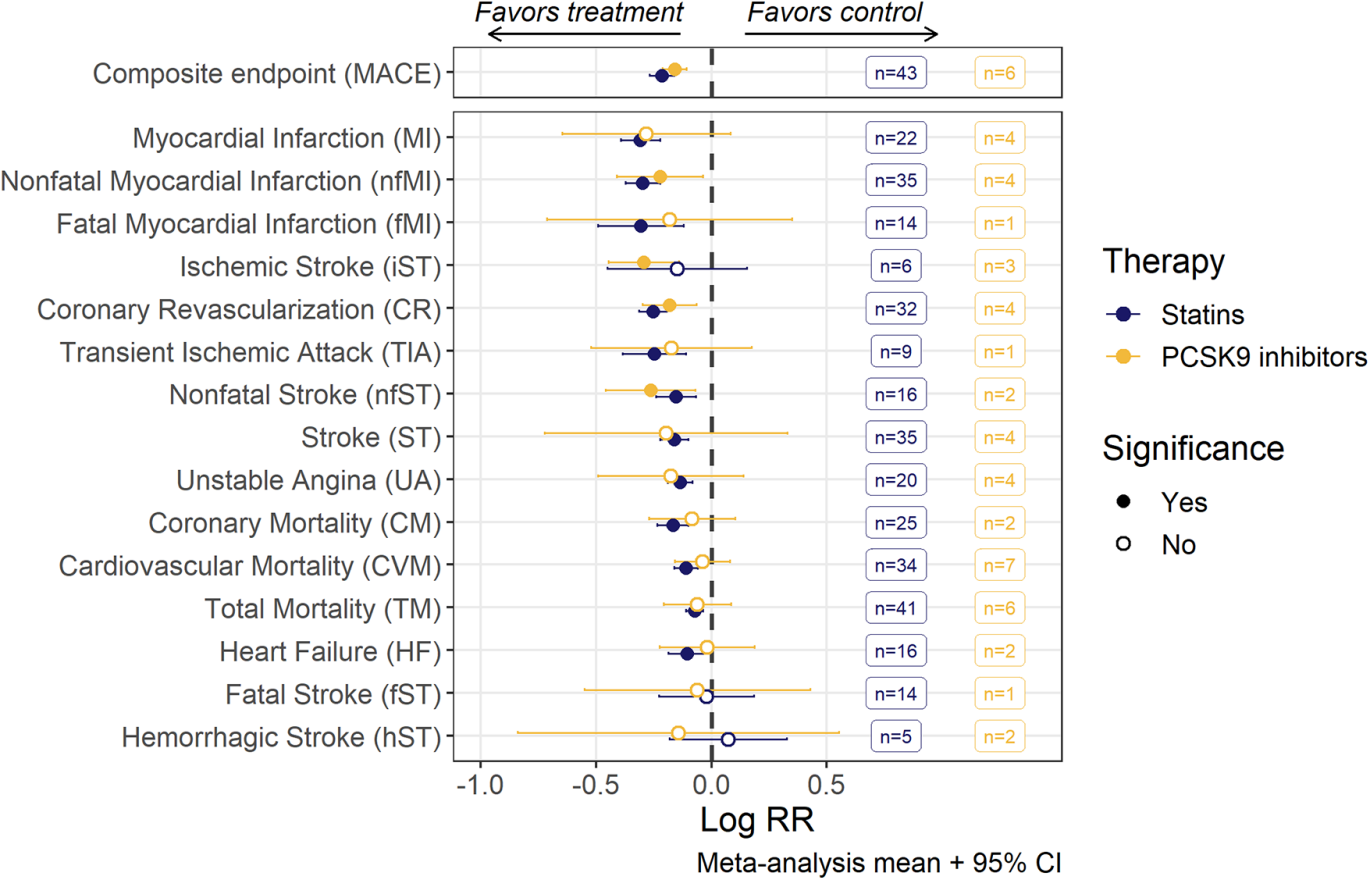
Risk ratios (RR; mean with 95% confidence intervals) of MACE and individual MACE components as assessed by random-effects meta-analysis modeling. Estimates for each endpoint were derived from a corresponding set of trials, the number of trials being given in the boxes. An open dot represents non-significant (*α* = 0.05) risk reduction between two groups. The vertical line depicts the point of “no difference” between two groups.

The pooled meta-analysis average of log(RR) divided between anti-PCSK9 and statin therapy for various CV events is shown in Figure 2. Point estimates of individual studies are presented in forest plots for each CV event (Supplementary Figure S6). Both types of hypercholesterolemia treatment significantly reduced the risks of composite MACE outcome. However, among the stand-alone events, statistical significance in the effect of anti-PCSK9 therapies was achieved only for 4 out of 15 outcomes (nfMI, iST, CR, nfST), whereas for statins, the reduction in the average risks was unambiguously determined for all components of MACE, except for iST, fST and hST. If the studies with patients with severe renal impairment were excluded—statistical significance was also achieved for iST risks reduction, although the effect on weighted means was negligible (Supplementary Figure S7).

We observe significant heterogeneity of the effect size (*I*^2^ up to 48%) among individual studies, as evaluated by Cochran's *Q*-test for 4 out of 15 single events: TM, iST, nfMI and CR. Nevertheless, random-effects meta-regression models were developed for all individual MACE components based on the workflow described in detail in section 2.3. As TC and non-HDLc exhibited strong correlation with LDLc (both at baseline and under treatment), they were excluded from the covariate search procedure. At least one covariate was deemed significant for 10 out of 15 CV endpoints (Table 1). Detailed results of the covariate search for each endpoint are presented in the Supplementary Table S4.

**Table 1:** Detailed information on models developed for each tested endpoint.

Event	Formula	β0 (SE)	β1 (SE)	β2 (SE)	β3 (SE)
CR	Log(RR) = β0 + β1_PCSK9 inhibitors_ + β2 × ΔLDLc	−0.035 (0.057) *p* = 0.55	**0.22 (0.067) *p* < 0.001**	**0.007 (0.002) *p* < 0.001**	
CVM	Log(RR) = β0 + β1_PCSK9 inhibitors_ + β2 × HDLc	**−0.625 (0.253) *p* = 0.02**	0.082 (0.067) *p* = 0.23	**0.012 (0.006) *p* = 0.05**	
HF	Log(RR) = β0 + β1_PCSK9 inhibitors_ + β2 × remC	**0.554 (0.253) *p* = 0.05**	0.097 (0.111) *p* = 0.4	**−0.023 (0.009) *p* = 0.03**	
iST	Log(RR) = β0 + β1_PCSK9 inhibitors_ + β2 × Hypertension	**−0.791 (0.17) *p* = 0.01**	−0.309 (0.122) *p* = 0.06	**0.011 (0.003) *p* = 0.02**	
MI	Log(RR) = β0 + β1_PCSK9 inhibitors_ + β2 × HDLc	0.407 (0.323) *p* = 0.22	0.033 (0.104) *p* = 0.75	**−0.017 (0.008) *p* = 0.04**	
ST	Log(RR) = β0 + β1_PCSK9 inhibitors_ + β2_Prevention_ × Age	−0.773 (0.403) *p* = 0.06	−0.056 (0.096) *p* = 0.56	*Primary:* 0.007 (0.007) *p* = 0.29	
				*Secondary:* 0.01 (0.006) *p* = 0.15	
				*Both:* 0.011 (0.006) *p* = 0.09	
TM	Log(RR) = β0 + β1_PCSK9 inhibitors_ + β2 × HDLc	**−0.448 (0.17) *p* = 0.01**	0.029 (0.063) *p* = 0.65	**0.008 (0.004) *p* = 0.03**	
CM	Log(RR) = β0 + β1_PCSK9 inhibitors_ + β2 × ΔLDLc	−0.019 (0.064) *p* = 0.77	0.147 (0.109) *p* = 0.19	**0.004 (0.002) *p* = 0.02**	
fST	Log(RR) = β0 + β1_PCSK9 inhibitors_ + β2_RD_	−0.113 (0.089) *p* = 0.23	0.05 (0.289) *p* = 0.87	**0.654 (0.283) *p* = 0.04**	
nfMI	Log(RR) = β0 + β1_PCSK9 inhibitors_ + β2 × ΔLDLc + β3_RD_ × ΔLDLc	−0.057 (0.091) *p* = 0.54	**0.242 (0.1) *p* = 0.02**	**0.008 (0.003) *p* < 0.001**	**−0.005 (0.002) *p* = 0.02**

Model coefficients are presented as point estimates with standard error. β0 indicates the impact of statins therapy, β1_PCSK9 inhibitors_ represents the impact of PCSK9 inhibitors relative to statins, β2_Prevention_ represents the effect of different prevention categories, β3_RD_ represents the effect of inclusion of patients with severe renal disease.

Statistically significant values (*p* < 0.05) are in bold.

For all CV endpoints with the exception of CR and nfMI, the difference in effect size between statins and anti-PCSK9 therapies was not significant. For CR and MI, mean treatment benefit was decreased, respectively, by 24.6% [95% CI, 9.3–42.1] and 27.4% [95% CI, 4.7–55] in anti-PCSK9 treated populations.

Treatment-mediated changes in LDLc concentration were established as predictors of RR reduction for nfMI, CR and CM. 1 mmol/L (38.67 mg/dl) decrease in LDLc level was associated with RR changes of −24% [95% CI, −34% to −11%] for CR, −14% [95% CI, −26%–0%] for CM, and −27% [95% CI, −34% to −11%] for nfMI. The abundance of patients with severe renal disease in a trial negatively affected the treatment benefit for the latter by diminishing the respective decrease in RR to −11% [95%CI, −39%–30%].

Baseline levels of dyslipidemia biomarkers were found to be associated with the RR of HF, MI, TM, and CVM. In particular, an increase of 13 mg/dl in baseline remC resulted in an approximately 2-fold decrease in the RR of HF. Likewise, high baseline HDLc positively correlated with treatment benefit in MI. In contrast, populations with baseline HDLc above 48 mg/dl for statin treatment and at any investigated values of covariate (36–60 mg/dl) for PCSK9 inhibitors were shown to lack any significant response in RR of TM and CVM.

Quantitative relationships for stroke-related events, including ST, iST and fST, were established only with the general demographics of a study population, such as prevention category, renal status, age, and proportion of subjects with hypertension. RR reduction of iST in a population with 75% subjects experiencing high blood pressure was 1.7 [95% CI, 1.3–2.3] fold less effective compared to a population with 25% hypertensive patients. Likewise, the effect of the therapy on fST incidence was notably less explicit [by 1.9 (95% CI, 1.1–3.3) times] in subjects with late stage renal disease, as compared to patients with normal renal status. Finally, the RR of unspecified ST was found to be dependent on the interaction between prevention category and mean age of the trial population, with age negatively correlating with the effect size of the treatment. The shift from the primary prevention category to secondary for the population with the same age of 60 years resulted in the RR changing from 0.71 [95% CI, 0.62–0.82] to 0.82 [95% CI, 0.76–0.88] for statin and from 0.67 [95% CI, 0.53–0.85] to 0.78 [95% CI, 0.65–0.93] for PCSK9 trials.

### 3.3 Model-based optimization of MACE endpoint composition

**Figure 3:**
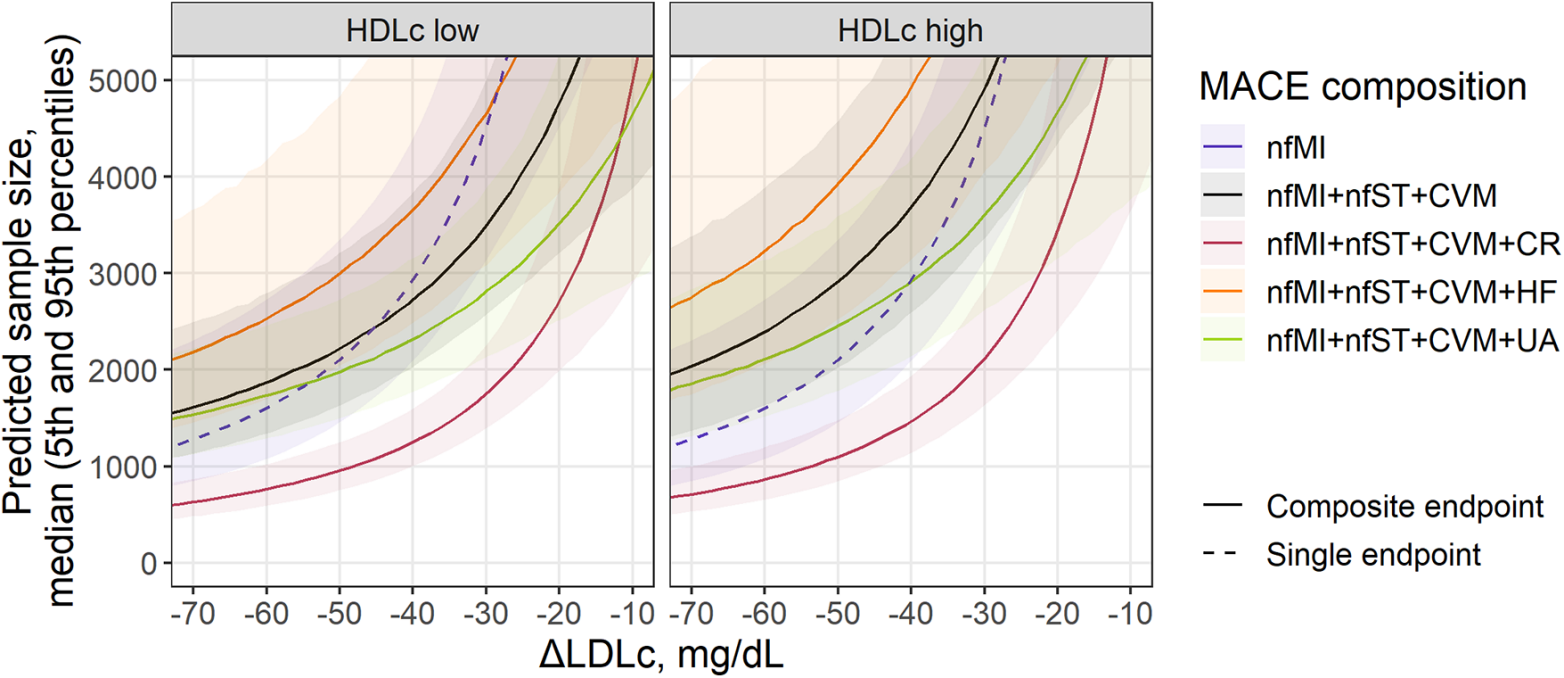
Sample size prediction for a statin treatment trial with single (dashed) or composite MACE (solid) as an endpoint. Predicted sample size for MACE composition depends on covariates found as significant in the meta-regression analysis of individual components: treatment-mediated decrease in LDLc (nfMI, CR), inclusion of patients with renal disease (nfMI) and baseline level of HDLc (CVM) and remC (HF). Sample size is presented as median (solid line) with 5th-95th percentiles (shaded area) for a statin trial without inclusion of patients with renal disease. Facets represent HDLc status of patients and correspond to the 25th and 75th percentiles of covariate value in the investigated population (41.4 and 49.9 mg/ml, respectively). For MACE composition with HF inclusion, baseline remC level (27.7 mg/ml) was derived from the multivariate distribution of HDLc and remC. Follow-up duration corresponds to the mean observed value across investigated studies (3.87 years).

The meta-regression models we developed in conjunction with the methodology for sample size calculation described in Section 2.4 provided a quantitative tool for the benchmarking of various MACE endpoint compositions across different types of therapies while considering demographic characteristics and length of the studies. To illustrate the application of such a tool, we predicted the sample size required to achieve statistical significance in the effect size (RR) of a statin treatment for different combinations of 3- and 4-point MACE endpoints, and compared it to nfMI as the single component with the most prominent effects in RR reduction, as illustrated in Figure 2. Simulations were performed for a wide range of treatment-mediated changes in LDLc (from −10 to −75 mg/dl) for a study with a 4-year follow-up, in a population without renal impairment and with low or high baseline HDLc defined as, respectively, 41.4 and 49.9 mg/ml, which corresponded to the 25% and 75% percentiles of the observed data (Figure 3).

All curves in Figure 3 declined exponentially following an increase in LDLc response to the treatment, as it correlated with the RR of events such as nfMI and CR. Consequently, to achieve statistical significance in the effect size for nfMI alone, a total of 1,164 subjects [5%–95% percentile range, 765–2,139] would be required, provided the expected magnitude of LDLc reduction relative to the comparator arm is −75 mg/dl, whereas for an ΔLDLc reduction of −30 mg/dl, the number of subjects would need to be drastically increased up to 4,564 [5%–95% percentile range, 3,163–7,122]. If the effect size associated with the LDLc treatment response and other characteristics of the population is too small or the study duration is too short, the number of subjects required to reliably estimate the treatment benefit in an outcome will asymptotically tend to infinity. The steepness of the curve, however, will depend on the event or event combination. For example, for the commonly used 3-point MACE, consisting of nfMI, nfST and CVM, the recommended population size at −30 mg/dl LDLc reduction is 3,515 [5%–95% percentile range, 2,585–5,038] and 4,946 [5%–95% percentile range, 3,374–7,921] for patients with HDL low and high status, respectively, which is by comparison less than that for nfMI, but only for the patients with low HDL. Introducing additional CV events to the 3-point MACE does not necessarily lead to a reduction in the required sample size and depends on both the average effect size of an event and the uncertainty around its estimate. According to our model-based predictions, accounting for UA and HF did not provide significant benefit in enrollment requirements. The most efficient 4-point MACE in terms of population size was the combination of nfMI, nfST, CVM, and CR. For this type of an outcome, for a study in patients with low baseline HDL, a minimum required sample size was estimated to be between 685 [5%–95% percentile range, 524–935] and 1,491 [5%–95% percentile range, 1,195–1,904] for a ΔLDLc between −65 and −35 mg/dl.

**Figure 4:**
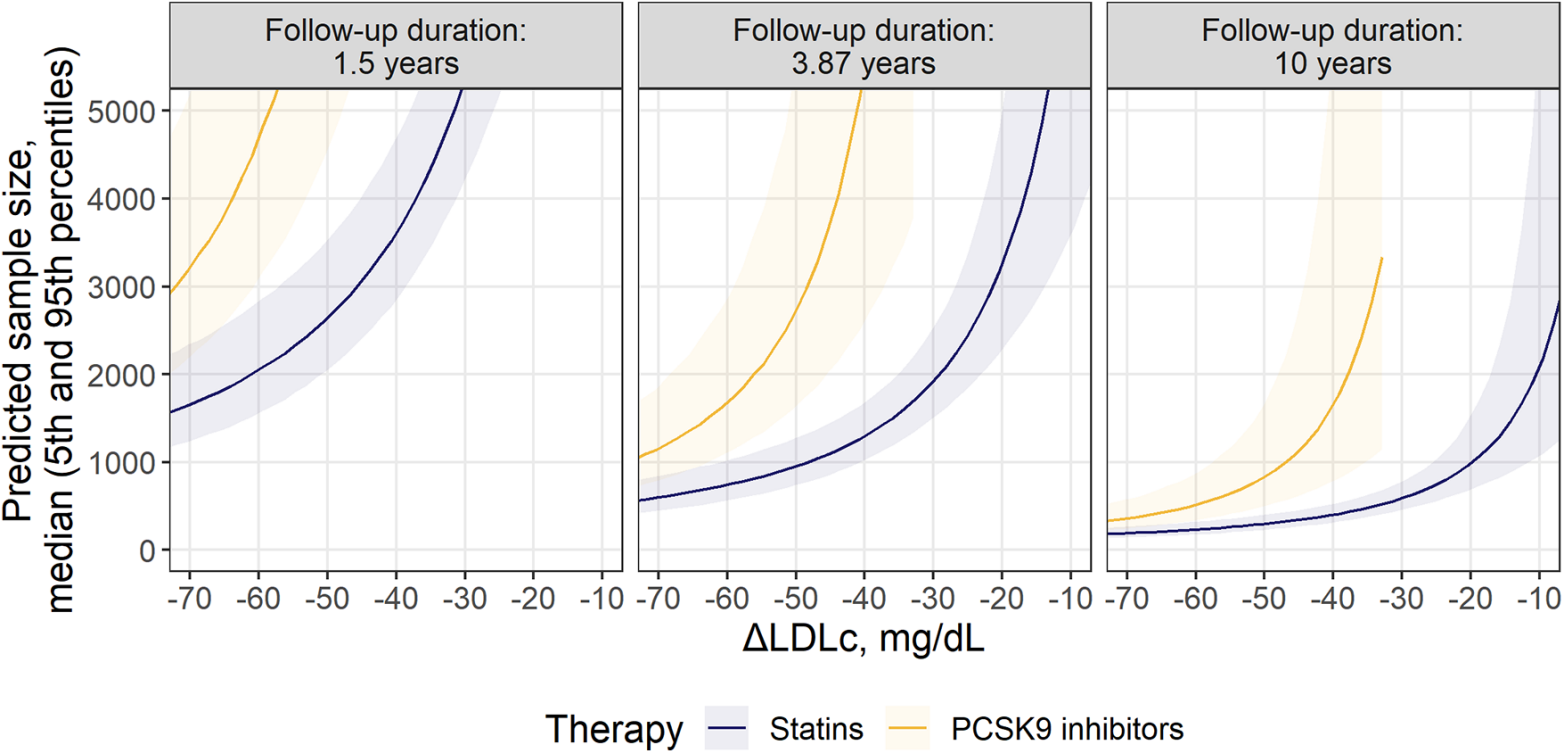
Comparison of required sample size for statins and PCSK9 inhibitors within the selected MACE composition: nfMI + nfST + CR. Predicted sample size is presented as median (solid line) with 5th-95th percentiles (shaded area) for patients without significant renal disease. Facets represent minimum, mean and maximum observed values of follow-up durations across investigated studies.

Finally, we compared the minimal recommended population size for statin and anti-PCSK9 trials, to highlight the opportunity to benchmark the effectiveness of different outcomes across therapies with diverse mechanisms of action at different follow-up duration, using the proposed methodology (Figure 4). A 3-point MACE consisting of nfMI, nfST and CR was chosen for the task, since these endpoints only showed statistically significant treatment benefit in RR for both classes of compounds, as follows from Figure 2. Study duration was chosen to be 1.5, 4.0 and 10 years. As expected, an increase in follow-up duration notably alleviates the sample size requirement: in the extreme case of a 10-year long study with an average LDLc response of −75 mg/dl, the necessary enrollment varied between 175 [5%–95% percentile range, 131 to 249] and 309 [5%–95% percentile range, 215–488] for both statins and anti-PCSK9 therapies. However, the predicted sample size for anti-PCSK9 compounds was more sensitive to ΔLDLc changes, as compared to statins. As a result, at 4.0-year follow-up and with a −50 mg/dl ΔLDLc, the estimated sample size was 2,720 [5%–95% percentile range, 1,628–5,530] for anti-PCSK9 therapies and 953 [5%–95% percentile range, 738–1,276] for statins. Furthermore, according to model predictions, clinical studies of PCSK9 inhibitors would require ΔLDLc to be at least −32 mg/dl. Otherwise, no statistically significant difference from the control in the selected composite outcome would be observed for the therapy.

## 4 Discussion

Composite clinical endpoints are essential therapy assessment tools in trial design optimization in various disease areas, offering both advantages and disadvantages associated with the choice of individual events which make up the endpoint (16). In the present study, we proposed a model-based methodology for the selection of an optimal repertoire of events for a composite endpoint, based on a meta-regression of historical data of anti-hypercholesterolemia therapies, including statins as well as anti-PCSK9 mAbs and siRNA.

Whereas multiple meta-analyses quantifying the effect size of a treatment and of associated intrinsic and extrinsic factors exist in this field (26, 35, 36), the current research represents, to our knowledge, the first successful attempt to evaluate and specify the relationships between multiple early-phase biomarkers and long-term efficacy outcomes in dyslipidemia treatment under statin or anti-PCSK9 therapies, in the context of 15 individual MACE components, and based on an up-to-date comprehensive analysis of historical data from 54 clinical trials. Furthermore, the described methodology and associated workflow can be applied beyond this particular therapeutic area and may serve as a quantitative tool for sample size calculation of future trials in other indications.

Treatment-mediated decrease in LDLc is a well-known factor associated with the risk reduction of composite MACE endpoints (37). According to our results, a robust association between changes in LDLc over the treatment period and the RR was established only for 3 out of 15 events: nfMI, CR and CM. Nonetheless, ΔLDLc was not the sole predictor identified for the endpoints: baseline levels of lipoprotein cholesterol, hypertension and other demographic characteristics affected the relative risks of HF, MI, TM, CVM, and different types of stroke. Overall, RR reduction in healthier subjects was less evident compared to that in the patients with poor dyslipidemia profile, which is in line with the general knowledge. For example, high remC concentration in the population at baseline was associated with an increased treatment benefit in HF relative to control, while it is also known that an abnormally high remC concentration in plasma represents a risk factor for HF occurrence (38, 39). Interestingly, the abundance, at baseline, of HDLc, the “good cholesterol”, was not indicative of an increased treatment benefit for TM and CVM, unlike for MI, which can be explained by the intricate physiological network of lipoprotein homeostasis (40–43). Finally, statistically significant differences in RR reduction between anti-PCSK9 and statin treatments after adjusting for confounding factors was established only for CR and nfMI, which might be explained by the pleiotropic effects of statins that include improvement in endothelial function, enhancement of the stability of atherosclerotic plaques, decreased oxidative stress and inflammation, and inhibition of the thrombogenic response (44). RR were not associated with trial duration, as investigated by an exploratory data analysis and a covariate search (Supplementary Figure S3). In consideration of all the above, it is important to note that the present analysis was based on aggregated study-level data from heterogenous populations, a majority of them having been treated with statins (47 out of 54 trials) and included participants with normal renal function only (48 out of 54 studies). Moreover, the evaluated studies were conducted over a span of 30 years, which affected rescue and background therapy as well as detection methods, and may account for some of the differences in absolute event rate between the trials. A sensitivity analysis was performed to test the effect of renal impairment on the RR of each CV outcome. No statistically significant difference in average risks was observed between the subset of studies with participants with normal renal function and the complete set of trials (Supplementary Figure S7). An additional evaluation was performed to highlight the lack of correlation between incidence of CV events and year of publishing the results (Supplementary Figure S8).

In our proposed method, meta-regression of the effect size for single events is the founding step for the optimization of composite endpoints. Contrary to a popular belief, the inclusion of additional outcomes does not necessarily result in an increase in power (45). The effect size for a composite endpoint as measured by the RR is represented by the weighted average of the treatment benefit per incorporated single event (see Methods, Section 2.4). As such, introducing additional outcomes with a lower effect size than that in the previously included components does not provide an extra gain in power; moreover, a small decrease in RR for one event can dilute or mask a stronger effect elsewhere. The confidence in the RR estimate depends on the total number of events within the follow-up period. Thus, the sample size of the population required to achieve a statistically significant effect size for a composite endpoint is derived from the delicate balance between the effect sizes of the underlying events (which in turn depend on dyslipidemia markers and population demographics), their frequencies, and the study duration. In our methodology, the former is derived from the meta-regression modeling, and the latter is defined by the researcher. The probability of events is derived from the control arms and is assumed to be constant over time (Supplementary Figure S1), although introducing non-linear dynamics to the event frequency might improve the precisions of the sample size calculation (46, 47). Considering the above, it is expected to observe a notable reduction in sample size requirements when a frequently occurring CR outcome with marked effect size is introduced to the commonly used 3-point MACE (Figure 3). In contrast, inclusion of HF or UA with relatively low effect size and moderate frequency has no substantial effect on composite MACE. Finally, since the method considers uncertainty in the estimated meta-regression means, the respective uncertainty around the sample size predictions will shrink as the number and size of the underlying trials will grow, which explains the larger spread in the predicted percentiles for the anti-PCSK9 treatments, as compared to statins, in Figure 4.

In summary, we proposed a methodology for the optimization of the composition of complex clinical endpoints following the model-based meta-analysis of anti-hypercholesterolemia trials, based on the sample size estimation required to achieve statistical significance in the RR reduction and as defined by the upper bound of the 95% CI of the weighted mean being less than zero. The approach takes into account the uncertainty in model parameters, the frequency of events in the control arms, the type of therapy, population demographics, baseline- and treatment-related markers, and can be useful in a variety of situations and indications. It can be applied to support the design of future cholesterol-lowering drugs, especially anti-PCSK9 therapies, since there are currently many such compounds out of various pharmacological modalities under development (small molecules (48), monoclonal antibodies (49), antisense oligonucleotides, siRNAs (50), CRISPR/Cas9 gene editing systems (51), polypeptides (52), vaccines (53)). Overall, rather than standardizing MACE composition, it would be preferable to redefine composite outcome for each population and mechanism of action, in order to achieve greater power. Alternatively, the method can be used to tune the endpoint composition for novel compounds in other disease areas, once the data on single outcomes for a first-in-class drug are available, making the development of further compounds, out of novel pharmacological modalities, more efficient.

## Data Availability

The original contributions presented in the study are included in the article/Supplementary Material, further inquiries can be directed to the corresponding author.
