# Supplementary material for: Optimization of the MACE endpoint composition to increase power in studies of lipid-lowering therapies—a model-based meta-analysis
Source: Front Cardiovasc Med. 2024 Jan 8;10:1242845. doi: 10.3389/fcvm.2023.1242845 (PMC10832431; doi:10.3389/fcvm.2023.1242845)

Supplementary Material

Optimization of the MACE endpoint composition to increase power in studies of lipid-lowering therapies - A model-based meta-analysis

**Alina Volkova^*^, Boris Shulgin, Gabriel Helmlinger, Kirill Peskov, Victor Sokolov**

*** Correspondence:** Alina Volkova, Alina.Volkova@msdecisions.tech

# Supplementary File 1

**Search strategy**

Final query for search in PubMed database:

(((statin) OR (evolocumab OR alirocumab OR bococizumab) OR (inclisiran)) AND (cholesterol) AND ("stroke" OR "infarction" OR "revascularization" OR "MACE") AND ("randomized controlled trial"[Publication Type])) AND (cardi*) AND (english[Filter])

Final query for search in ClinicalTrial.gov database:

Atorvastatin OR Fluvastatin OR Lovastatin OR Pitavastatin OR Pravastatin OR Rosuvastatin OR Simvastatin OR Mevastatin OR Inclisiran OR Evolocumab OR Alirocumab OR Bococizumab | Completed, Terminated, Unknown status Studies | cardiovascular OR coronary OR stroke OR infarction OR angina OR atherosclerosis OR dyslipidemia OR cholesterol | cholesterol OR lipid OR LDL-c OR mortality OR death OR stroke OR infarction OR MACE OR cardiovascular OR safety | Adult, Older Adult | Phase 2, 3, 4, Not Applicable | Enrollment >= 200 | Completion.Date - Start.Date > 2

# Supplementary File 2

**Sample size** $\boldsymbol{N}$ **estimation for a new virtual trial with known effect size (**$\boldsymbol{\theta}_{\boldsymbol{i}}$**) and known risks in the control arm (**$\boldsymbol{k}_{\boldsymbol{i}}$**).**

For a new virtual trial, assign the following 2x2 event table, assuming equal number of patients across arms ($\frac{\boldsymbol{N}}{\mathbf{2}}$):

|  | Event | No event | Total |
| --- | --- | --- | --- |
| Control group | $\boldsymbol{c}$ | $\boldsymbol{d}$ | $\boldsymbol{c}+\boldsymbol{d}=\frac{\boldsymbol{N}}{\mathbf{2}}$ |
| Treatment group | $\boldsymbol{a}$ | $\boldsymbol{b}$ | $\boldsymbol{a}+\boldsymbol{b}=\frac{\boldsymbol{N}}{\mathbf{2}}$ |

Then, the risks in the control arm $\boldsymbol{k}_{\boldsymbol{i}}$ could be derived as $\boldsymbol{k}_{\boldsymbol{i}}= \frac{\boldsymbol{c}}{\frac{\boldsymbol{N}}{\mathbf{2}}}$ and subsequently

$\left\{ \begin{aligned} \boldsymbol{c}=\boldsymbol{k}_{\boldsymbol{i}}*\frac{\boldsymbol{N}}{\mathbf{2}} \\ \boldsymbol{d}=\frac{\boldsymbol{N}}{\mathbf{2}}-\boldsymbol{k}_{\boldsymbol{i}}*\frac{\boldsymbol{N}}{\mathbf{2}} \end{aligned} \right.$ (1)

The effect size of the trial could be estimated via risk ratios (RR) in the treatment and control arms (note that different metrics for sample size could be used):

$$\boldsymbol{\theta}_{\boldsymbol{i}}=\boldsymbol{log}(\boldsymbol{RR})={\boldsymbol{log}(\frac{\boldsymbol{a}}{\frac{\boldsymbol{N}}{\mathbf{2}}\text{ }}}/{\frac{\boldsymbol{c}}{\frac{\boldsymbol{N}}{\mathbf{2}}\text{ }}})=\boldsymbol{log}(\frac{\boldsymbol{a}}{\boldsymbol{c}})$$

Then,

$\left\{ \begin{aligned} \boldsymbol{a}=\boldsymbol{c}*\boldsymbol{e}^{\boldsymbol{\theta}_{\boldsymbol{i}}}=\boldsymbol{k}_{\boldsymbol{i}}*\frac{\boldsymbol{N}}{\mathbf{2}}*\boldsymbol{e}^{\boldsymbol{\theta}_{\boldsymbol{i}}} \\ \boldsymbol{b}=\frac{\boldsymbol{N}}{\mathbf{2}}-\boldsymbol{k}_{\boldsymbol{i}}*\frac{\boldsymbol{N}}{\mathbf{2}}*\boldsymbol{e}^{\boldsymbol{\theta}_{\boldsymbol{i}}} \end{aligned} \right.$ (2)

The minimal required sample size $\boldsymbol{N}$ for a new trial is determined by the 97.5th percentile of the effect size being less than the value of the effect size with no difference between treatment and control (i.e., $\log\left( RR \right)=0$ in our case):


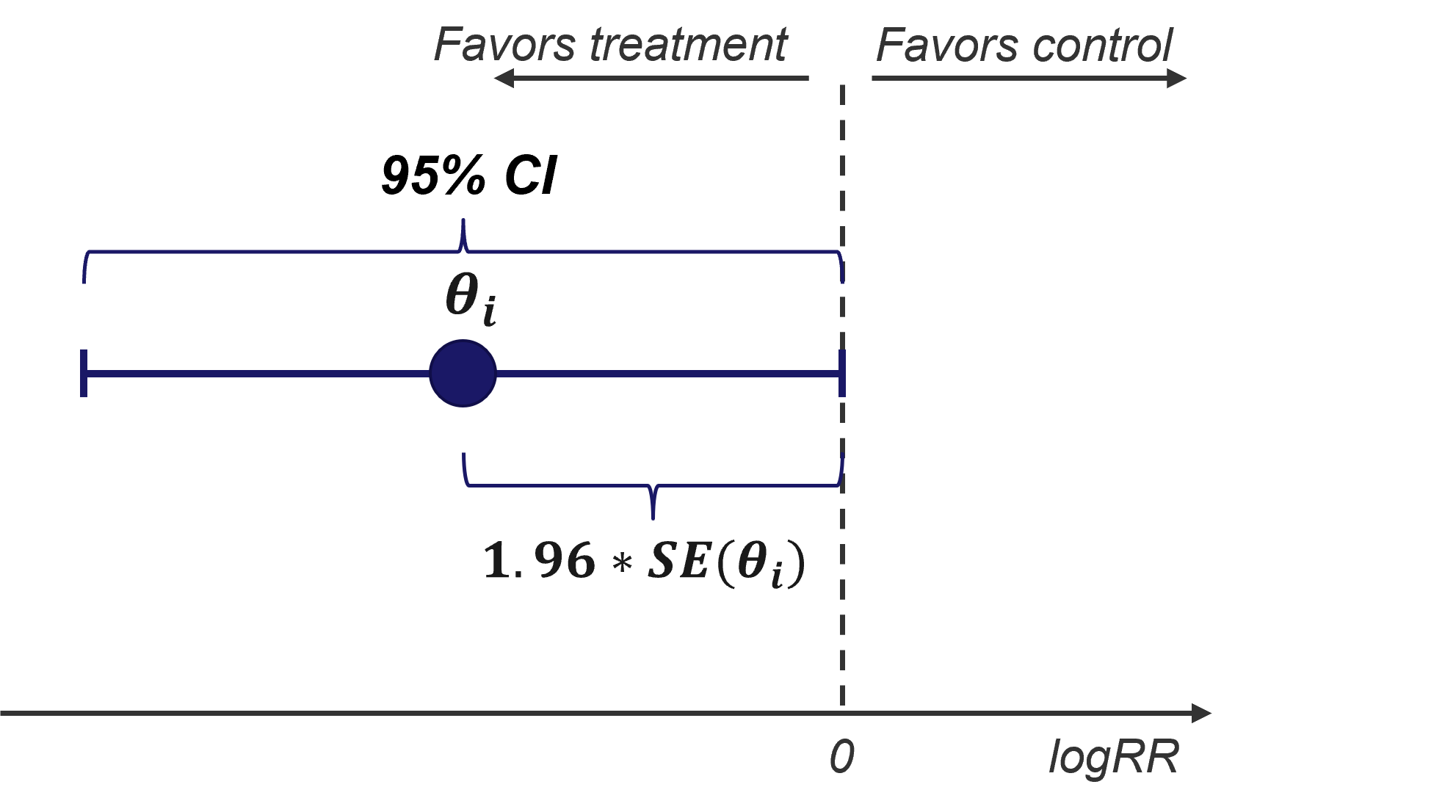


Therefore, at a minimal sample size $\boldsymbol{N}$ the following equation must be satisfied:

$${\mathbf{1}.\mathbf{96}*\boldsymbol{SE}(\boldsymbol{\theta}}_{\boldsymbol{i}})+\boldsymbol{\theta}_{\boldsymbol{i}}=\mathbf{0}$$

Substitute SE for the effect size as $\boldsymbol{log}(\boldsymbol{RR})$ based on the 2x2 event table:

$$\mathbf{1}.\mathbf{96}*\sqrt{\frac{\mathbf{1}}{\boldsymbol{a}}-\frac{\mathbf{1}}{\boldsymbol{a}+\boldsymbol{b}}+\frac{\mathbf{1}}{\boldsymbol{c}}-\frac{\mathbf{1}}{\boldsymbol{c}+\boldsymbol{d}}}+\boldsymbol{\theta}_{\boldsymbol{i}}=\mathbf{0}$$

Replace $\boldsymbol{a}$, $\boldsymbol{b}$, $\boldsymbol{c}$ and $\boldsymbol{d}$ with (1) and (2):

$$\mathbf{1}.\mathbf{96}*\sqrt{\frac{\mathbf{1}}{\boldsymbol{k}_{\boldsymbol{i}}*\frac{\boldsymbol{N}}{\mathbf{2}}*\boldsymbol{e}^{\boldsymbol{\theta}_{\boldsymbol{i}}}}-\frac{\mathbf{2}}{\boldsymbol{N}}+\frac{\mathbf{1}}{\boldsymbol{k}_{\boldsymbol{i}}*\frac{\boldsymbol{N}}{\mathbf{2}}}-\frac{\mathbf{2}}{\boldsymbol{N}}}=-\boldsymbol{\theta}_{\boldsymbol{i}}$$

Then, calculate $\boldsymbol{N}$:

$\mathbf{1}.\mathbf{96}*\sqrt{\frac{\mathbf{1}}{\boldsymbol{k}_{\boldsymbol{i}}*\frac{\boldsymbol{N}}{\mathbf{2}}*\boldsymbol{e}^{\boldsymbol{\theta}_{\boldsymbol{i}}}}-\frac{\mathbf{2}}{\boldsymbol{N}}+\frac{\mathbf{1}}{\boldsymbol{k}_{\boldsymbol{i}}*\frac{\boldsymbol{N}}{\mathbf{2}}}-\frac{\mathbf{2}}{\boldsymbol{N}}}=-\boldsymbol{\theta}_{\boldsymbol{i}}\to{\mathbf{1}.\mathbf{96}}^{\mathbf{2}}*\frac{\mathbf{2}-\mathbf{4}*\boldsymbol{k}_{\boldsymbol{i}}*\boldsymbol{e}^{\boldsymbol{\theta}_{\boldsymbol{i}}}+\mathbf{2}*\boldsymbol{e}^{\boldsymbol{\theta}_{\boldsymbol{i}}}}{\boldsymbol{k}_{\boldsymbol{i}}*\boldsymbol{N}*\boldsymbol{e}^{\boldsymbol{\theta}_{\boldsymbol{i}}}}= \boldsymbol{\theta}_{\boldsymbol{i}}^{\mathbf{2}}\to$

$$\to{\boldsymbol{N}=\mathbf{1}.\mathbf{96}}^{\mathbf{2}}*\frac{\mathbf{2}-\mathbf{4}*\boldsymbol{k}_{\boldsymbol{i}}*\boldsymbol{e}^{\boldsymbol{\theta}_{\boldsymbol{i}}}+\mathbf{2}*\boldsymbol{e}^{\boldsymbol{\theta}_{\boldsymbol{i}}}}{\boldsymbol{k}_{\boldsymbol{i}}*\boldsymbol{e}^{\boldsymbol{\theta}_{\boldsymbol{i}}}*\boldsymbol{\theta}_{\boldsymbol{i}}^{\mathbf{2}}}$$

# Supplementary File 3

**Estimation of the effect size** $\boldsymbol{\theta}_{\boldsymbol{sum}}$ **and the risks in the control group** $\boldsymbol{k}_{\boldsymbol{sum}}$ **for a new virtual trial with composite MACE as an endpoint, based on known effect sizes** $\boldsymbol{\theta}_{\boldsymbol{i}}$ **and the risks in the control group** $\boldsymbol{k}_{\boldsymbol{i}}$ **for each** $\boldsymbol{i}$**th component of MACE.**

For a new virtual trial, assign the following 2x2 event table, assuming equal number of patients across arms ($\frac{\boldsymbol{N}}{\mathbf{2}}$):

|  | Event | No event | Total |
| --- | --- | --- | --- |
| Control group | $\boldsymbol{c}$ | $\boldsymbol{d}$ | $\boldsymbol{c}+\boldsymbol{d}=\frac{\boldsymbol{N}}{\mathbf{2}}$ |
| Treatment group | $\boldsymbol{a}$ | $\boldsymbol{b}$ | $\boldsymbol{a}+\boldsymbol{b}=\frac{\boldsymbol{N}}{\mathbf{2}}$ |

Assign analogous tables for each $\boldsymbol{i}$th component of MACE:

|  | Event | No event | Total |
| --- | --- | --- | --- |
| Control group | $\boldsymbol{c}_{\boldsymbol{i}}$ | $\boldsymbol{d}_{\boldsymbol{i}}$ | $\boldsymbol{c}_{\boldsymbol{i}}+\boldsymbol{d}_{\boldsymbol{i}}=\frac{\boldsymbol{N}}{\mathbf{2}}$ |
| Treatment group | $\boldsymbol{a}_{\boldsymbol{i}}$ | $\boldsymbol{b}_{\boldsymbol{i}}$ | $\boldsymbol{a}_{\boldsymbol{i}}+\boldsymbol{b}_{\boldsymbol{i}}=\frac{\boldsymbol{N}}{\mathbf{2}}$ |

For $\boldsymbol{\theta}_{\boldsymbol{i}}$ and $\boldsymbol{k}_{\boldsymbol{i}}$, use the following formulae:

$$\left\{ \begin{aligned} \boldsymbol{\theta}_{\boldsymbol{i}}=\boldsymbol{log}(\boldsymbol{RR})={\boldsymbol{log}(\frac{\boldsymbol{a}_{\boldsymbol{i}}}{\frac{\boldsymbol{N}}{\mathbf{2}}}}/{\frac{\boldsymbol{c}_{\boldsymbol{i}}}{\frac{\boldsymbol{N}}{\mathbf{2}}}})=\boldsymbol{log}(\frac{\boldsymbol{a}_{\boldsymbol{i}}}{\boldsymbol{c}_{\boldsymbol{i}}}) \\ \boldsymbol{k}_{\boldsymbol{i}}=\frac{\boldsymbol{c}_{\boldsymbol{i}}}{\frac{\boldsymbol{N}}{\mathbf{2}}} \end{aligned} \right.\to\left\{ \begin{aligned} \boldsymbol{a}_{\boldsymbol{i}}=\boldsymbol{c}_{\boldsymbol{i}}*\boldsymbol{e}^{\boldsymbol{\theta}_{\boldsymbol{i}}} \\ \boldsymbol{c}_{\boldsymbol{i}}=\boldsymbol{k}_{\boldsymbol{i}}*\frac{\boldsymbol{N}}{\mathbf{2}} \end{aligned} \right.$$

Assume that the number of patients who experience several individual MACE within the trial is negligible, thus we can calculate the number of MACE as the sum of individual MACE components:

$$\left\{ \begin{aligned} \boldsymbol{c}=\sum\boldsymbol{c}_{\boldsymbol{i}} \\ \boldsymbol{a}=\sum\boldsymbol{a}_{\boldsymbol{i}} \end{aligned} \right.$$

Then calculate $\boldsymbol{\theta}_{\boldsymbol{sum}}$:

$$\boldsymbol{\theta}_{\boldsymbol{sum}}=\boldsymbol{log}(\boldsymbol{RR})={\boldsymbol{log}(\frac{\boldsymbol{a}}{\frac{\boldsymbol{N}}{\mathbf{2}}}}/{\frac{\boldsymbol{c}}{\frac{\boldsymbol{N}}{\mathbf{2}}}})=\boldsymbol{log}(\frac{\boldsymbol{a}}{\boldsymbol{c}})=\boldsymbol{log}(\frac{\sum\boldsymbol{a}_{\boldsymbol{i}}}{\sum\boldsymbol{c}_{\boldsymbol{i}}})=\boldsymbol{log}(\frac{\sum{(\boldsymbol{c}}_{\boldsymbol{i}}*\boldsymbol{e}^{\boldsymbol{\theta}_{\boldsymbol{i}}})}{\sum\boldsymbol{c}_{\boldsymbol{i}}})=\boldsymbol{log}(\frac{\sum{(\boldsymbol{k}}_{\boldsymbol{i}}*\frac{\boldsymbol{N}}{\mathbf{2}}*\boldsymbol{e}^{\boldsymbol{\theta}_{\boldsymbol{i}}})}{\sum{(\boldsymbol{k}}_{\boldsymbol{i}}*\frac{\boldsymbol{N}}{\mathbf{2}})})=\boldsymbol{log}(\frac{\sum{(\boldsymbol{k}}_{\boldsymbol{i}}*\boldsymbol{e}^{\boldsymbol{\theta}_{\boldsymbol{i}}})}{\sum\boldsymbol{k}_{\boldsymbol{i}}})$$

And $\boldsymbol{k}_{\boldsymbol{sum}}$:

$$\boldsymbol{k}_{\boldsymbol{sum}}=\frac{\boldsymbol{c}}{\frac{\boldsymbol{N}}{\mathbf{2}}}=\frac{\sum\boldsymbol{c}_{\boldsymbol{i}}}{\frac{\boldsymbol{N}}{\mathbf{2}}}=\sum\boldsymbol{k}_{\boldsymbol{i}}$$

# Supplementary Table 1. PRISMA checklist.

**PRISMA 2020 Main Checklist**

| **Topic** | **No.** | **Item** | **Location where item is reported** |
| --- | --- | --- | --- |
| **TITLE** |  |  |  |
| **Title** | 1 | Identify the report as a systematic review. | Page 1 |
| **ABSTRACT** |  |  |  |
| **Abstract** | 2 | See the PRISMA 2020 for Abstracts checklist |  |
| **INTRODUCTION** |  |  |  |
| **Rationale** | 3 | Describe the rationale for the review in the context of existing knowledge. | Section 1 |
| **Objectives** | 4 | Provide an explicit statement of the objective(s) or question(s) the review addresses. | Section 1 |
| **METHODS** |  |  |  |
| **Eligibility criteria** | 5 | Specify the inclusion and exclusion criteria for the review and how studies were grouped for the syntheses. | Section 2.1 |
| **Information sources** | 6 | Specify all databases, registers, websites, organisations, reference lists and other sources searched or consulted to identify studies. Specify the date when each source was last searched or consulted. | Section 2.1 |
| **Search strategy** | 7 | Present the full search strategies for all databases, registers and websites, including any filters and limits used. | Supplementary Material, File 1 |
| **Selection process** | 8 | Specify the methods used to decide whether a study met the inclusion criteria of the review, including how many reviewers screened each record and each report retrieved, whether they worked independently, and if applicable, details of automation tools used in the process. | Section 2.1 |
| **Data collection process** | 9 | Specify the methods used to collect data from reports, including how many reviewers collected data from each report, whether they worked independently, any processes for obtaining or confirming data from study investigators, and if applicable, details of automation tools used in the process. | Section 2.1 |
| **Data items** | 10a | List and define all outcomes for which data were sought. Specify whether all results that were compatible with each outcome domain in each study were sought (e.g. for all measures, time points, analyses), and if not, the methods used to decide which results to collect. | Section 2.2 |
|  | 10b | List and define all other variables for which data were sought (e.g. participant and intervention characteristics, funding sources). Describe any assumptions made about any missing or unclear information. | Section 2.2 |
| **Study risk of bias assessment** | 11 | Specify the methods used to assess risk of bias in the included studies, including details of the tool(s) used, how many reviewers assessed each study and whether they worked independently, and if applicable, details of automation tools used in the process. | Section 2.3, 3.1 |
| **Effect measures** | 12 | Specify for each outcome the effect measure(s) (e.g. risk ratio, mean difference) used in the synthesis or presentation of results. | Section 2.3 |
| **Synthesis methods** | 13a | Describe the processes used to decide which studies were eligible for each synthesis (e.g. tabulating the study intervention characteristics and comparing against the planned groups for each synthesis (item 5)). | N/A |
|  | 13b | Describe any methods required to prepare the data for presentation or synthesis, such as handling of missing summary statistics, or data conversions. | Section 2.2 |
|  | 13c | Describe any methods used to tabulate or visually display results of individual studies and syntheses. | Section 2.3 |
|  | 13d | Describe any methods used to synthesize results and provide a rationale for the choice(s). If meta-analysis was performed, describe the model(s), method(s) to identify the presence and extent of statistical heterogeneity, and software package(s) used. | Section 2.3, Section 2.4 |
|  | 13e | Describe any methods used to explore possible causes of heterogeneity among study results (e.g. subgroup analysis, meta-regression). | Section 2.3 |
|  | 13f | Describe any sensitivity analyses conducted to assess robustness of the synthesized results. | Section 3.1 |
| **Reporting bias assessment** | 14 | Describe any methods used to assess risk of bias due to missing results in a synthesis (arising from reporting biases). | N/A |
| **Certainty assessment** | 15 | Describe any methods used to assess certainty (or confidence) in the body of evidence for an outcome. | N/A |
| **RESULTS** |  |  |  |
| **Study selection** | 16a | Describe the results of the search and selection process, from the number of records identified in the search to the number of studies included in the review, ideally using a flow diagram. | Figure 1 |
|  | 16b | Cite studies that might appear to meet the inclusion criteria, but which were excluded, and explain why they were excluded. | N/A |
| **Study characteristics** | 17 | Cite each included study and present its characteristics. | Table S2 |
| **Risk of bias in studies** | 18 | Present assessments of risk of bias for each included study. | Figure S4 |
| **Results of individual studies** | 19 | For all outcomes, present, for each study: (a) summary statistics for each group (where appropriate) and (b) an effect estimate and its precision (e.g. confidence/credible interval), ideally using structured tables or plots. | Figure 2, Figure S6 |
| **Results of syntheses** | 20a | For each synthesis, briefly summarise the characteristics and risk of bias among contributing studies. | Table S3, Figure S4 |
|  | 20b | Present results of all statistical syntheses conducted. If meta-analysis was done, present for each the summary estimate and its precision (e.g. confidence/credible interval) and measures of statistical heterogeneity. If comparing groups, describe the direction of the effect. | Figure 2, Figure S6 |
|  | 20c | Present results of all investigations of possible causes of heterogeneity among study results. | Table 1 |
|  | 20d | Present results of all sensitivity analyses conducted to assess the robustness of the synthesized results. | Figure S5 |
| **Reporting biases** | 21 | Present assessments of risk of bias due to missing results (arising from reporting biases) for each synthesis assessed. | N/A |
| **Certainty of evidence** | 22 | Present assessments of certainty (or confidence) in the body of evidence for each outcome assessed. | N/A |
| **DISCUSSION** |  |  |  |
| **Discussion** | 23a | Provide a general interpretation of the results in the context of other evidence. | Section 4, Page 8-10 |
|  | 23b | Discuss any limitations of the evidence included in the review. | Section 4, Page 9 |
|  | 23c | Discuss any limitations of the review processes used. | Section 4, Page 9 |
|  | 23d | Discuss implications of the results for practice, policy, and future research. | Section 4, Page 10 |
| **OTHER INFORMATION** |  |  |  |
| **Registration and protocol** | 24a | Provide registration information for the review, including register name and registration number, or state that the review was not registered. | N/A |
|  | 24b | Indicate where the review protocol can be accessed, or state that a protocol was not prepared. | N/A |
|  | 24c | Describe and explain any amendments to information provided at registration or in the protocol. | N/A |
| **Support** | 25 | Describe sources of financial or non-financial support for the review, and the role of the funders or sponsors in the review. | Sections Funding, Acknowledgements |
| **Competing interests** | 26 | Declare any competing interests of review authors. | Section Conflict of interest |
| **Availability of data, code and other materials** | 27 | Report which of the following are publicly available and where they can be found: template data collection forms; data extracted from included studies; data used for all analyses; analytic code; any other materials used in the review. | Section Data availability statement |

# PRIMSA Abstract Checklist

| **Topic** | **No.** | **Item** | **Reported?** |
| --- | --- | --- | --- |
| **TITLE** |  |  |  |
| **Title** | 1 | Identify the report as a systematic review. | Yes |
| **BACKGROUND** |  |  |  |
| **Objectives** | 2 | Provide an explicit statement of the main objective(s) or question(s) the review addresses. | Yes |
| **METHODS** |  |  |  |
| **Eligibility criteria** | 3 | Specify the inclusion and exclusion criteria for the review. | Yes |
| **Information sources** | 4 | Specify the information sources (e.g. databases, registers) used to identify studies and the date when each was last searched. | Yes |
| **Risk of bias** | 5 | Specify the methods used to assess risk of bias in the included studies. | No |
| **Synthesis of results** | 6 | Specify the methods used to present and synthesize results. | Yes |
| **RESULTS** |  |  |  |
| **Included studies** | 7 | Give the total number of included studies and participants and summarise relevant characteristics of studies. | Yes |
| **Synthesis of results** | 8 | Present results for main outcomes, preferably indicating the number of included studies and participants for each. If meta-analysis was done, report the summary estimate and confidence/credible interval. If comparing groups, indicate the direction of the effect (i.e. which group is favoured). | No |
| **DISCUSSION** |  |  |  |
| **Limitations of evidence** | 9 | Provide a brief summary of the limitations of the evidence included in the review (e.g. study risk of bias, inconsistency and imprecision). | No |
| **Interpretation** | 10 | Provide a general interpretation of the results and important implications. | Yes |
| **OTHER** |  |  |  |
| **Funding** | 11 | Specify the primary source of funding for the review. | No |
| **Registration** | 12 | Provide the register name and registration number. | No |

# Supplementary Table 2. Summary of selected trials.

| **Study ID** | **Therapy (C/A)** | **Number of patients (C/A)** | **MACE composition** | **Duration of follow-up, y.** | **Prevention type** | **SRD patients** | **Age, y.** | **BMI, kg/m2** | **Number of men, %** | **Number of smokers, %** | **Number of hypertensive patients, %** | **LDLc, mg/dL** | **HDLc, mg/dL** | **Triglycerides, mg/dL** | **Remnant Ch, mg/dL** | **ΔLDLc, mg/dL** | **ΔHDLc, mg/dL** | **ΔTriglycerides, mg/dL** | **ΔRemnant Ch, mg/dL** |
| --- | --- | --- | --- | --- | --- | --- | --- | --- | --- | --- | --- | --- | --- | --- | --- | --- | --- | --- | --- |
| 1. AFCAPS/TexCAPS(Downs et al., 1998) | placebo/lovastatin (20-40 mg) | 3301/3304 | MI, UA, CVM | 5.2 | I* | 0 | 58 | 27 | 85 | 12.5 | 22 | 144.5 | 37.8 | 160.3 | 29.4 | -40 | 1.5 | -21.4 | -21.8 |
| 2. ALLHAT-LLT(The ALLHAT Officers and Coordinators for the ALLHAT Collaborative Research Group, 2002) | soc/pravastatin (40 mg) | 5185/5170 | TM | 4.8 | I, II | 0 | 66.3 | 29.9 | 51 | 23.2 | 100 | 129.1 | 47.1 | 146.8 | 29.2 | -17.3 | 3.4 | 0.2 | -5 |
| 3. ALLIANCE(Koren and Hunninghake, 2004) | soc/atorvastatin (10-80 mg) | 1225/1217 | CA*, CVM, nfMI, CR, UA | 4.3 | II | 0 | 61.2 | 29.5 | 82.2 | 19.5 | 59.5 | 124.5 | 41.5 | 187 | 36.5 | -16 | 0 | -12 | -4 |
| 4. ASCOT-LLA(Sever et al., 2003) | placebo/atorvastatin (10 mg) | 5137/5168 | nfMI, CM | 3.3 | I | 1 | 63.2 | 28.6 | 81.2 | 32.7 | 100 | 120.5 | 50.5 | 136.6 | 26 | -36.7 | 0.8 | -18.1 | -2.7 |
| 5. AtoZ(de Lemos et al., 2004)  (NCT00251576) | placebo+simstatin (20 mg)/  simvastatin (40-80 mg) | 2232/2265 | CVM, nfMI, ST, ACS* | 2 | II | 0 | 61 | 27.5 | 75.5 | 41 | 50 | 92.5 | 41.5 | 135.5 | 32 | -16 | 0 | -12 | -4 |
| 6. AURORA(Fellström et al., 2009)  (NCT00240331) | placebo/rosuvastatin (10 mg) | 1384/1389 | CVM, nfMI, nfST | 3.8 | I, II | 1 | 64.2 | 25.4 | 62.1 | 15.4 | 61 | 88.3 | 45.4 | 149.5 | 29.4 | -41 | 0.9 | -26.8 | -5.9 |
| 7. AVERT(Bertram et al., 1999) | soc+angioplasty/  atorvastatin (80 mg) | 177/164 | CVM, CA, nfMI, ST, CR, UA | 1.5 | NA* | 0 | 58.5 | NA | 84 | 22.5 | 45.5 | 121.8 | 44.3 | 157.9 | 31.8 | -39 | -1 | -33.3 | -6.8 |
| 8. CARDS(Colhoun et al., 2004)  (NCT00327418) | placebo/atorvastatin (10 mg) | 1410/1428 | MI, UA, CM, CA, CR, ST | 3.9 | I | 0 | 61.6 | 28.8 | 68 | 22.5 | 84 | 109.1 | 51.2 | 152.2 | 34 | -39.8 | 2.3 | -28.3 | -7.8 |
| 9. CARE (≥65 years)(Lewis, 1998) | placebo/pravastatin (40 mg) | 643/640 | CM, nfMI, CR, ST | 5 | II | 0 | 69 | 27 | 81.5 | 11.5 | 48 | 124.7 | 40.5 | 142.1 | 28.9 | -40 | 1.6 | -17.6 | -3.2 |
| 10. CARE (<65 years)(Lewis, 1998) | placebo/pravastatin (40 mg) | 1435/1441 | CM, nfMI, CR, ST | 5 | II | 0 | 54 | 28 | 88 | 24.5 | 40.5 | 126.5 | 38.6 | 151.9 | 30.6 | -37.5 | 1.9 | -22.4 | -4.1 |
| 11. CORONA(Kjekshus et al., 2007)  (NCT00206310) | placebo/rosuvastatin (10 mg) | 2497/2514 | CVM, nfMI, nfST | 2.7 | II | 1 | 73 | 27 | 76 | 8.5 | 63 | 121.9 | 48.1 | 167.5 | 20 | -62.2 | 2.3 | -41.8 | -8 |
| 12. FOURIER(Sabatine et al., 2015)  (NCT01764633) | placebo/evolocumab (140 mg Q2W/420 mg Q4W) | 13780/13784 | CVM, MI, ST, UA, CR | 2.2 | II | 0 | 62.5 | 29.5 | 75.5 | 28.2 | 80.1 | 77 | 45.3 | 127.8 | 30.8 | -54 | 2.4 | -20.8 | -8 |
| 13. GISSI-HF(GISSI-HF investigators, 2008)  (NCT00336336) | placebo/rosuvastatin (10 mg) | 2289/2285 | TM/CVM* | 3.9 | II | 0 | 68 | 27.1 | 77.4 | 14.1 | 52.8 | 112.7 | NA | NA | NA | -30.2 | NA | NA | NA |
| 14. GREACE(Athyros et al., 2002) | soc/atorvastatin (10-80 mg) | 800/800 | TM, nfMI, UA, HF, CR, ST | 3 | II | 0 | 58.5 | 24 | 78.5 | 25 | 43 | 156.2 | 40 | 165.5 | 36.8 | -73 | 2 | -50 | -21 |
| 15. IDEAL(Pedersen et al., n.d.)  (NCT00159835) | simvastatin (20 mg)/atorvastatin (80 mg) | 4449/4439 | CM, nfMI, CA | 4.8 | II | 0 | 61.7 | 27.3 | 80.8 | 20.6 | 33 | 105.7 | 48.2 | 138.3 | 26.8 | -20 | -0.4 | -23.2 | -3.9 |
| 16. JUPITER(Ridker et al., 2008)  (NCT00239681) | placebo/rosuvastatin (20 mg) | 8901/8901 | CVM, MI, ST, UA, CR | 1.9 | I | 0 | 66 | 28.4 | 61.8 | 15.8 | 57.4 | 95 | 49.5 | 113.2 | 25.1 | -54 | 0 | -19 | -4.8 |
| 17. KLIS(The Kyushu Lipid Intervention Study Group, 2000) | soc/pravastatin (10-20 mg) | 1634/2219 | MI, CR, CVM, sudden death | 5 | I, II | 0 | 58 | 24 | 100 | 39.2 | 43 | 151 | NA | NA | NA | -20.2 | NA | NA | NA |
| 18. LIPID(Long-Term Intervention with Pravastatin in Ischaemic Disease (LIPID) Study Group, 1998) | placebo/pravastatin (40 mg) | 4502/4512 | CM | 6.1 | II | 0 | 62 | 27.5 | 83 | 9.5 | 41.5 | NA | NA | NA | NA | -38.7 | 1.9 | -17.7 | -3.5 |
| 19. LIPS(Serruys, 2002) | placebo/fluvastatin (80 mg) | 833/844 | CVM, nfMI | 3.9 | II | 0 | 60 | 26.6 | 83.9 | 26.6 | 38.6 | 122.7 | 41.6 | 148.8 | 29 | -35.4 | 0.2 | 0 | -1 |
| 20. MEGA(Nakamura et al., 2006)  (NCT00211705) | diet/diet+pravastatin (10-20 mg) | 3966/3866 | MI, cardiac sudden death, UA, CR | 5.3 | I | 0 | 58.3 | 23.8 | 31.5 | 20.5 | 42 | 140.4 | 59.7 | 123.6 | 27.1 | -19.3 | 3.1 | -6.2 | -4.3 |
| 21. HPS(Heart Protection Study Collaborative Group, 2002)  (ISRCTN48489393) | placebo/simvastatin (40 mg) | 10267/10269 | TM, CM, nfMI, ST, CR | 5 | II | 0 | 60 | 27.5 | 75.2 | 22.7 | 41 | NA | NA | NA | NA | -27.1 | 0.8 | -17.7 | -3.5 |
| 22. ODYSSEY OUTCOMES(Schwartz et al., 2018)  (NCT01663402) | placebo/alirocumab (75 mg Q2W) | 9462/9462 | CM, nfMI, iST, UA | 4 | II | 0 | 58 | 28.5 | 74.8 | 24.1 | 64.8 | 84.9 | 45.6 | 145 | 29 | -48.1 | 2.8 | -12 | -2.4 |
| 23. postCABG(Post Coronary Artery Bypass Graft Trial Investigators, 1997) | warfarin (1-4 mg)+lovastatin (2.5mg)/warfarin (1-4 mg)+lovastatin (40 mg) | 675/676 | CVM, nfMI, ST, CR | 4.3 | II | 0 | 61.5 | 27.5 | 92.3 | 11.3 | 60 | 135.5 | 41.3 | 163.2 | 24.5 | -38.8 | 0.6 | -11.2 | 6.1 |
| 24. PREVEND IT(Asselbergs et al., 2004) | placebo/pravastatin (40 mg) | 431/433 | CVM, nfMI, HF, ST, peripheral vascular disease | 3.8 | I | 0 | 51.3 | 26 | 65 | 40 | 51 | 146 | NA | NA | NA | -34.8 | -0.6 | -0.2 | -7.7 |
| 25. PROSPER(Shepherd et al., 2002) | placebo/pravastatin (40 mg) | 2913/2891 | CM, nfMI, ST | 3.2 | I, II | 0 | 75.3 | 26.8 | 48.3 | 26.8 | 61.9 | 124.4 | 51.9 | 125.9 | 24.1 | -32.9 | 2.7 | -15 | -3 |
| 26. PROVE IT-TIMI 22(Cannon et al., 2004)  (NCT00382460) | pravastatin (40 mg)/atorvastatin (80 mg) | 2063/2099 | TM, MI, UA, CR, ST | 2 | II | 0 | 58.2 | 27.5 | 78.1 | 36.8 | 50.2 | 92.2 | 39.9 | NA | NA | -33 | -0.7 | NA | NA |
| 27. REGRESS(Jukema et al., 1995) | placebo/pravastatin (40 mg) | 434/450 | MI, CM, CR, ST, TIA, TM | 2 | II | 0 | 55.5 | 26 | 100 | 28 | 28 | 157.1 | 36.7 | 149.1 | 29.2 | -44.5 | 3.1 | -19.5 | -3.9 |
| 28. SEARCH(Study of the Effectiveness of Additional Reductions in Cholesterol and Homocysteine (SEARCH) Collaborative Group, 2010)  (NCT00124072) | simvastatin (20 mg)/simvastatin (80 mg) | 6033/6031 | CM, MI, ST, CR | 6.7 | II | 0 | 64.2 | 27.5 | 83 | 12.3 | 42.1 | NA | NA | NA | NA | -13.2 | 0.8 | -15 | -3 |
| 29. SPARCL(Karam et al., 2008)  (NCT00147602) | placebo/atorvastatin (80 mg) | 2366/2365 | ST | 4.9 | I, II | 0 | 62.8 | 27.4 | 59.6 | 19.2 | 61.9 | 116.9 | 50.8 | 136 | 27.1 | -54.6 | 1.1 | -34.5 | -6.8 |
| 30. 4S(Pedersen et al., n.d.) | placebo/simvastatin (20-40 mg) | 2223/2221 | TM | 10 | II | 0 | 58.5 | 26 | 81.5 | 25.5 | 26 | 176.5 | 46.4 | 132.7 | 22.5 | -60.3 | 1.5 | -23 | -9 |
| 31. TNT(LaRosa et al., 2005)  (NCT03073863) | atorvastatin (10 mg)/atorvastatin (80 mg) | 5006/4995 | CM, nfMI, CA, ST | 4.9 | II | 0 | 61 | 28.5 | 81 | 13.4 | 53.7 | 94.1 | 47.1 | 150.1 | 29.8 | -20.4 | 0.6 | -25 | -6.2 |
| 32. WOSCOPS(Shepherd et al., 1995) | placebo/pravastatin (40 mg) | 3293/3302 | CM, nfMI | 10 | I | 0 | 55.2 | 26 | 100 | 44 | 15.7 | 178.9 | NA | NA | NA | -51 | NA | NA | NA |
| 33. ACAPS(Furberg et al., 1994)  (NCT00000469) | placebo/lovastatin (20-40 mg) | 459/460 | MI, CM, ST | 3 | I | 0 | 61.7 | 25.9 | 51.5 | 11.9 | 28.8 | 144.3 | 51.7 | 140.2 | 27.4 | -33.7 | 2.9 | -16.3 | -3.5 |
| 34. SCAT(Teo et al., 2000) | placebo/simvastatin (10-40 mg) | 230/230 | - | 5 | I, II | 0 | 61.9 | NA | 89 | 15 | 36 | 120.6 | 39.1 | 158.4 | 31.5 | -44.9 | 1.5 | -33.6 | -5.4 |
| 35. ALERT(Holdaas et al., 2003) | placebo/fluvastatin (40-80 mg) | 1052/1050 | CVM, nfMI, CR | 5.4 | I | 1 | 49.8 | 25.8 | 66 | 18.5 | 74.9 | 143 | NA | NA | NA | -43.7 | NA | NA | NA |
| 36. J-STARS(Hosomi et al., 2015)  (NCT00221104) | soc/pravastatin (10 mg) | 785/793 | ST, TIA | 4.9 | II | 0 | 66.2 | 23.7 | 68.9 | 53.6 | 76 | 119.8 | 54.2 | 135.6 | 26.4 | -16 | 4.3 | -11.8 | -6.8 |
| 37. CAIUS(Mercuri et al., 1996) | placebo/pravastatin (40 mg) | 154/151 | MI, UA | 3 | I | 0 | 55 | 24.7 | 53 | 24 | NA | 172.1 | 53.4 | 137.6 | 27.9 | -43.3 | 2.3 | 0 | -5 |
| 38. 30983166(Kitas et al., 2019) | soc/atorvastatin (40 mg) | 1498/1504 | CVM, MI, ST, TIA, CR | 2.5 | I | 0 | 61.1 | 26.4 | 25.8 | 18 | 21.8 | 112 | 56 | 108.8 | 22.3 | -29.8 | 2.7 | -10.6 | -5 |
| 39. 31987664(Moroi et al., 2020) | atorvastatin (10 mg)/pitavastatin (2 mg) | 310/312 | CVM, sudden death, nfMI, nfST, TIA, HF | 4.6 | I, II | 0 | 65.3 | 24.6 | 54 | NA | 74.4 | 120 | 54.9 | 152.2 | 29.3 | 1.4 | -2.9 | 10.7 | 1.5 |
| 40. 16034009(Wanner et al., 2005) | placebo/atorvastatin (20 mg) | 636/619 | CVM, nfMI, ST | 4 | I, II | 1 | 65.7 | 27.5 | 54 | 8.6 | 100 | 109.5 | NA | NA | NA | -44 | NA | NA | NA |
| 41. ORION-9(Raal et al., 2020)  (NCT03397121) | placebo/inclisiran (300 mg) on days 1, 90, 270, and 450 | 240/242 | MI, ST, CVM | 1.5 | NA | 0 | 56 | NA | 47 | 11.6 | 42 | 140.8 | 53 | 116 | 26.3 | -68.9 | 1.4 | -12.5 | -8.3 |
| 42. ORION-10(Ray et al., 2020)  (NCT03399370) | placebo/inclisiran (284 mg) on days 1, 90 and Q24W | 780/781 | MI, ST, CVM | 1.5 | NA | 0 | 66 | NA | 69.3 | 15 | 90.6 | 90.1 | 47.4 | 123.8 | 28 | -54.1 | 2.4 | -21.2 | -10.1 |
| 43. ORION-11(Ray et al., 2020)  (NCT03400800) | placebo/inclisiran (284 mg) on days 1, 90 and Q24W | 807/811 | MI, ST, CVM | 1.5 | NA | 0 | 64.8 | NA | 71.7 | 18 | 80.5 | 93 | 51.3 | 129.3 | 28.8 | -51.9 | 3 | -9.5 | -6.9 |
| 44. PLAC-I(Pitt et al., 1995) | placebo/pravastatin (40 mg) | 202/206 | MI, CM, TM, nfMI, nfST, TM, CR | 3 | I, II | 0 | 57 | NA | 38 | 8 | 22.3 | 149.2 | 42.2 | 166.6 | 26.4 | -47.6 | 2.1 | -28.2 | -3 |
| 45. SATURN(Nicholls et al., 2011)  (NCT000620542) | atorvastatin (40-80 mg)/rosuvastatin (20-40 mg) | 689/691 | CVM, nfMI, nfST, UA, CR | 2 | I, II | 0 | 57.6 | 29 | 73.6 | 32.3 | 70.3 | 93.2 | 47.2 | 122 | 27.3 | -7.7 | 1.2 | 12 | 1.4 |
| 46. LISK(Sasaki et al., 2002) | soc/pravastatin (10-20 mg) | 498/587 | UA, MI, ST, TM | 5 | I | 0 | 55.5 | 23.5 | 37.2 | 18.5 | 37.7 | 175.6 | 55.1 | 178.8 | 17 | -21.3 | 7 | 9.8 | 8.6 |
| 47. PHYLLIS(Moneta, 2006) | hydrochlorothiazide/ hydrochlorothiazide + pravastatin  fosinopril/fosinopril+pravastatin | 127/126  127/128 | - | 2.6 | I | 0 | 58.4 | 25.8 | 40.2 | 16.2 | 100 | 169.3 | 53.1 | 140.1 | 27.9 | -36.4 | 1.3 | -11.4 | -2.3 |
| 48. METEOR(Crouse et al., 2007)  (NCT00225589) | placebo/rosuvastatin (40 mg) | 282/702 | UA, ACS, MI | 2 | I, II | 0 | 57 | 27.3 | 59.8 | 3.9 | 19.9 | 134.8 | 50.5 | 124.2 | 24.8 | -75 | 2 | -33 | -5 |
| 49. COMBO II(Cannon et al., 2015)  (NCT01644188) | ezetimibe (10 mg)/alirocumab (75-150 mg Q2W) | 241/479 | - | 2 | I | 0 | 61.5 | 30.1 | 73.6 | NA | NA | 86.8 | 47.5 | 128.3 | 31 | -32.7 | 3.8 | -1.4 | 1.6 |
| 50. HOPE-3(Yusuf et al., 2016)  (NCT00468923) | candesartan (16 mg) + hydrochlorothiazide (12.5 mg)/candesartan (16 mg) + hydrochlorothiazide (12.5 mg) + rosuvastatin (10 mg)  placebo/rosuvastatin (10 mg) | 3176/3180  3168/3181 | CVM, nfMI, nfST/ CVM, nfMI, nfST, HF, CA, CR | 5.6 | I | 0 | 65.7 | 27.1 | 53.8 | 27.8 | 37.9 | 118.4 | NA | NA | NA | -27.8 | NA | NA | NA |
| 51. REAL-CAD(Taguchi et al., 2018)  (NCT01042730) | pitavastatin (1 mg)/pitavastatin (4 mg) | 6214/6199 | CVM, nfMI, non-fatal iST, UA | 3 | II | 1 | 68 | 24.6 | 82.6 | 16.4 | 75.7 | 85.8 | 51.4 | 121 | 25.9 | -14 | 0.6 | -7 | -1.8 |
| 52. 31752850(Jeong et al., 2019)  (NCT02545231) | pitavastatin (1 mg)/pitavastatin (4 mg) | 251/251 | - | 3 | I, II | 0 | 63 | 24.5 | 72.1 | 25.7 | 57 | 109.9 | 44.8 | 146.8 | 10.6 | -23.4 | -1.3 | -18.1 | 13.8 |
| 53. GLAGOV(Nicholls et al., 2016)  (NCT01813422) | statins/statins+evolocumab (420 mg Q76W) | 484/484 | TM, CVM, MI, UA, CR, ST, TIA, HF | 1.5 | NA | 0 | 59.8 | 29.4 | 72.2 | 24.4 | 83 | 78.7 | 47.1 | 120 | 26.8 | -56.6 | 2.5 | -19 | -6.3 |
| 54. ASPEN(Knopp et al., 2006) | placebo/atorvastatin | 1199/1211 | CVM, nfMI, nfST, CR, CA, UA | 4 | I, II | 0 | 61 | 28.9 | 66.5 | 12.5 | 55 | 104.6 | 47.2 | 148.2 | 31.9 | -33 | 1.1 | -20.2 | -3.6 |

CA – cardiac arrest

ACS - acute coronary syndrome

TM/CVM - co-primary endpoints

NA – not available

Prevention type: I – primary, II – secondary, I, II - both

**References**

Asselbergs, F. W., Diercks, G. F. H., Hillege, H. L., van Boven, A. J., Janssen, W. M. T., Voors, A. A., et al. (2004). Effects of Fosinopril and Pravastatin on Cardiovascular Events in Subjects With Microalbuminuria. *Circulation* 110, 2809–2816. doi: 10.1161/01.CIR.0000146378.65439.7A.

Athyros, V. G., Papageorgiou, A. A., Mercouris, B. R., Athyrou, V. V., Symeonidis, A. N., Basayannis, E. O., et al. (2002). Treatment with Atorvastatin to the National Cholesterol Educational Program Goal Versus “Usual” Care in Secondary Coronary Heart Disease Prevention. *Current Medical Research and Opinion* 18, 220–228. doi: 10.1185/030079902125000787.

Bertram, P., David, W., and Virgil, B. W. (1999). Aggressive Lipid-Lowering Therapy Compared with Angioplasty in Stable Coronary Artery Disease. *The New England Journal of Medicine* 342, 70–76. doi: 10.1056/NEJM199907083410202.

Cannon, C. P., Cariou, B., Blom, D., McKenney, J. M., Lorenzato, C., Pordy, R., et al. (2015). Efficacy and safety of alirocumab in high cardiovascular risk patients with inadequately controlled hypercholesterolaemia on maximally tolerated doses of statins: the ODYSSEY COMBO II randomized controlled trial. *Eur Heart J* 36, 1186–1194. doi: 10.1093/eurheartj/ehv028.

Cannon, C. P., Rouleau, J. L., and Skene, A. M. (2004). Intensive versus Moderate Lipid Lowering with Statins after Acute Coronary Syndromes. *The New England Journal of Medicine* 350, 1495–1504.

Colhoun, H. M., Betteridge, D. J., Durrington, P. N., Hitman, G. A., Neil, H. A. W., Livingstone, S. J., et al. (2004). Primary prevention of cardiovascular disease with atorvastatin in type 2 diabetes in the Collaborative Atorvastatin Diabetes Study (CARDS): multicentre randomised placebo-controlled trial. *The Lancet* 364, 685–689. doi: 10.1016/S0140-6736(04)16895-5.

Crouse, J. R., Raichlen, J. S., Riley, W. A., Evans, G. W., Palmer, M. K., O’Leary, D. H., et al. (2007). Effect of Rosuvastatin on Progression of Carotid Intima-Media Thickness in Low-Risk Individuals With Subclinical Atherosclerosis: The METEOR Trial. *JAMA* 297, 1344. doi: 10.1001/jama.297.12.1344.

de Lemos, J.A., Blazing, M.A., Wiviott, S.D., Lewis, E.F., Fox, K.A., White, H.D., et al. (2004) Early Intensive vs a Delayed Conservative Simvastatin Strategy in Patients With Acute Coronary Syndromes Phase Z of the A to Z Trial. *JAMA* 292, 1307–1316. doi: 10.1001/jama.292.11.1307.

Downs, J. R., Clearfield, M., Weis, S., Whitney, E., Shapiro, D. R., Beere, P. A., et al. (1998). Primary Prevention of Acute Coronary Events With Lovastatin in Men and Women With Average Cholesterol Levels: Results of AFCAPS/TexCAPS. *JAMA* 279, 1615. doi: 10.1001/jama.279.20.1615.

Fellström, B. C., Jardine, A. G., Schmieder, R. E., Holdaas, H., Bannister, K., Beutler, J., et al. (2009). Rosuvastatin and Cardiovascular Events in Patients Undergoing Hemodialysis. *N Engl J Med* 360, 1395–1407. doi: 10.1056/NEJMoa0810177.

Furberg, C. D., Adams, H. P., Applegate, W. B., Byington, R. P., Espeland, M. A., Hartwell, T., et al. (1994). Effect of Lovastatin on Early Carotid Atherosclerosis and Cardiovascular Events. Asymptomatic Carotid Artery Progression Study (ACAPS) Research Group. *Circulation* 90, 1679–1687. doi: 10.1161/01.cir.90.4.1679.

GISSI-HF investigators (2008). Effect of rosuvastatin in patients with chronic heart failure (the GISSI-HF trial): a randomised, double-blind, placebo-controlled trial. *The Lancet* 372, 1231–1239. doi: 10.1016/S0140-6736(08)61240-4.

Heart Protection Study Collaborative Group. MRC/BHF Heart Protection Study of cholesterol lowering with simvastatin in 20 536 high-risk individuals: a randomised placebocontrolled trial (2002). *The Lancet* 360, 7–22. doi: 10.1016/S0140-6736(02)09327-3.

Holdaas, H., Fellström, B., Jardine, A. G., Holme, I., Nyberg, G., Fauchald, P., et al. (2003). Effect of fluvastatin on cardiac outcomes in renal transplant recipients: a multicentre, randomised, placebo-controlled trial. *The Lancet* 361, 2024–2031. doi: 10.1016/S0140-6736(03)13638-0.

Hosomi, N., Nagai, Y., Kohriyama, T., Ohtsuki, T., Aoki, S., Nezu, T., et al. (2015). The Japan Statin Treatment Against Recurrent Stroke (J-STARS): A Multicenter, Randomized, Open-label, Parallel-group Study. *EBioMedicine* 2, 1071–1078. doi: 10.1016/j.ebiom.2015.08.006.

Jeong, H. S., Hong, S. J., Son, S., An, H., Kook, H., Joo, H. J., et al. (2019). Incidence of new-onset diabetes with 1 mg versus 4 mg pitavastatin in patients at high risk of developing diabetes during a 3-year follow-up. *Cardiovasc Diabetol* 18, 162. doi: 10.1186/s12933-019-0969-z.

Jukema, J. W., Bruschke, A. V. G., van Boven, A. J., Reiber, J. H. C., Bal, E. T., Zwinderman, A. H., et al. (1995). Effects of Lipid Lowering by Pravastatin on Progression and Regression of Coronary Artery Disease in Symptomatic Men With Normal to Moderately Elevated Serum Cholesterol Levels: The Regression Growth Evaluation Statin Study (REGRESS). *Circulation* 91, 2528–2540. doi: 10.1161/01.CIR.91.10.2528.

Karam, J. G., Loney-Hutchinson, L., and McFarlane, S. I. (2008). High-Dose Atorvastatin After Stroke or Transient Ischemic Attack: The Stroke Prevention by Aggressive Reduction in Cholesterol Levels (SPARCL) Investigators. *J CardioMetab Syndrome* 3, 68–69. doi: 10.1111/j.1559-4572.2008.07967.x.

Kitas, G. D., Nightingale, P., Armitage, J., Sattar, N., Belch, J. J. F., Symmons, D. P. M., et al. (2019). A Multicenter, Randomized, Placebo‐Controlled Trial of Atorvastatin for the Primary Prevention of Cardiovascular Events in Patients With Rheumatoid Arthritis. *Arthritis Rheumatol* 71, 1437–1449. doi: 10.1002/art.40892.

Kjekshus, J., Apetrei, E., Barrios, V., Böhm, M., Cleland, J. G. F., Cornel, J. H., et al. (2007). Rosuvastatin in Older Patients with Systolic Heart Failure. *N Engl J Med* 357, 2248–2261. doi: 10.1056/NEJMoa0706201.

Knopp, R. H., d’Emden, M., Smilde, J. G., Pocock, S. J., and on behalf of the ASPEN Study Group (2006). Efficacy and Safety of Atorvastatin in the Prevention of Cardiovascular End Points in Subjects With Type 2 Diabetes. *Diabetes Care* 29, 1478–1485. doi: 10.2337/dc05-2415.

Koren, M. J., and Hunninghake, D. B. (2004). Clinical outcomes in managed-care patients with coronary heart disease treated aggressively in lipid-lowering disease management clinics. *Journal of the American College of Cardiology* 44, 1772–1779. doi: 10.1016/j.jacc.2004.07.053.

LaRosa, J. C., Waters, D. D., Fruchart, J.-C., Greten, H., and Wenger, N. K. (2005). Intensive Lipid Lowering with Atorvastatin in Patients with Stable Coronary Disease. *The New England Journal of Medicine* 352, 1425–1435. doi: 10.1056/NEJMoa050461.

Lewis, S. J. (1998). Effect of Pravastatin on Cardiovascular Events in Older Patients with Myocardial Infarction and Cholesterol Levels in the Average Range: Results of the Cholesterol and Recurrent Events (CARE) Trial. *Ann Intern Med* 129, 681. doi: 10.7326/0003-4819-129-9-199811010-00002.

Long-Term Intervention with Pravastatin in Ischaemic Disease (LIPID) Study Group. Prevention of Cardiovascular Events and Death with Pravastatin in Patients with Coronary Heart Disease and a Broad Range of Initial Cholesterol Levels (1998). *The New England Journal of Medicine* 339, 1349–1357. doi: 10.1056/NEJM199811053391902.

Mercuri, M., Bond, M. G., Sirtori, C. R., Veglia, F., Crepaldi, G., Saverio Feruglio, F., et al. (1996). Pravastatin reduces carotid intima-media thickness progression in an asymptomatic hypercholesterolemic Mediterranean population: The Carotid Atherosclerosis Italian Ultrasound Study. *The American Journal of Medicine* 101, 627–634. doi: 10.1016/S0002-9343(96)00333-6.

Moneta, G. L. (2006). Different Effects of Antihypertensive Regimens Based on Fosinopril or Hydrochlorothiazide With or Without Lipid Lowering by Pravastatin on Progression of Asymptomatic Carotid Atherosclerosis: Principal Results of PHYLLIS—A Randomized Double-blind Trial. *Yearbook of Vascular Surgery* 2006, 287–288. doi: 10.1016/S0749-4041(08)70220-3.

Moroi, M., Nagayama, D., Hara, F., Saiki, A., Shimizu, K., Takahashi, M., et al. (2020). Outcome of pitavastatin versus atorvastatin therapy in patients with hypercholesterolemia at high risk for atherosclerotic cardiovascular disease. *International Journal of Cardiology* 305, 139–146. doi: 10.1016/j.ijcard.2020.01.006.

Nakamura, H., Arakawa, K., Itakura, H., Kitabatake, A., Goto, Y., Toyota, T., et al. (2006). Primary prevention of cardiovascular disease with pravastatin in Japan (MEGA Study): a prospective randomised controlled trial. *The Lancet* 368, 1155–1163. doi: 10.1016/S0140-6736(06)69472-5.

Nicholls, S. J., Ballantyne, C. M., Barter, P. J., Chapman, M. J., Erbel, R. M., Libby, P., et al. (2011). Effect of Two Intensive Statin Regimens on Progression of Coronary Disease. *N Engl J Med* 365, 2078–2087. doi: 10.1056/NEJMoa1110874.

Nicholls, S. J., Puri, R., Anderson, T., Ballantyne, C. M., Cho, L., Kastelein, J. J. P., et al. (2016). Effect of Evolocumab on Progression of Coronary Disease in Statin-Treated Patients: The GLAGOV Randomized Clinical Trial. *JAMA* 316, 2373. doi: 10.1001/jama.2016.16951.

Pedersen, T. R., Faergeman, O., Kastelein, J. J. P., Olsson, A. G., Tikkanen, M. J., Holme, I., et al. (2005). High-Dose Atorvastatin vs Usual-Dose Simvastatin for Secondary Prevention After Myocardial Infarction. *JAMA* 294, 2437–2445. doi: 10.1001/jama.294.19.2437.

Pedersen, T. R., Olsson, A. G., Wedel, H., Berg, K., Wilhelmsen, L., Haghfelt, T., et al. (1998). Lipoprotein Changes and Reduction in the Incidence of Major Coronary Heart Disease Events in the Scandinavian Simvastatin Survival Study (4S). *Circulation* 97, 1453–1460. doi: 10.1161/01.cir.97.15.1453.

Pitt, B., Mancini, G. B. J., Ellis, S. G., Rosman, H. S., Park, J.-S., and Mcgovern, M. E. (1995). Pravastatin limitation of atherosclerosis in the coronary arteries (PLAC I): Reduction in atherosclerosis progression and clinical events. *Journal of the American College of Cardiology* 26, 1133–1139. doi: 10.1016/0735-1097(95)00301-0.

Post Coronary Artery Bypass Graft Trial Investigators. The Effect of Aggressive Lowering of Low-Density Lipoprotein Cholesterol Levels and Low-Dose Anticoagulation on Obstructive Changes in Saphenous-Vein Coronary-Artery Bypass Grafts (1997). *The New England Journal of Medicine* 336, 153–162. doi: 10.1056/NEJM199701163360301.

Raal, F. J., Kallend, D., Ray, K. K., Turner, T., Koenig, W., Wright, R. S., et al. (2020). Inclisiran for the Treatment of Heterozygous Familial Hypercholesterolemia. *N Engl J Med* 382, 1520–1530. doi: 10.1056/NEJMoa1913805.

Ray, K. K., Wright, R. S., Kallend, D., Koenig, W., Leiter, L. A., Raal, F. J., et al. (2020). Two Phase 3 Trials of Inclisiran in Patients with Elevated LDL Cholesterol. *N Engl J Med* 382, 1507–1519. doi: 10.1056/NEJMoa1912387.

Ridker, P. M., Danielson, E., Fonseca, F. A. H., Genest, J., Gotto, A. M., Kastelein, J. J. P., et al. (2008). Rosuvastatin to Prevent Vascular Events in Men and Women with Elevated C-Reactive Protein. *N Engl J Med* 359, 2195–2207. doi: 10.1056/NEJMoa0807646.

Sabatine, M. S., Giugliano, R. P., Wiviott, S. D., Raal, F. J., Blom, D. J., Robinson, J., et al. (2015). Efficacy and Safety of Evolocumab in Reducing Lipids and Cardiovascular Events. *N Engl J Med* 372, 1500–1509. doi: 10.1056/NEJMoa1500858.

Sasaki, S., Nakagawa, M., Nakata, T., Azuma, A., Sawada, S., Takeda, K., et al. (2002). Effects of Pravastatin on Exercise Electrocardiography Test Performance and Cardiovascular Mortality and Morbidity in Patients With Hypercholesterolemia: Lipid Intervention Study in Kyoto. *Circ J* 66, 47–52. doi: 10.1253/circj.66.47.

Schwartz, G. G., Steg, P. G., Szarek, M., Bhatt, D. L., Bittner, V. A., Diaz, R., et al. (2018). Alirocumab and Cardiovascular Outcomes after Acute Coronary Syndrome. *N Engl J Med* 379, 2097–2107. doi: 10.1056/NEJMoa1801174.

Serruys, P. W. J. C. (2002). Fluvastatin for Prevention of Cardiac Events Following Successful First Percutaneous Coronary InterventionA Randomized Controlled Trial. *JAMA* 287, 3215. doi: 10.1001/jama.287.24.3215.

Sever, P. S., Dahlöf, B., Poulter, N. R., Wedel, H., Beevers, G., Caulfield, M., et al. (2003). Prevention of coronary and stroke events with atorvastatin in hypertensive patients who have average or lower-than-average cholesterol concentrations, in the Anglo-Scandinavian Cardiac Outcomes Trial—Lipid Lowering Arm (ASCOT-LLA): a multicentre randomised controlled trial. *The Lancet* 361.

Shepherd, J., Blauw, G. J., Murphy, M. B., Bollen, E. L., Buckley, B. M., Cobbe, S. M., et al. (2002). Pravastatin in elderly individuals at risk of vascular disease (PROSPER): a randomised controlled trial. *The Lancet* 360, 1623–1630. doi: 10.1016/S0140-6736(02)11600-X.

Shepherd, J., Cobbe, S. M., Ford, I., Isles, C. G., Lorimer, A. R., Macfarlane, P. W., et al. (1995). Prevention of Coronary Heart Disease with Pravastatin in Men with Hypercholesterolemia. *N Engl J Med* 333, 1301–1308. doi: 10.1056/NEJM199511163332001.

Study of the Effectiveness of Additional Reductions in Cholesterol and Homocysteine (SEARCH) Collaborative Group (2010). Intensive lowering of LDL cholesterol with 80 mg versus 20 mg simvastatin daily in 12 064 survivors of myocardial infarction: a double-blind randomised trial. *The Lancet* 376, 1658–1669. doi: 10.1016/S0140-6736(10)60310-8.

Taguchi, I., Iimuro, S., Iwata, H., Takashima, H., Abe, M., Amiya, E., et al. (2018). High-Dose Versus Low-Dose Pitavastatin in Japanese Patients With Stable Coronary Artery Disease (REAL-CAD): A Randomized Superiority Trial. *Circulation* 137, 1997–2009. doi: 10.1161/CIRCULATIONAHA.117.032615.

Teo, K. K., Burton, J. R., Buller, C. E., Plante, S., Catellier, D., Tymchak, W., et al. (2000). Long-Term Effects of Cholesterol Lowering and Angiotensin-Converting Enzyme Inhibition on Coronary Atherosclerosis: The Simvastatin/Enalapril Coronary Atherosclerosis Trial (SCAT). *Circulation* 102, 1748–1754. doi: 10.1161/01.CIR.102.15.1748.

The ALLHAT Officers and Coordinators for the ALLHAT Collaborative Research Group (2002). Major Outcomes in Moderately Hypercholesterolemic, Hypertensive Patients Randomized to Pravastatin vs Usual Care: The Antihypertensive and Lipid-Lowering Treatment to Prevent Heart Attack Trial (ALLHAT-LLT). *JAMA: The Journal of the American Medical Association* 288, 2998–3007. doi: 10.1001/jama.288.23.2998.

The Kyushu Lipid Intervention Study Group (2000). Pravastatin Use and Risk of Coronary Events and Cerebral Infarction in Japanese Men with Moderate Hypercholesterolemia: The Kyushu Lipid Intervention Study. *J Atheroscler Thromb* 7, 110–121. doi: 10.5551/jat1994.7.110.

Wanner, C., Olschewski, M., Mann, J. F. E., and Ritz, E. (2005). Atorvastatin in Patients with Type 2 Diabetes Mellitus Undergoing Hemodialysis. *The New England Journal of Medicine*.

Yusuf, S., Lonn, E., Pais, P., Bosch, J., López-Jaramillo, P., Zhu, J., et al. (2016). Blood-Pressure and Cholesterol Lowering in Persons without Cardiovascular Disease. *N Engl J Med* 374, 2032–2043. doi: 10.1056/NEJMoa1600177.

# Supplementary Table 3. Summary statistics of the covariates.

| **Covariate** | **N of trials** | **Mean** | **SD** | **Minimum value** | **Maximum value** | **Missingness, N** | **Missingness, %** |
| --- | --- | --- | --- | --- | --- | --- | --- |
| **Demographic characteristics** | | | | | | | |
| Age, years | 54 | 61.2 | 4.9 | 49.75 | 75.35 | 0 | 0 |
| Body mass index (BMI), kg/m^2^ | 48 | 26.89 | 1.75 | 23.5 | 30.15 | 6 | 11.11 |
| Males (%) | 54 | 69.73 | 17.23 | 25.8 | 100 | 0 | 0 |
| Systolic blood pressure (SBP), mmHg | 47 | 135.27 | 8.2 | 124.5 | 164.2 | 7 | 12.96 |
| Hypertension (%) | 52 | 54.71 | 22.61 | 15.7 | 100 | 2 | 3.7 |
| Diabetes (%) | 51 | 22.72 | 24.05 | 0 | 100 | 3 | 5.56 |
| Smokers (%) | 52 | 21.49 | 10.2 | 3.86 | 53.6 | 2 | 3.7 |
| **Therapy** | | | | | | | |
| PCSK9 inhibitors | 7 |  |  |  |  |  |  |
| Statins | 47 |  |  |  |  |  |  |
| **Inclusion of patients with renal disease** | | | | | | | |
| Yes | 6 |  |  |  |  |  |  |
| No | 48 |  |  |  |  |  |  |
| **Prevention category** | | | | | | | |
| Primary | 15 |  |  |  |  |  |  |
| Secondary | 21 |  |  |  |  |  |  |
| Both | 18 |  |  |  |  |  |  |
| **Trial design characteristics** | | | | | | | |
| Follow-up period, years | 54 | 3.87 | 1.83 | 1.5 | 10 | 0 | 0 |
| **Baseline lipid measurements** | | | | | | | |
| ΔLDLc, mg/dL | 54 | -36.91 | 16.57 | -75 | 1.4 | 0 | 0 |
| ΔremC, mg/dL | 47 | -4.37 | 5.69 | -21.82 | 13.8 | 7 | 12.96 |
| ΔTG, mg/dL | 47 | -16.27 | 12.87 | -50 | 12 | 7 | 12.96 |
| ΔHDLc, mg/dL | 48 | 1.53 | 1.63 | -2.9 | 6.98 | 6 | 11.11 |
| **Treatment-related lipid measurements** | | | | | | | |
| LDLc, mg/dL | 53 | 136.57 | 27.71 | 87.9 | 195.56 | 1 | 1.85 |
| remC, mg/dL | 53 | 30.72 | 7.06 | 13.05 | 60 | 1 | 1.85 |
| TG, mg/dL | 53 | 151 | 25.32 | 113.27 | 264 | 1 | 1.85 |
| HDLc, mg/dL | 53 | 45.82 | 6.05 | 35.96 | 59.55 | 1 | 1.85 |

# Supplementary Table 4. Stepwise covariate search.

**Step 1:** statistically significant covariates (Wald test, p-value < 0.05) after addition to the base model one at a time (log(RR) ~ Therapy + COVi).

**Step 2:** statistically significant interactions of selected covariate at Step 1 (Wald test, p-value < 0.05) after addition to the corresponding model at Step 1 (log(RR) ~ Therapy + COVi + COVi:COVj).

**Step 3:** a union model with a combination of all selected covariates at Steps 1-2.

**Final model:** the results of backward covariate search of the model at Stage 3.

| Event | Step 1 | Step 2 | Step 3 | Final model |
| --- | --- | --- | --- | --- |
| CM | ΔLDLc | - | ΔLDLc | ΔLDLc |
| CR | ΔLDLc | ΔLDLc + ΔLDLc:HDLc | ΔLDLc + ΔremC + ΔLDLc:HDLc | ΔLDLc |
|  | ΔremC | - |  |  |
| CVM | HDLc | HDLc + HDLc:Age | HDLc + Age + Smokers + HDLc:Age + Smokers:Age + HDLc:Smokers | HDLc |
|  | HDLc | HDLc + HDLc:Smokers |  |  |
|  | Age | - |  |  |
|  | Smokers | Smokers + Smokers:Age |  |  |
| fMI | - | - | - | - |
| fST | RD | - | RD | RD |
| HF | remC | remC | remC | remC |
| hST | - | - | - | - |
| iST | Diabetes | - | Diabetes + Smokers + Hypertension + RD | Hypertension |
|  | Smokers | - |  |  |
|  | Hypertension | - |  |  |
|  | RD | - |  |  |
| MI | HDLc | HDLc + HDLc:Smokers | HDLc + HDLc:Smokers | HDLc |
| nfMI | ΔLDLc | ΔLDLc + ΔLDLc:RD | ΔLDLc + ΔLDLc:RD | ΔLDLc + ΔLDLc:RD |
| nfST | - | - | - | - |
| ST | Prevention | Prevention + Prevention:AGE | Prevention + Prevention:AGE | Prevention:AGE |
| TIA | - | - | - | - |
| TM | HDLc | - | HDLc + Males | HDLc |
|  | Males | - |  |  |
| UA | - | - | - | - |

# Supplementary Figure 1. Results of meta-regression modeling of event rates in the control groups for each single CV event.

Red lines with shaded areas indicate fitted meta-regression line with 95% CI. Circles are sized according to the number of patients.


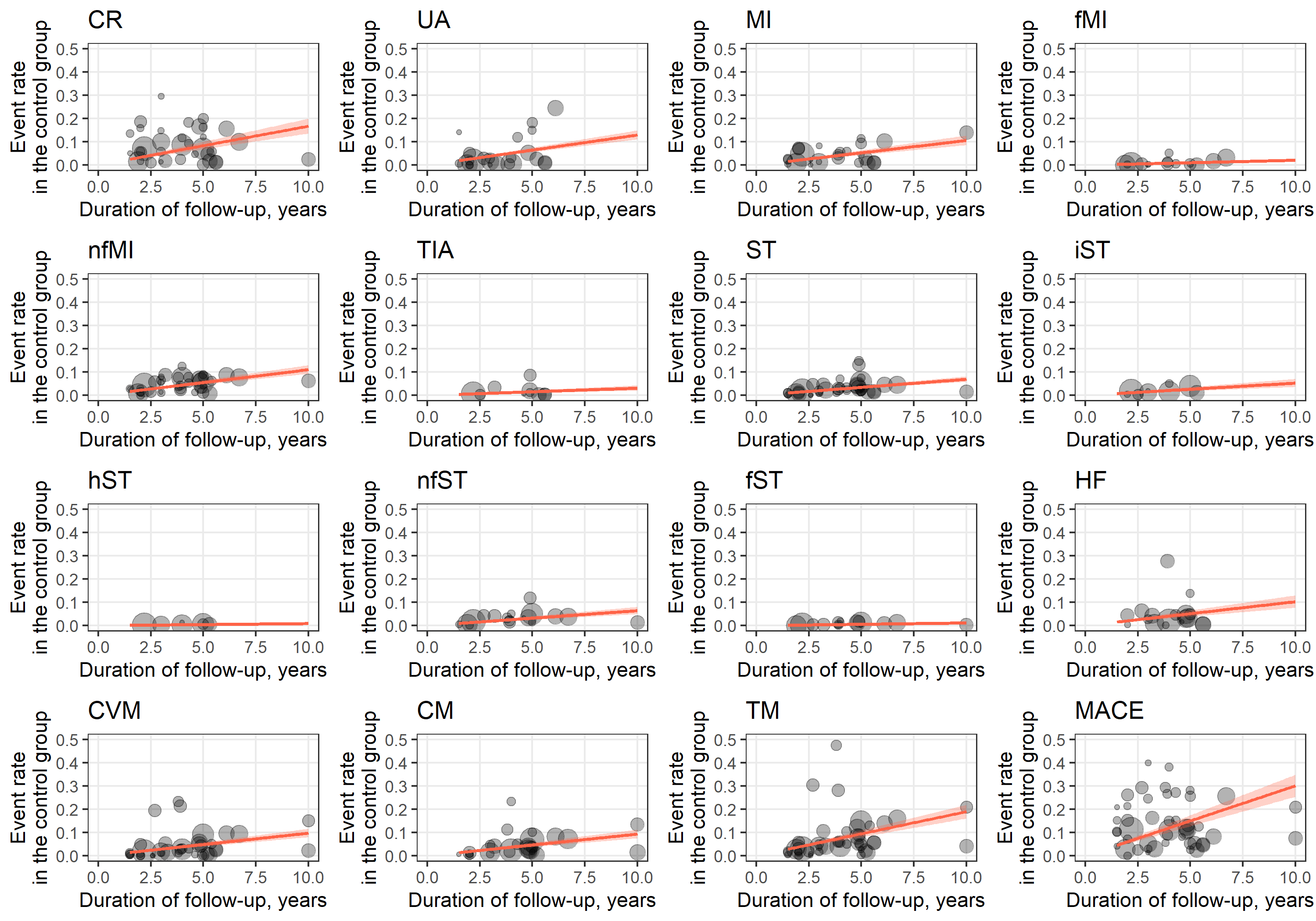


# Supplementary Figure 2. Correlation between lipid (A) and non-lipid (B) covariates.

Scatterplots of each covariate pair with linear regression line with 95% CI visualized in plots on the left side. Pearson correlation value and significance displayed on the right side.


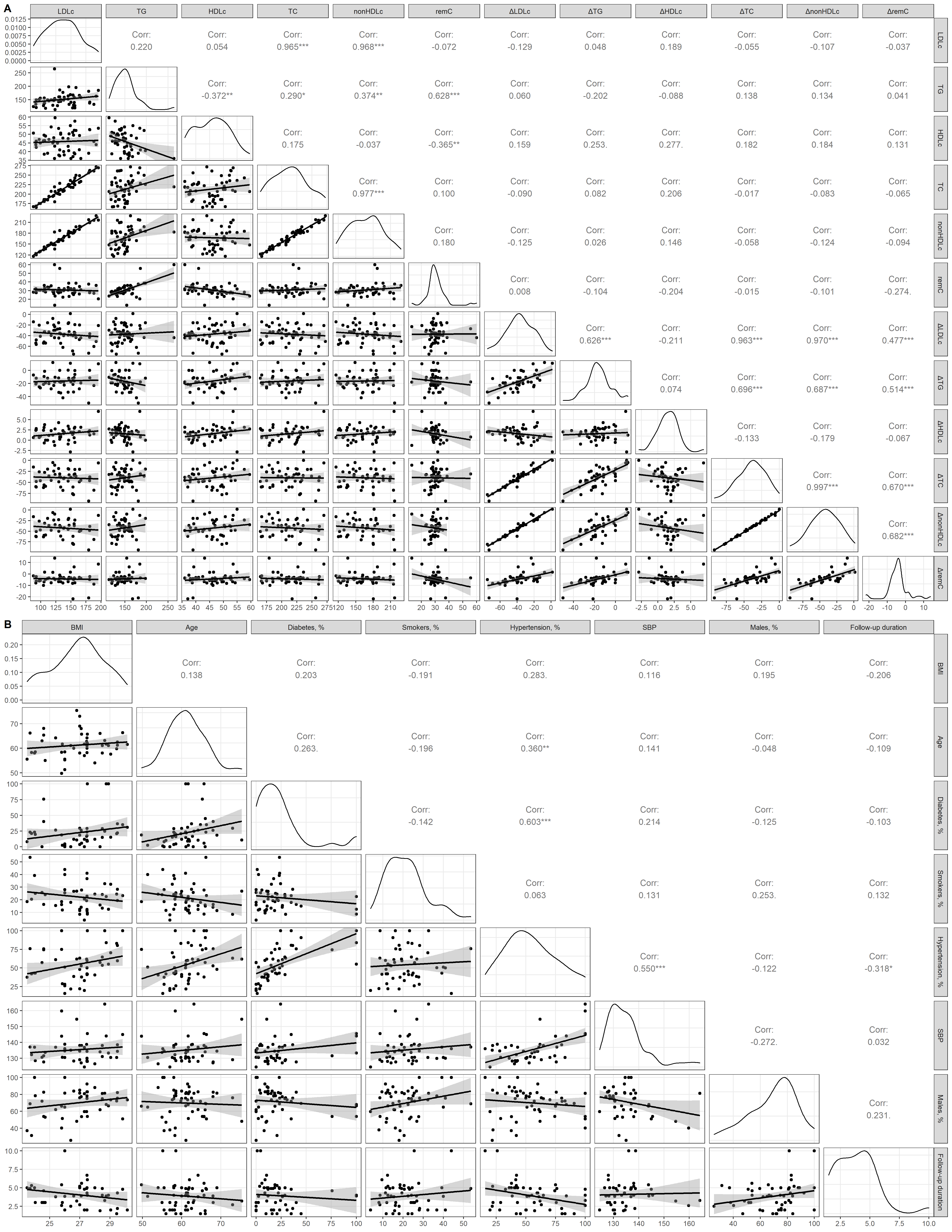


# Supplementary Figure 3. Weighted linear regression of the logarithm of RR and follow-up duration, per CV event.

Red lines depict the weighted regression line with specified adjusted R2 (Adj R2) and p-value (P). Circles are sized according to the study weight.


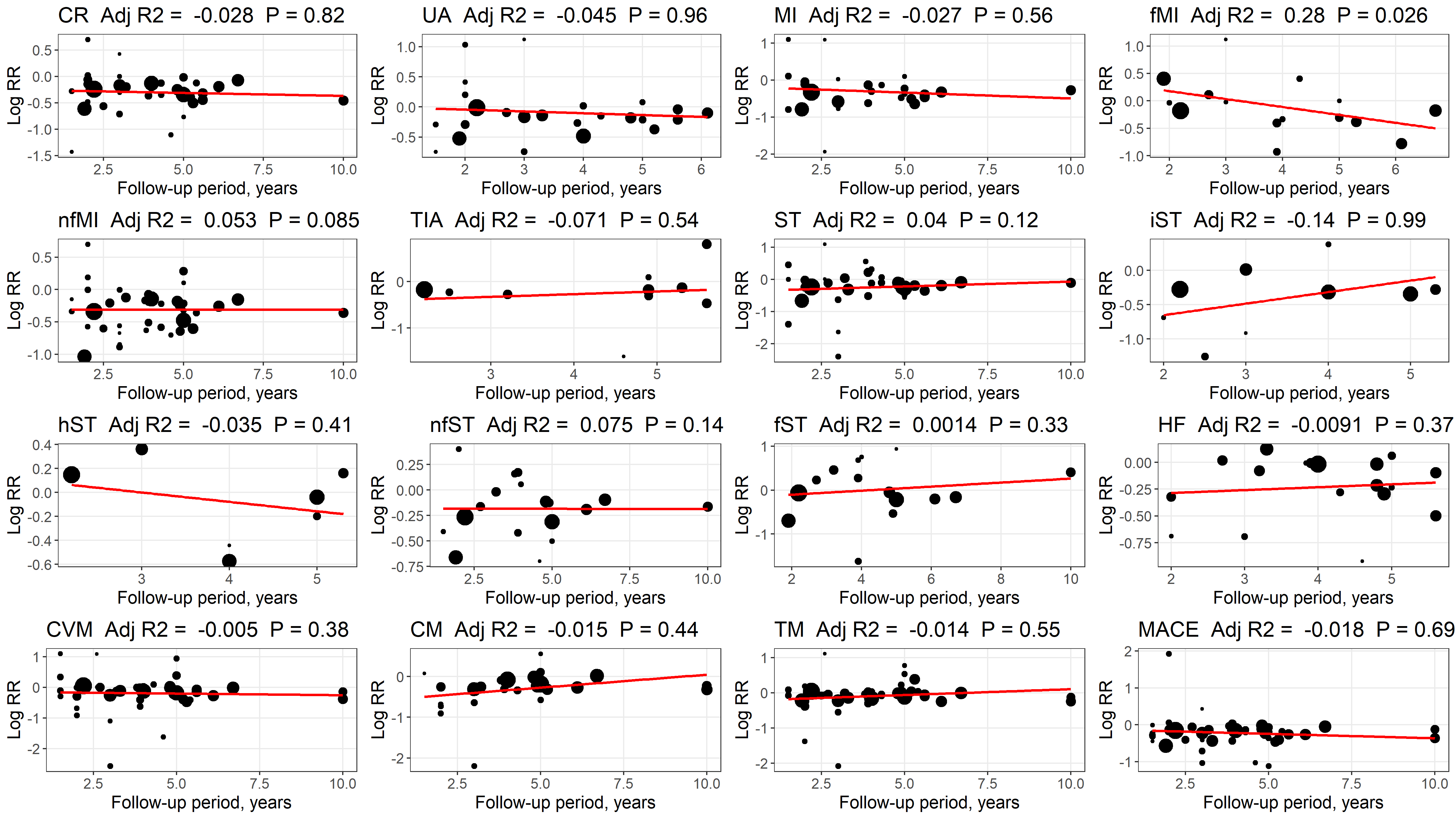


# Supplementary Figure 4. Funnel plots for each CV event.

White triangles represent the region where 95% of the data points should lie in the absence of publication bias. The vertical dashed line represents the meta-analysis mean. Funnel plot asymmetry was detected using Egger’s test.


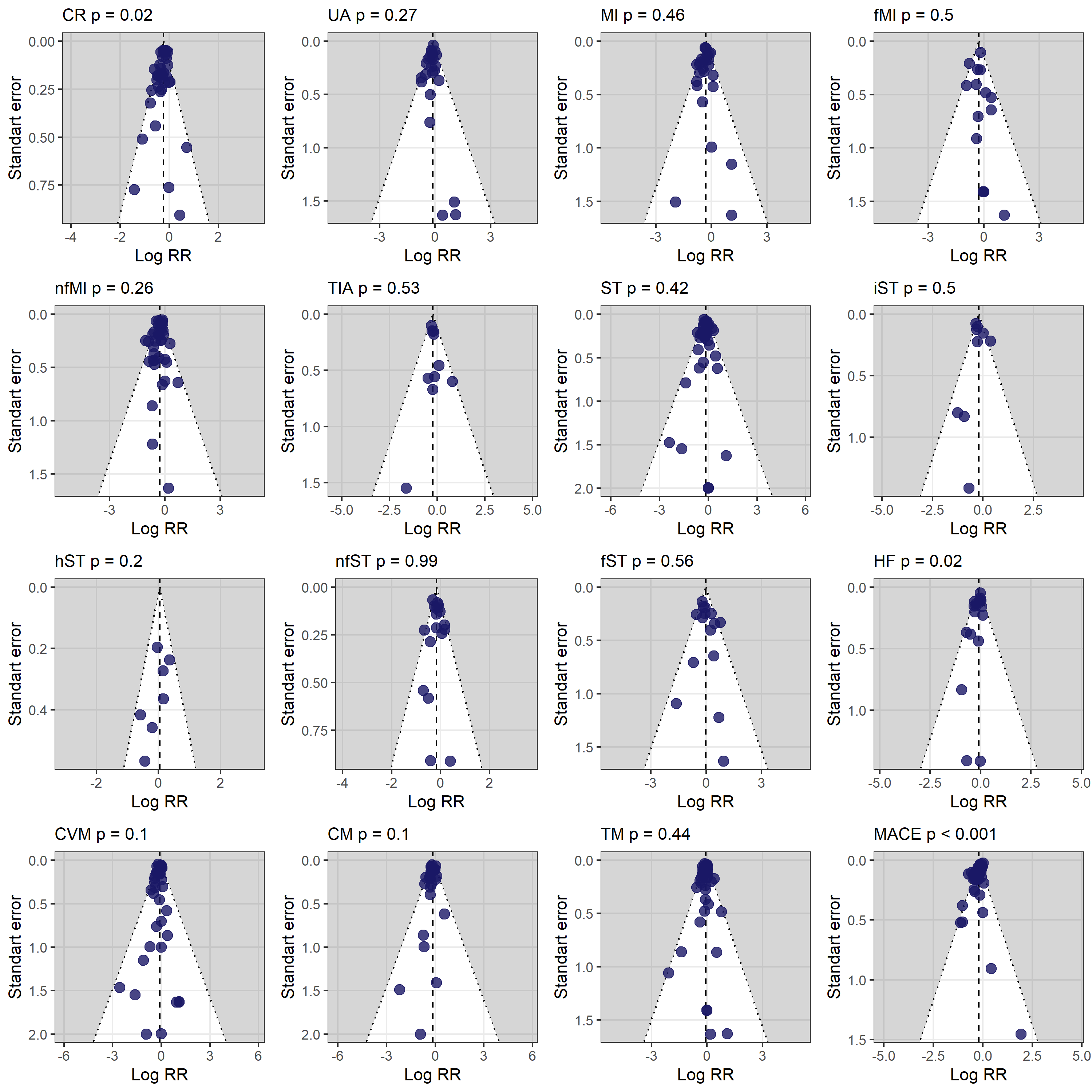


# Supplementary Figure 5. Leave-one-out diagnostics per CV event.

Red points represent potential outliers and influential cases.


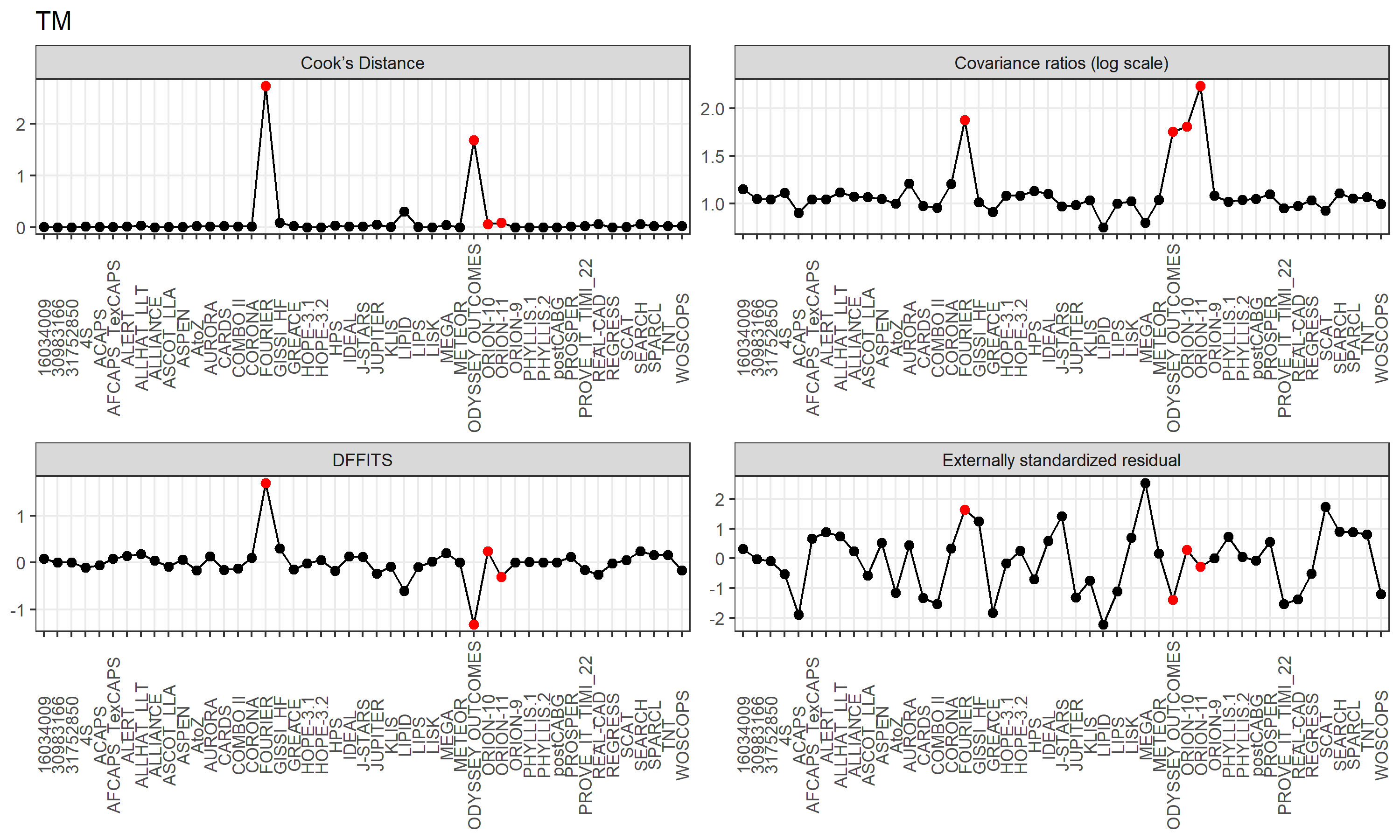

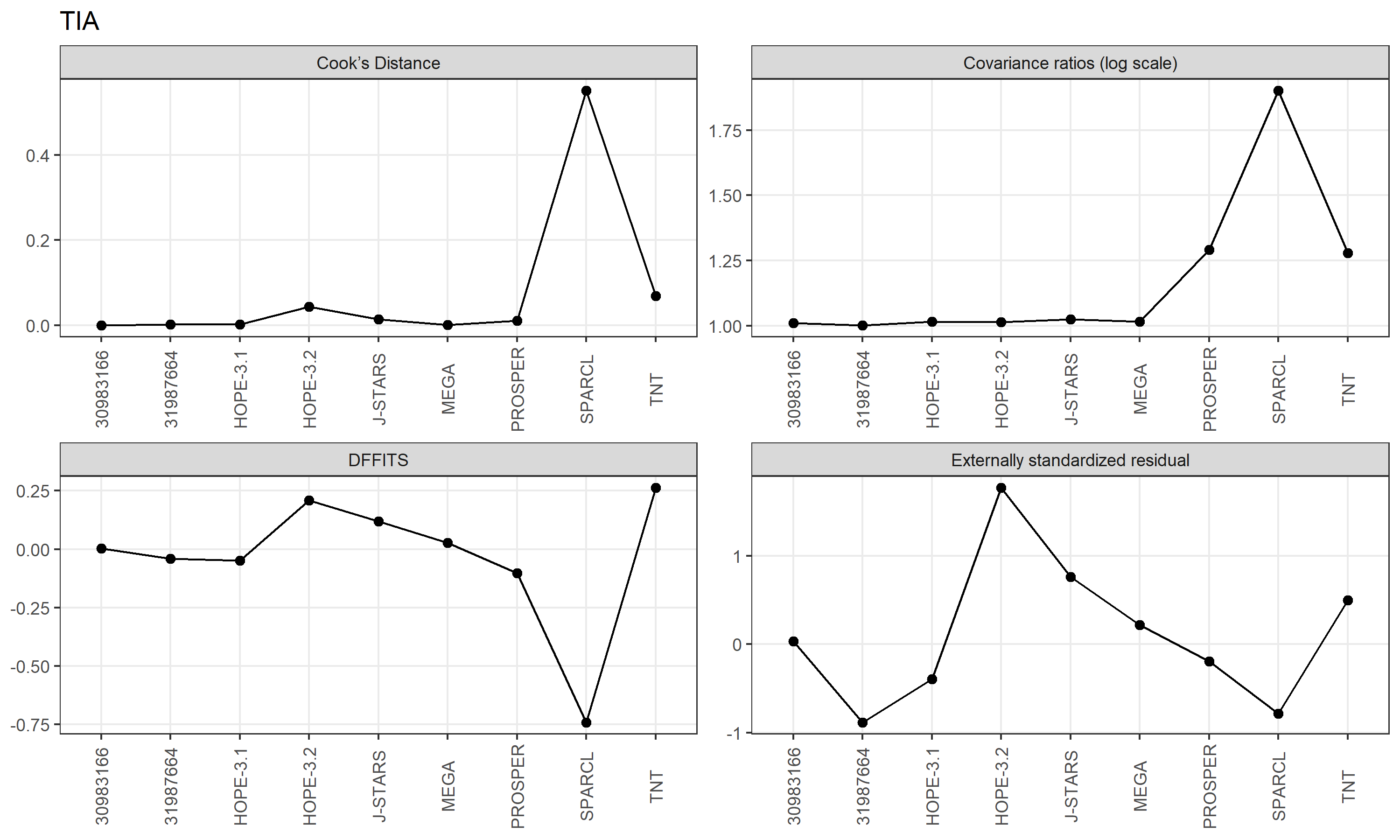

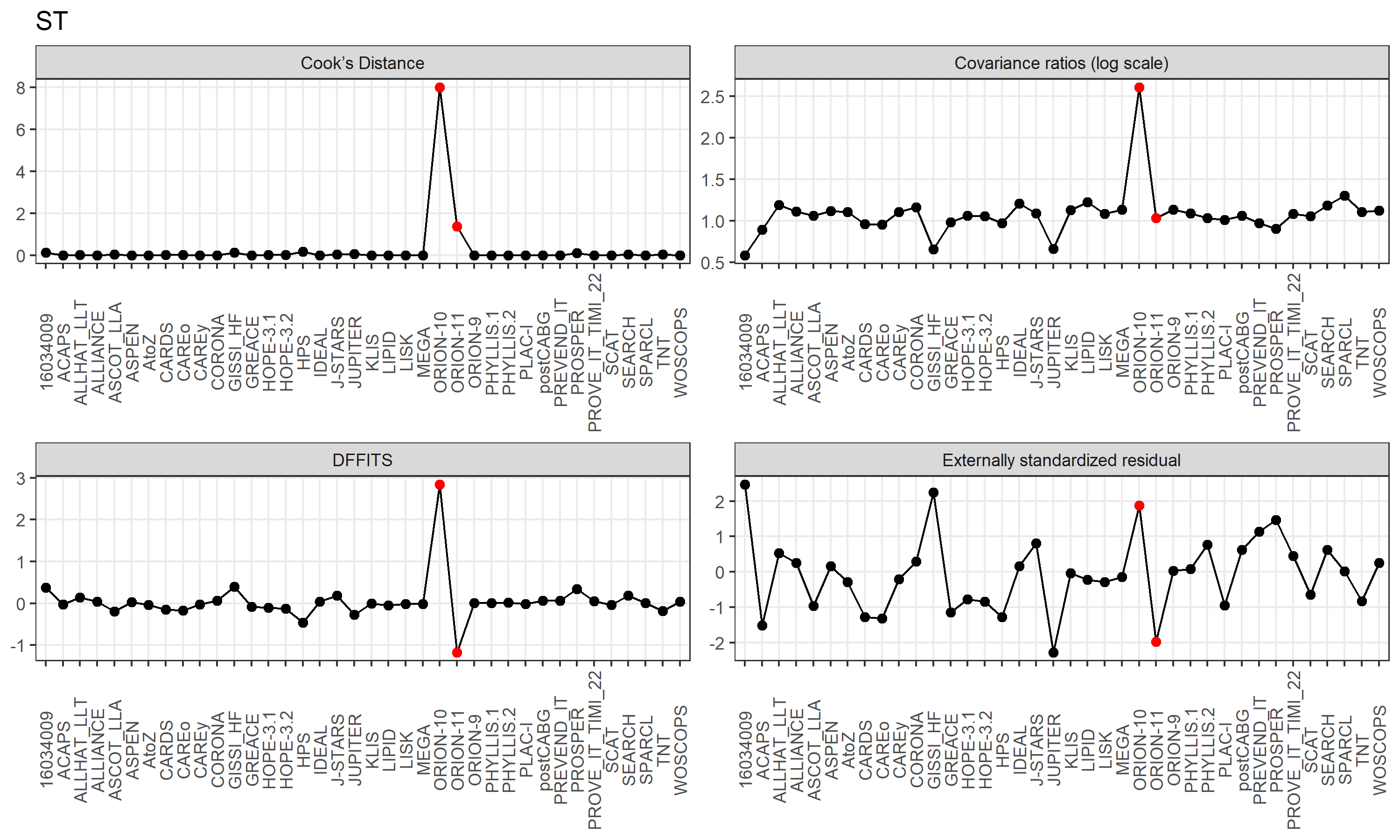

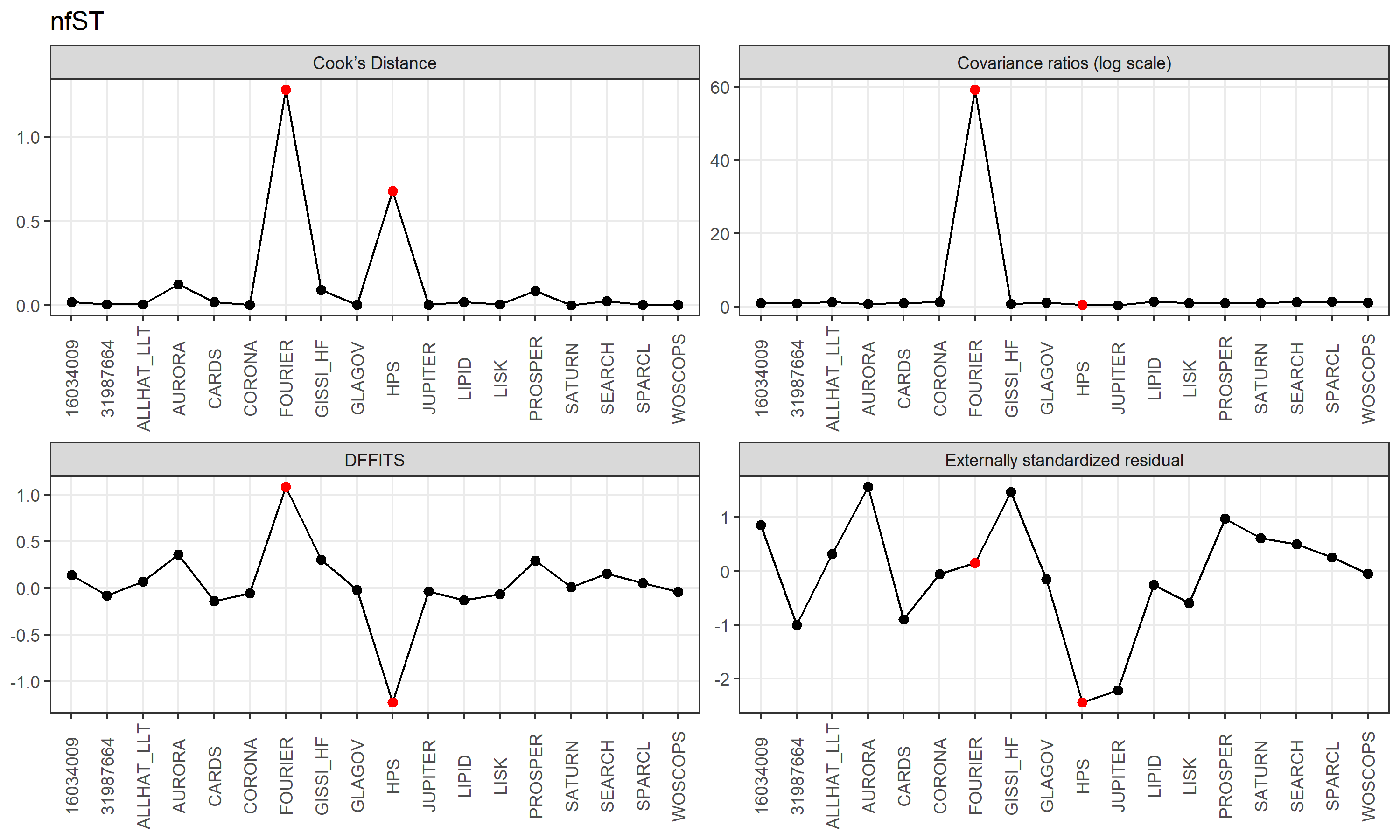

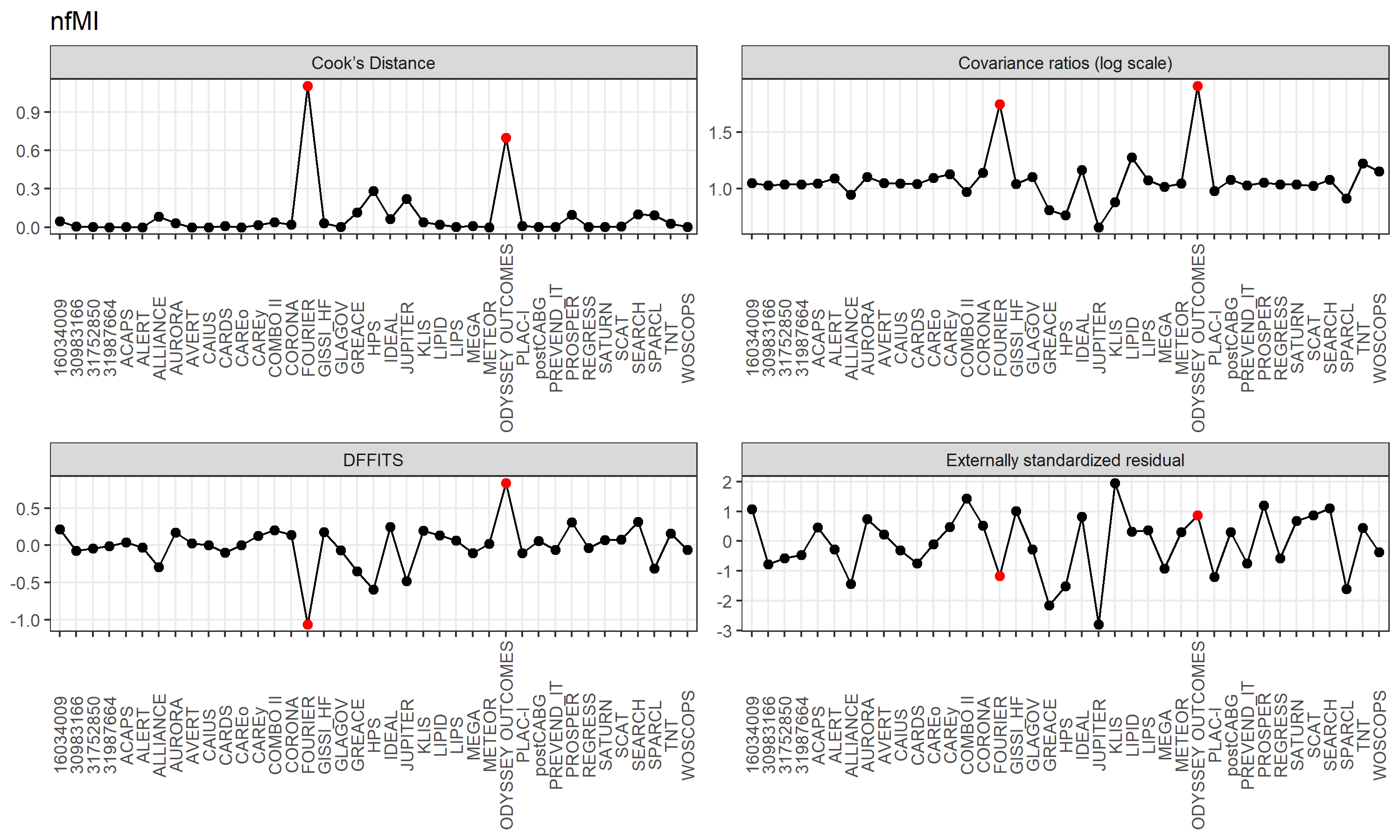

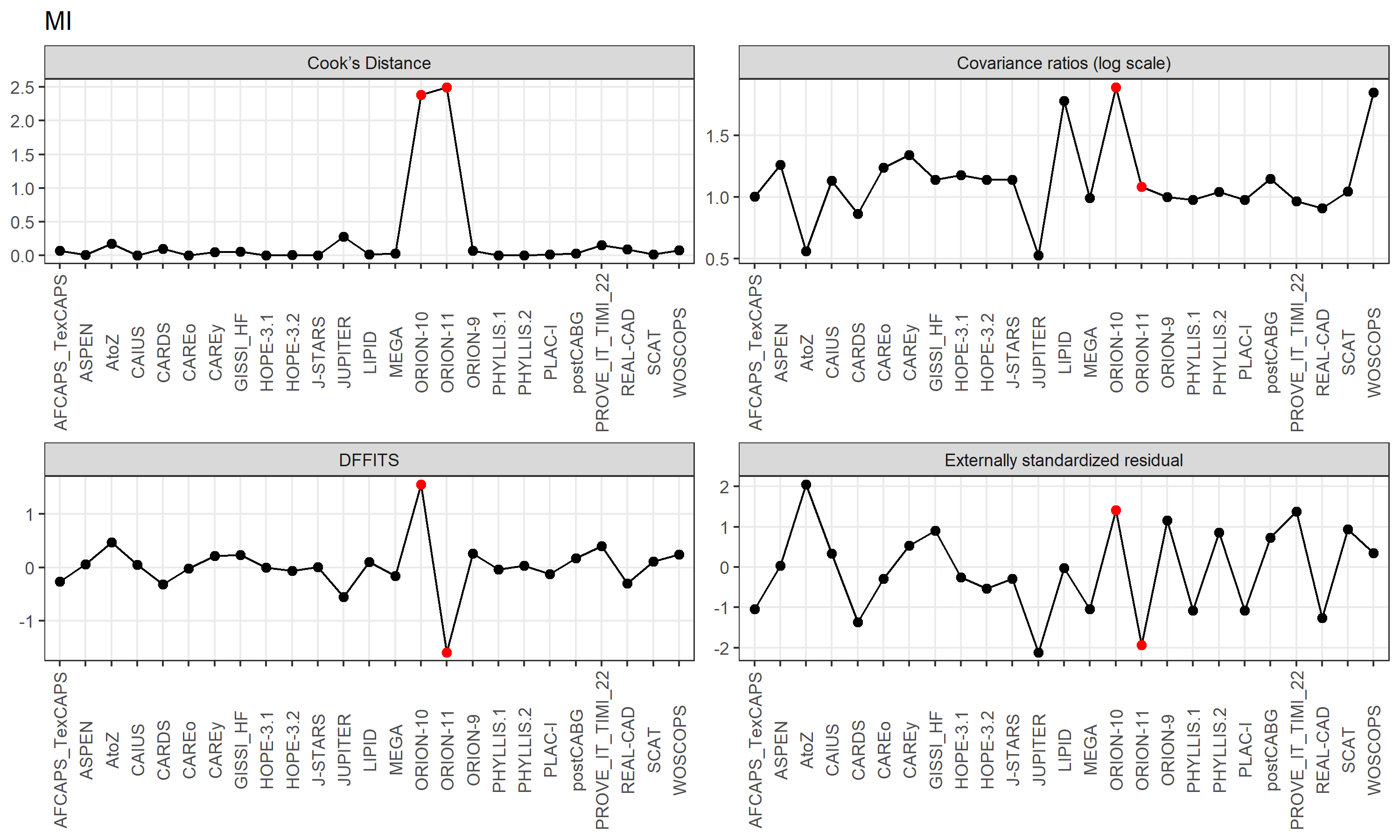

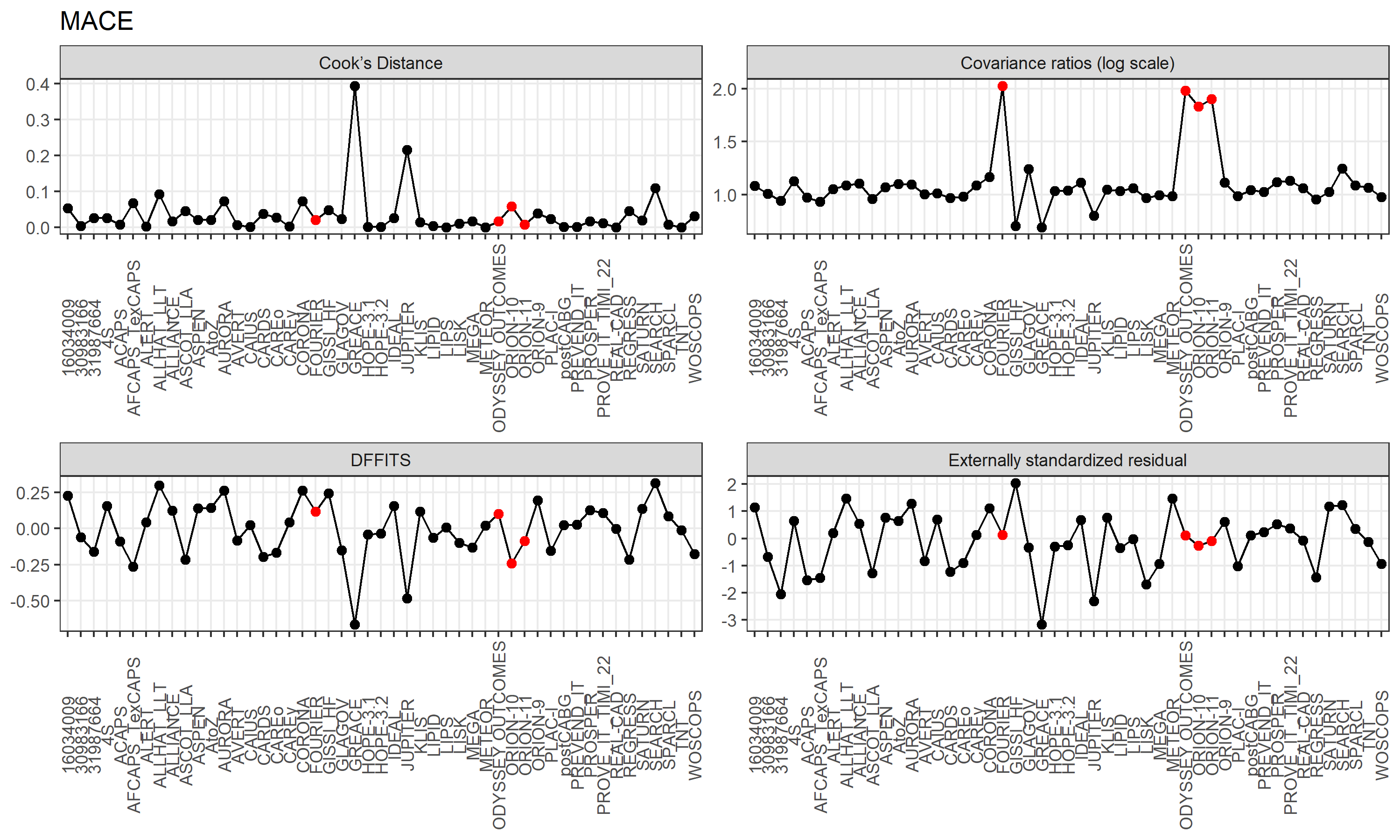

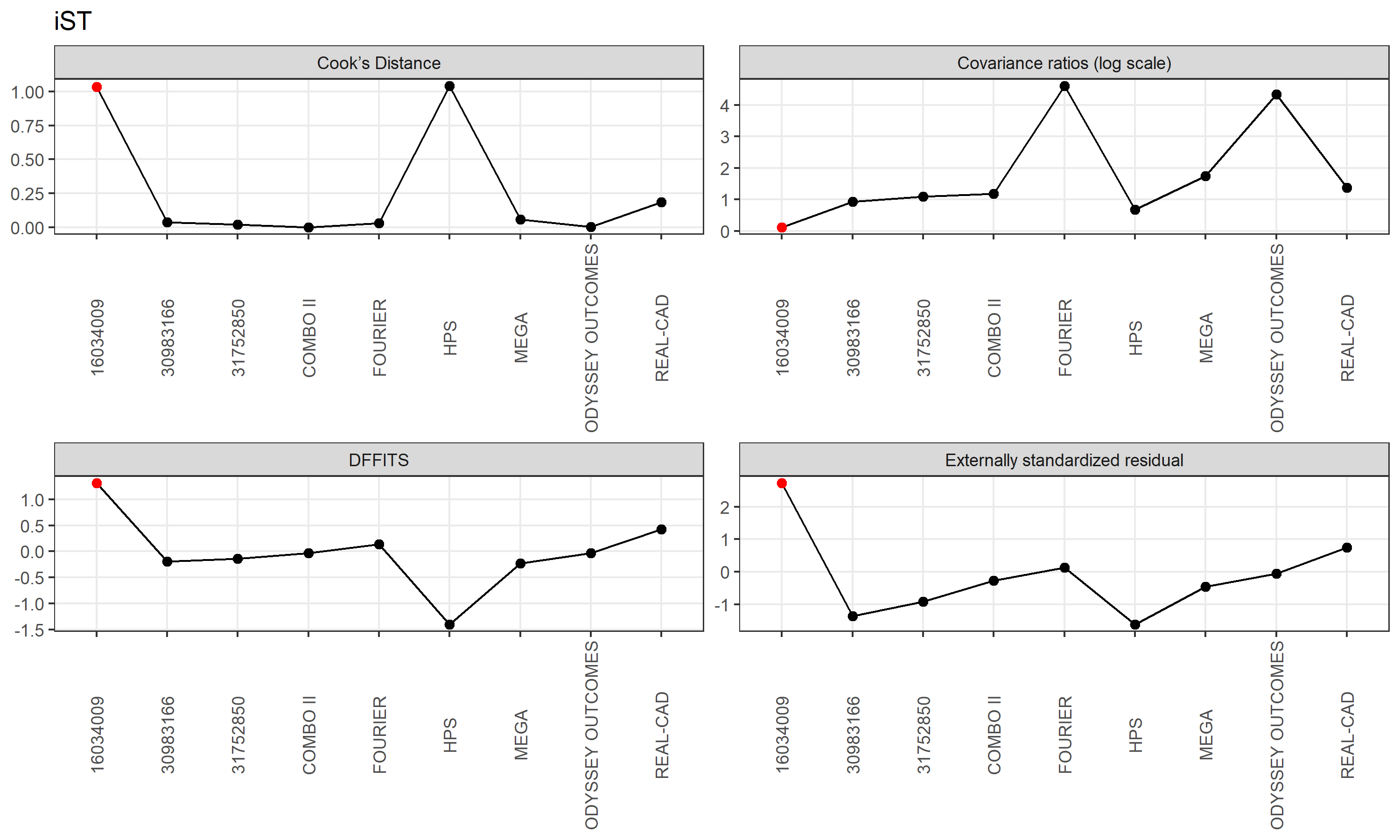

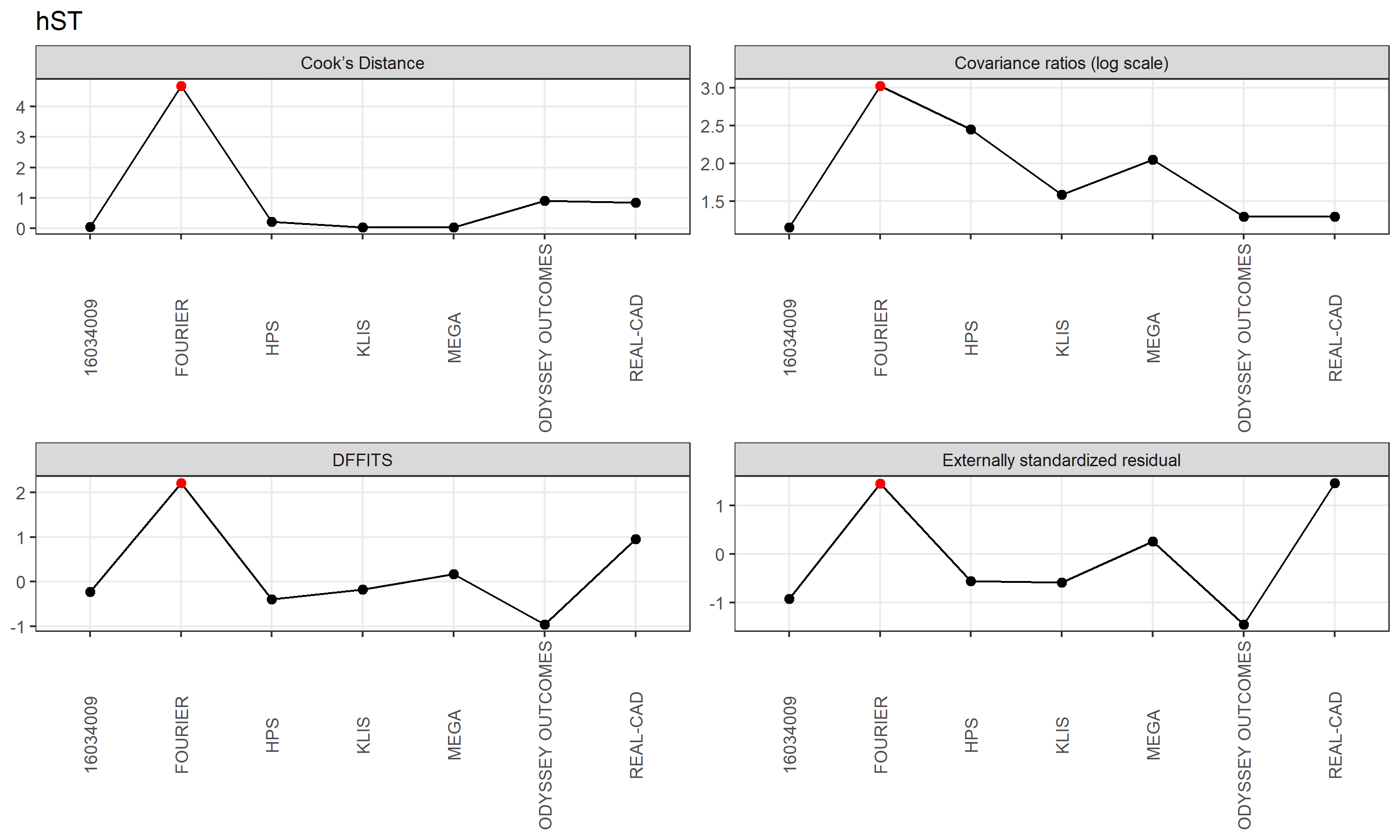

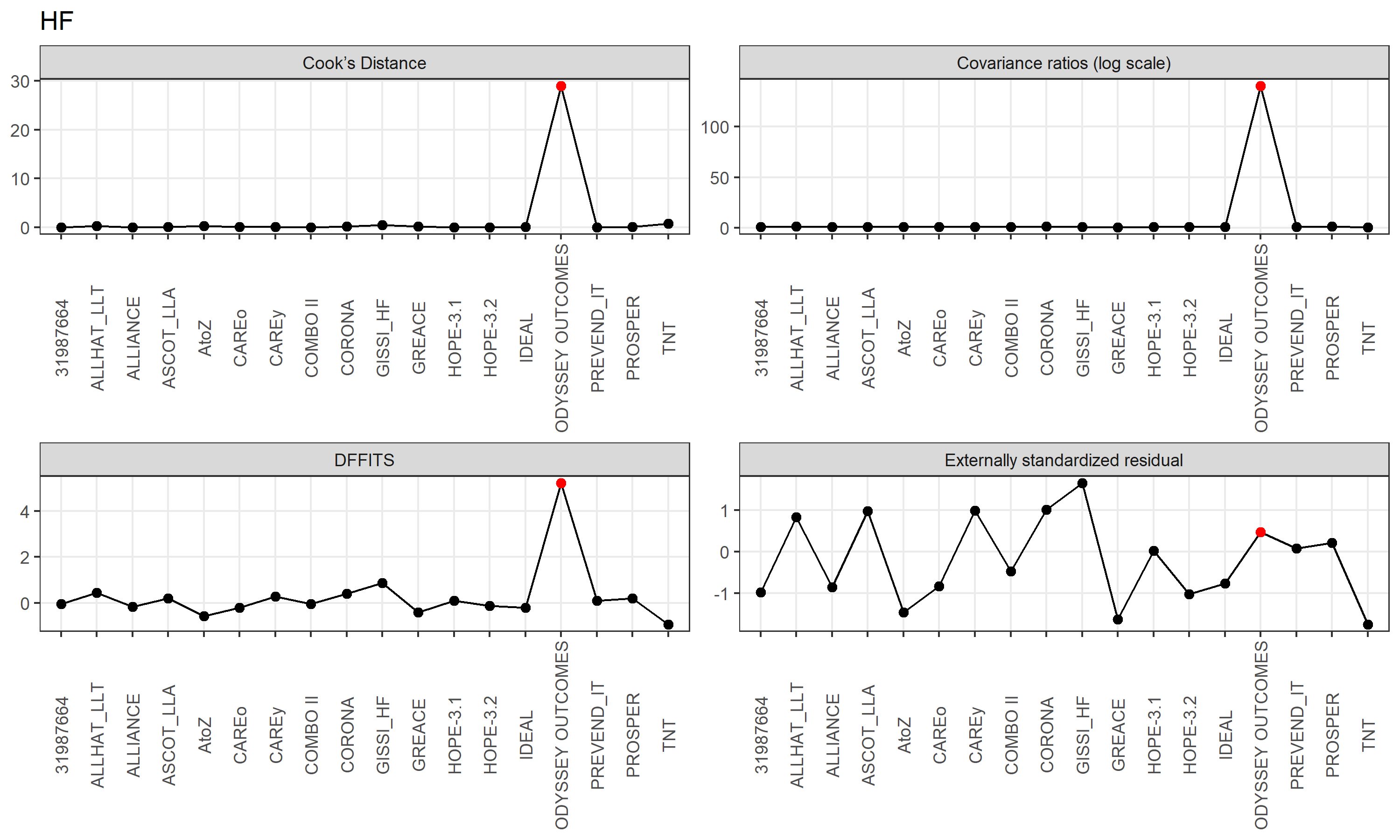

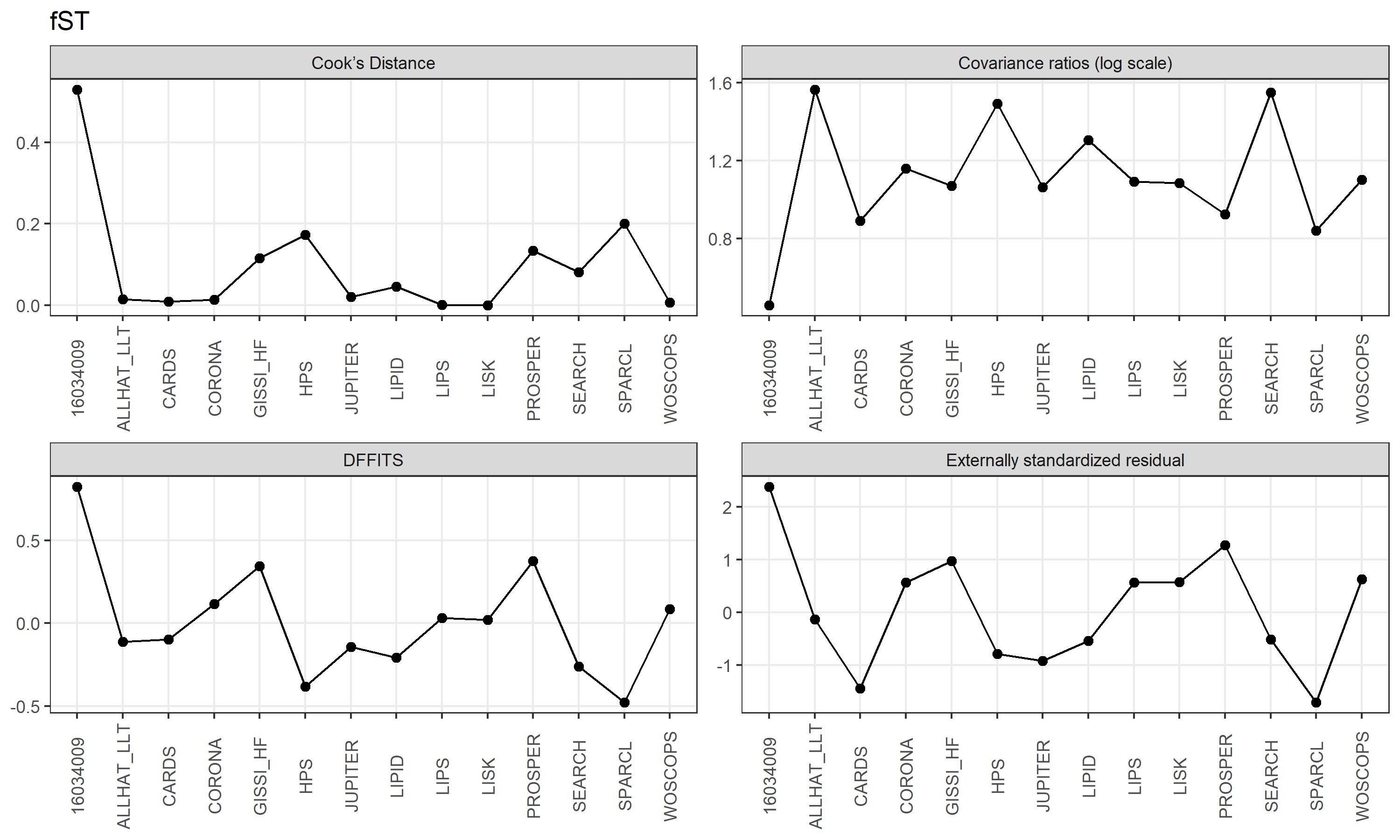

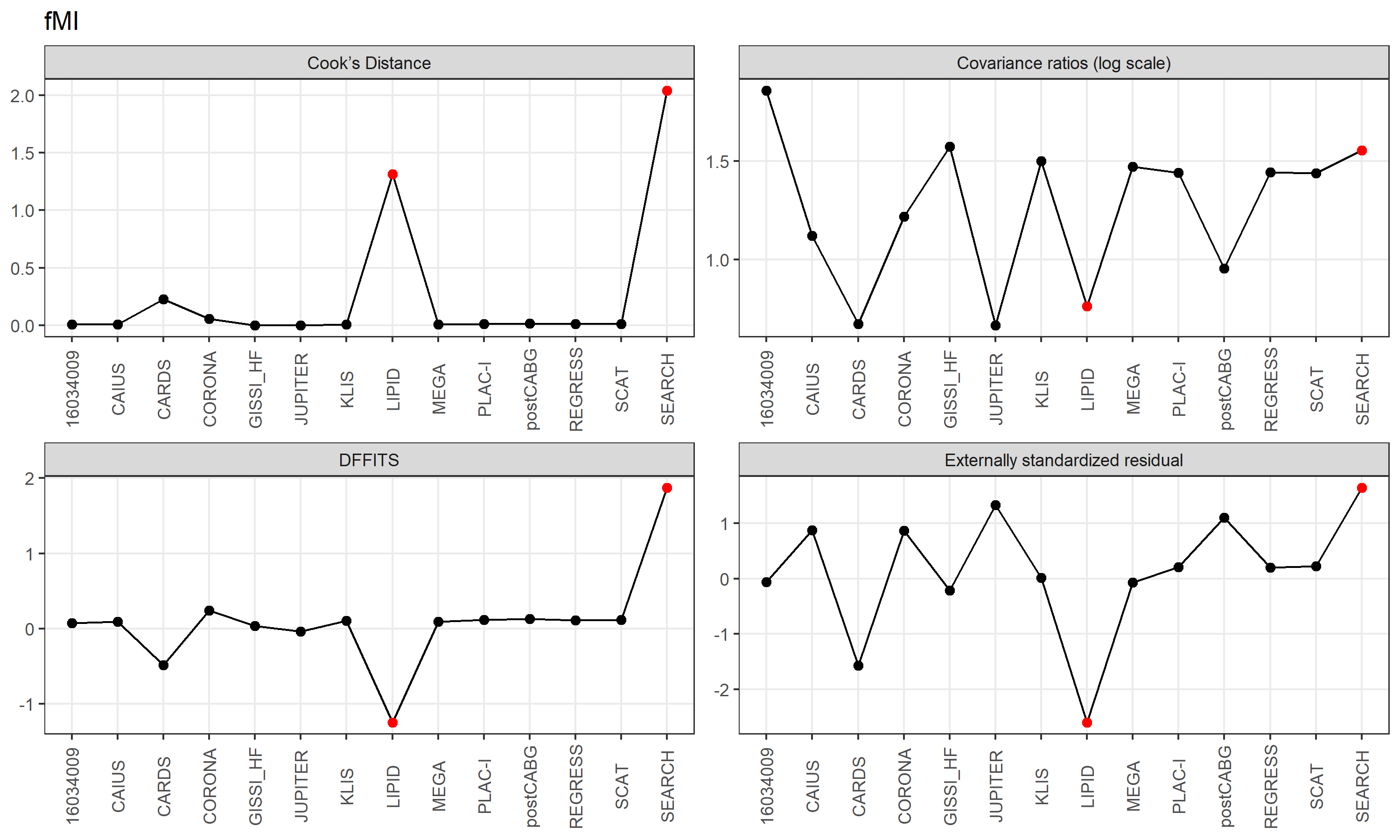

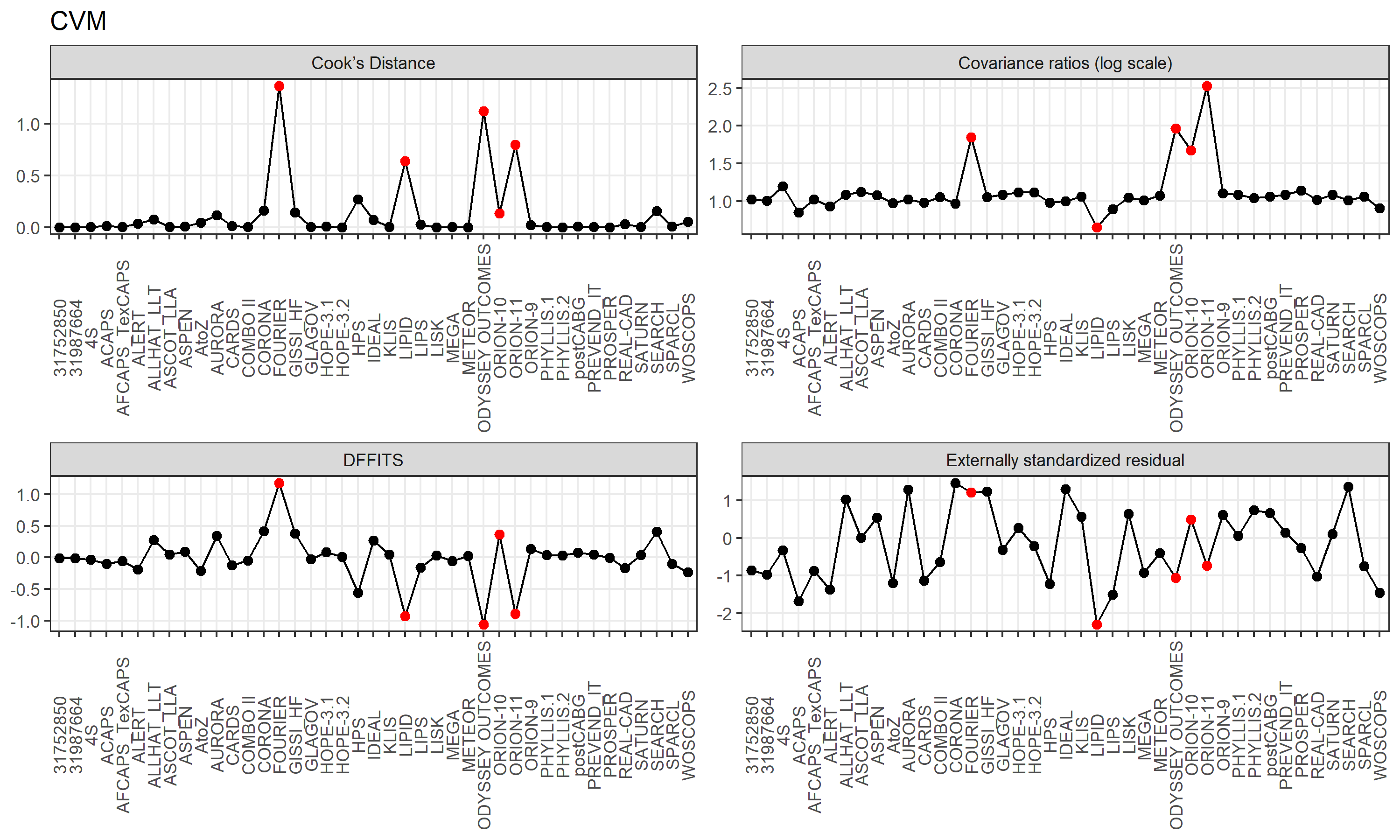

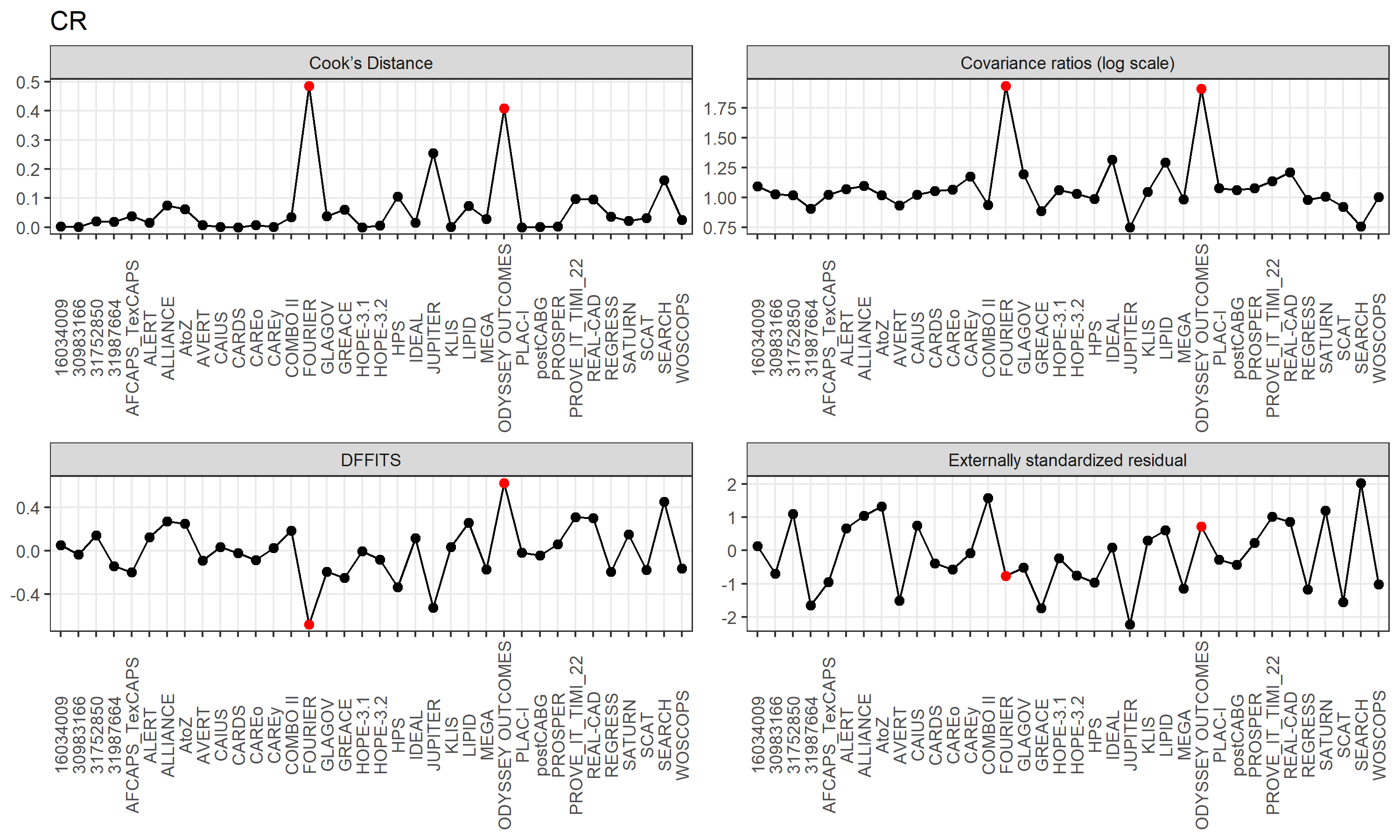

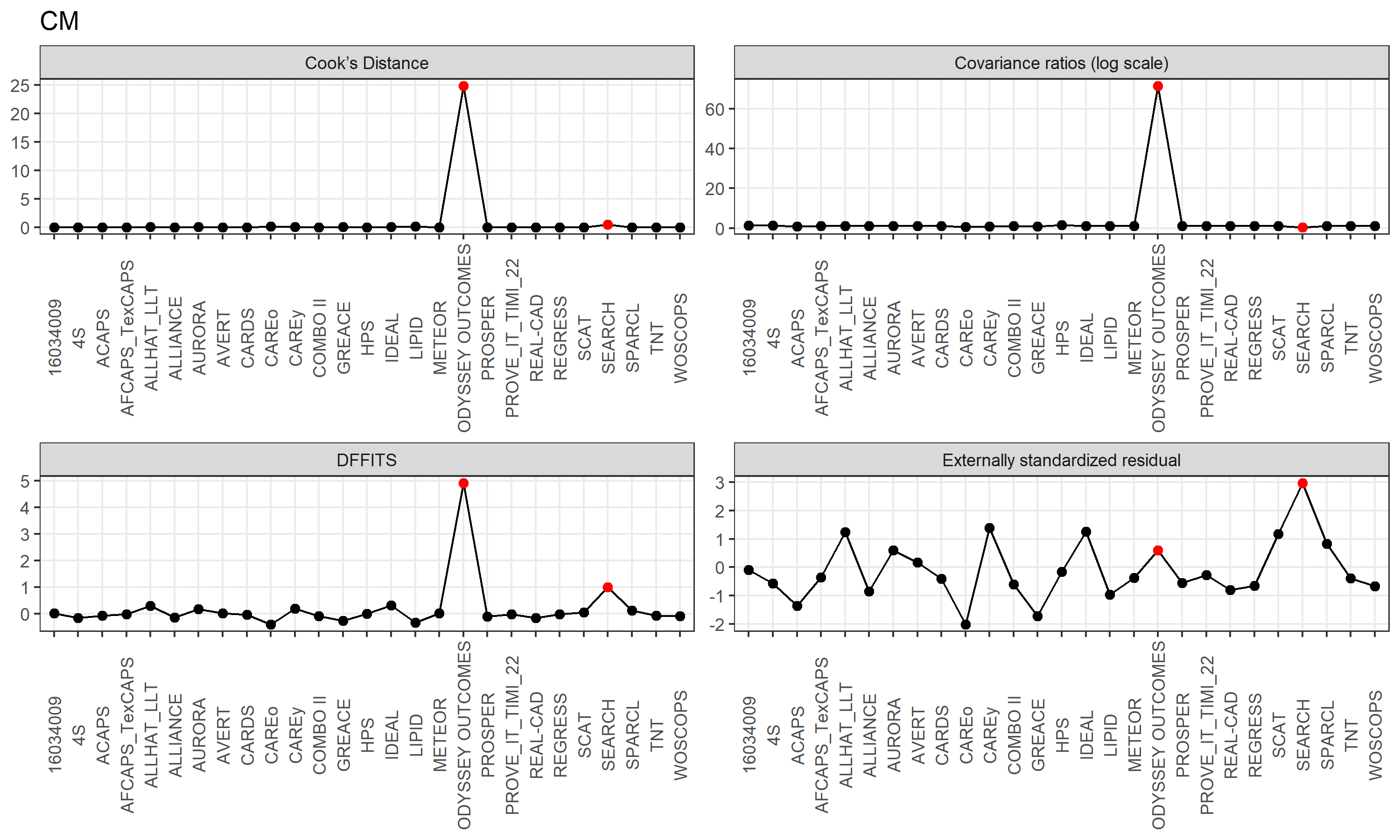

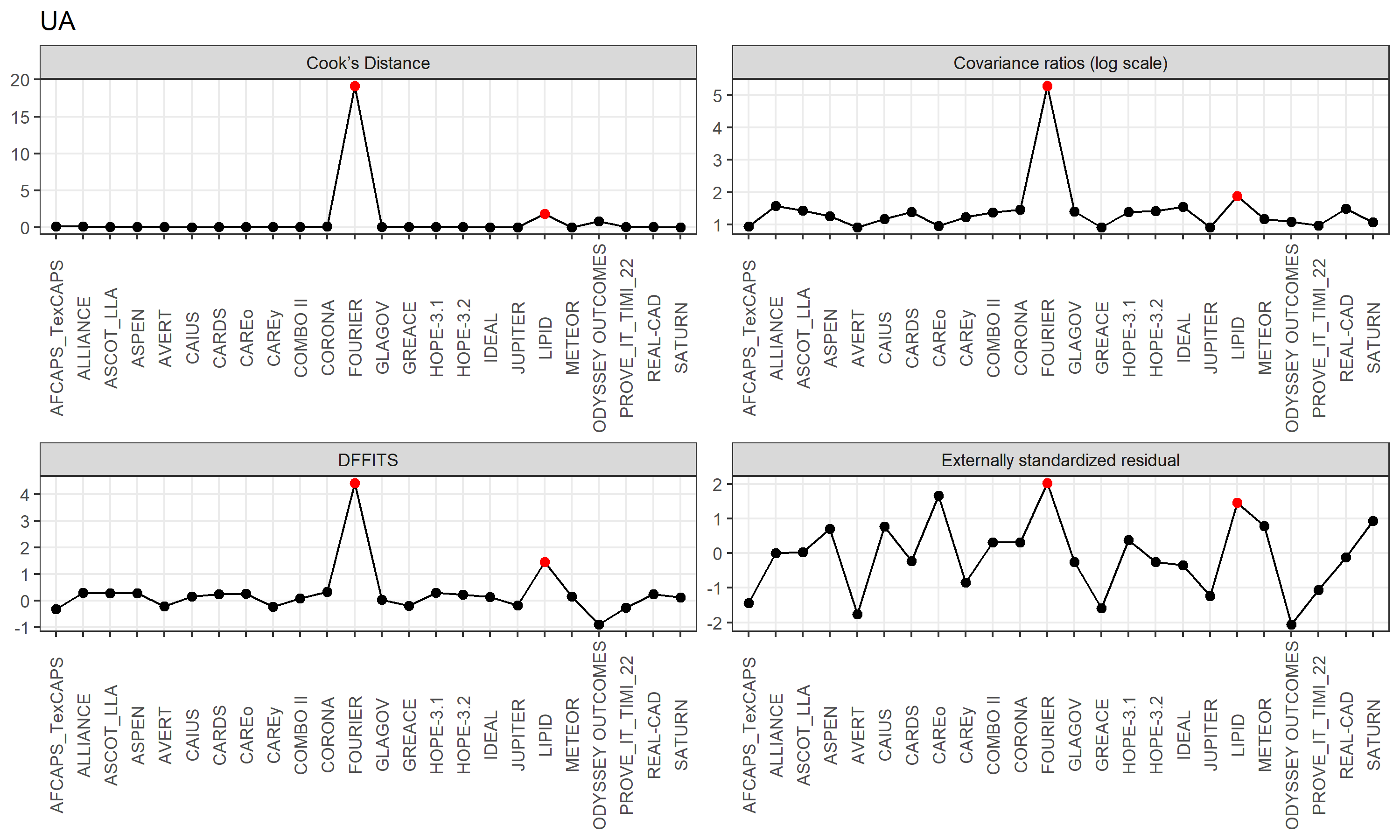


**Supplementary Figure 6. Forest plots, per CV event.**
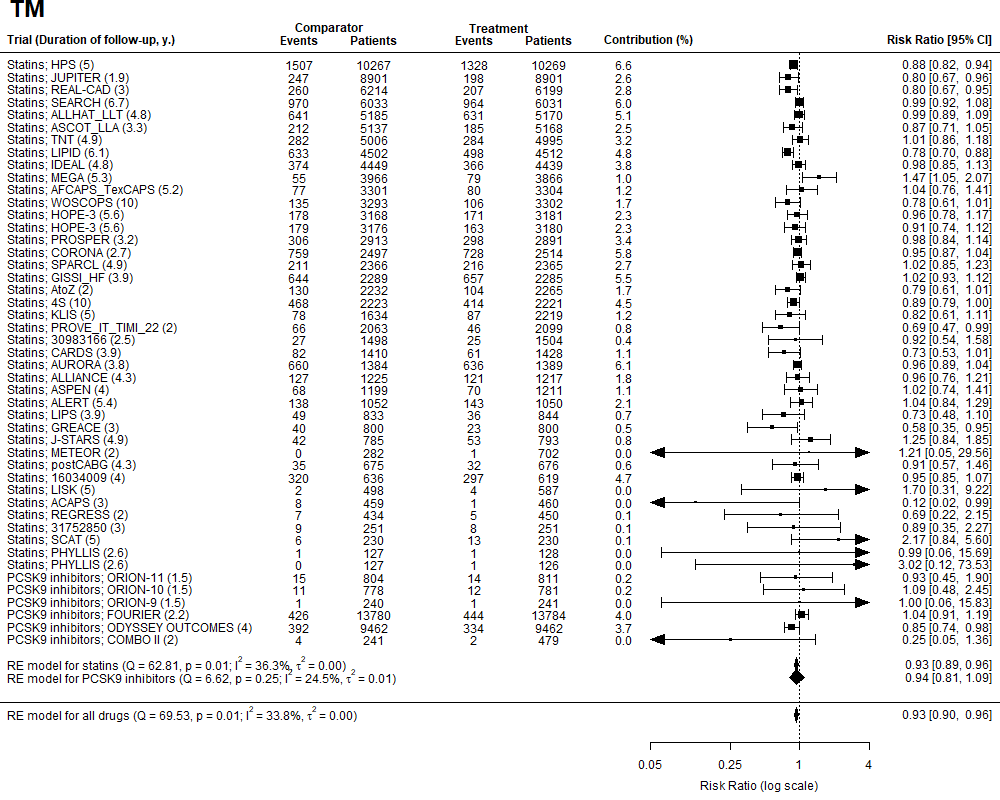

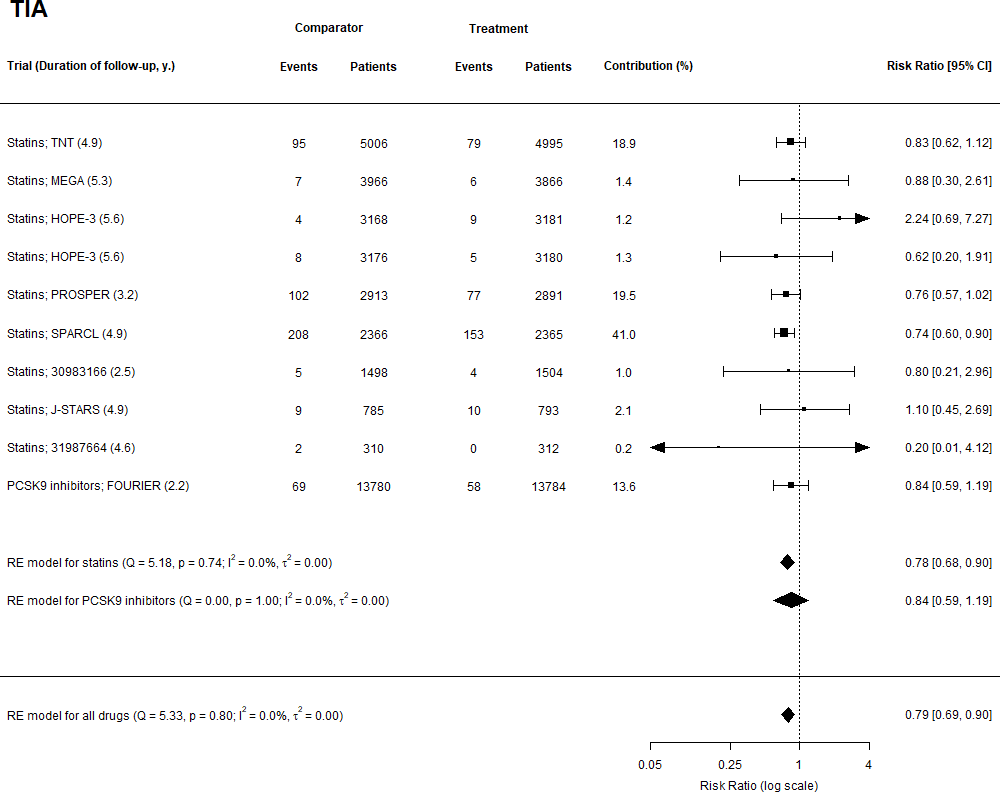

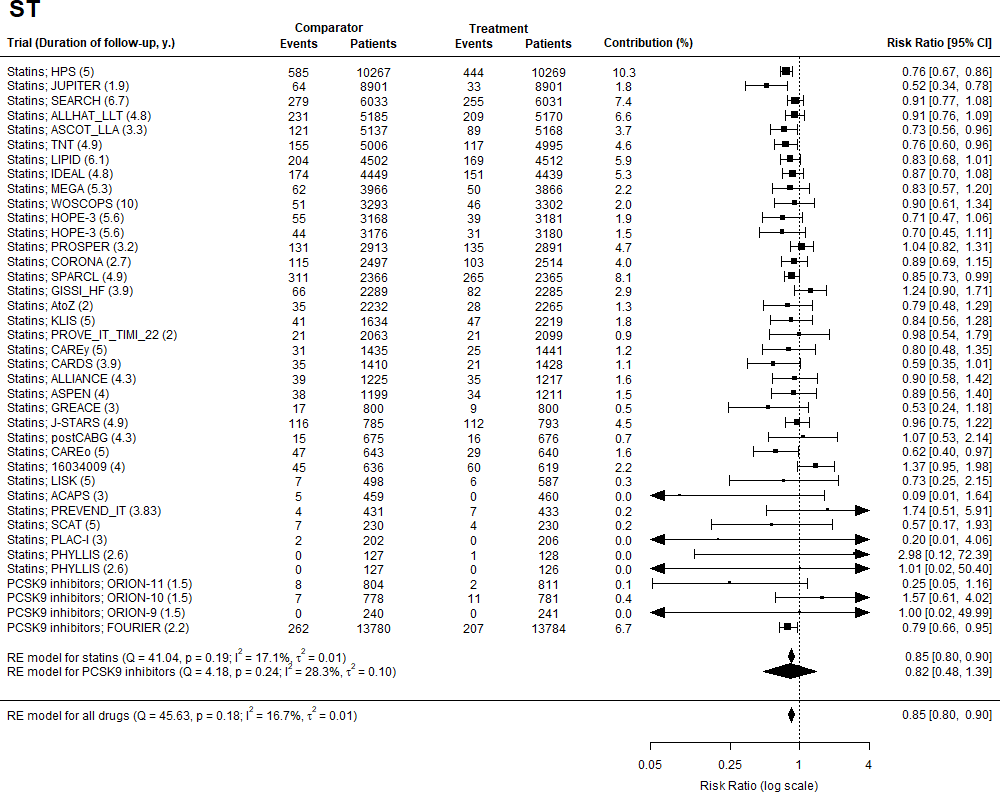

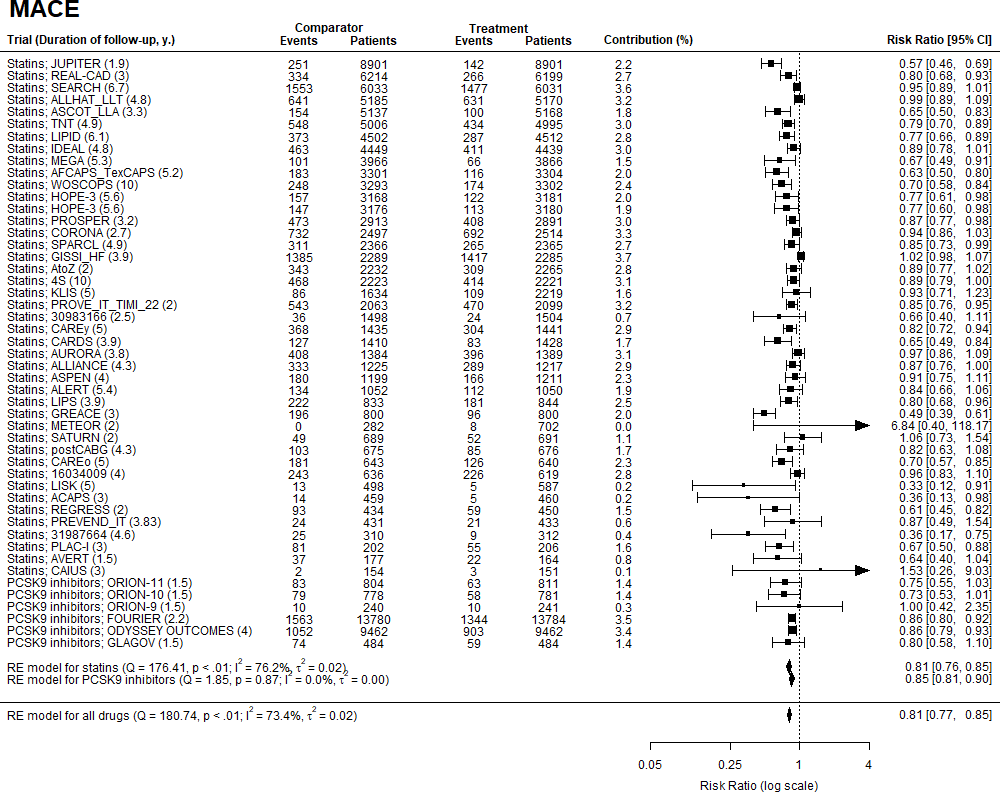

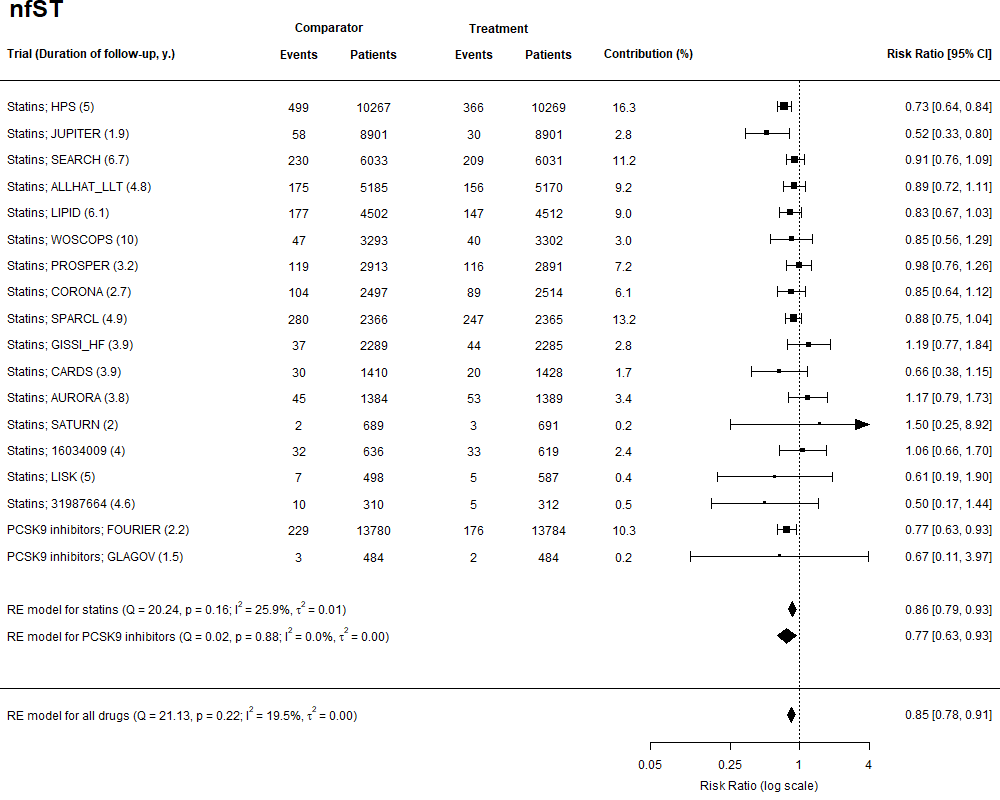

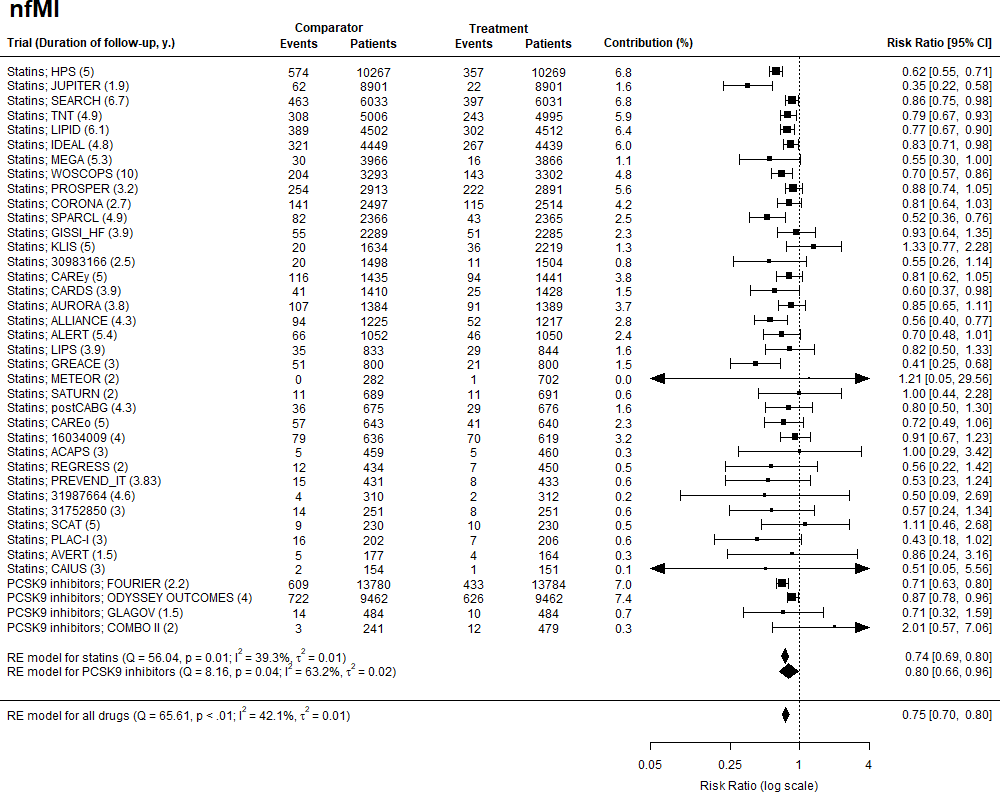

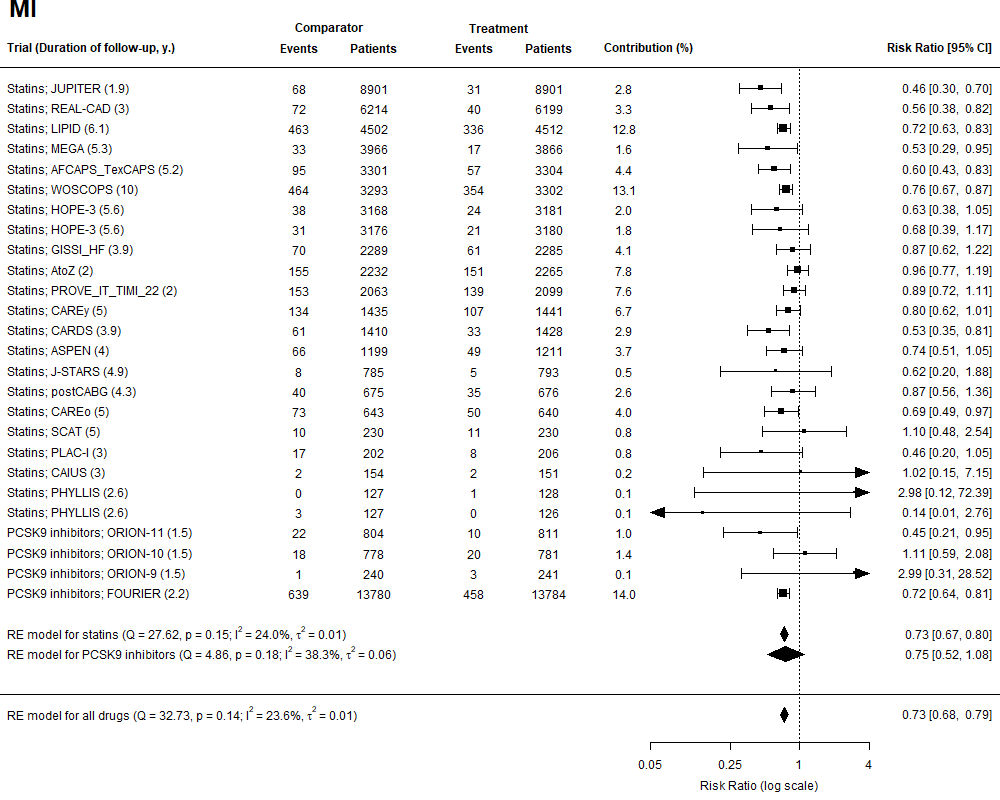

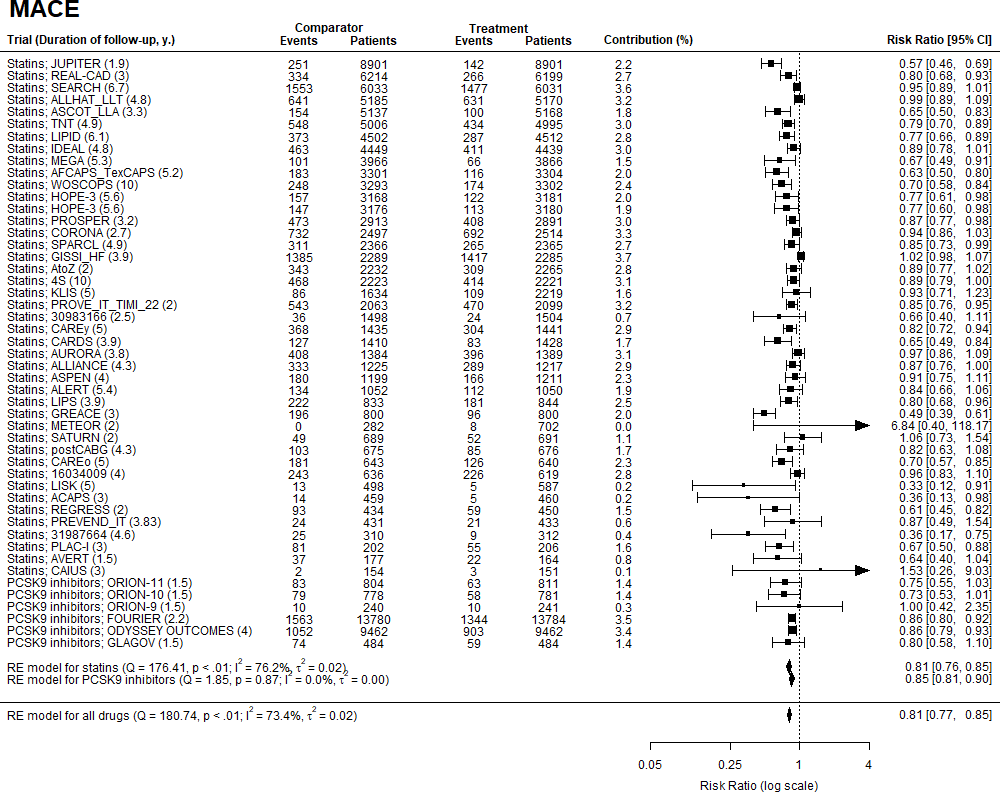

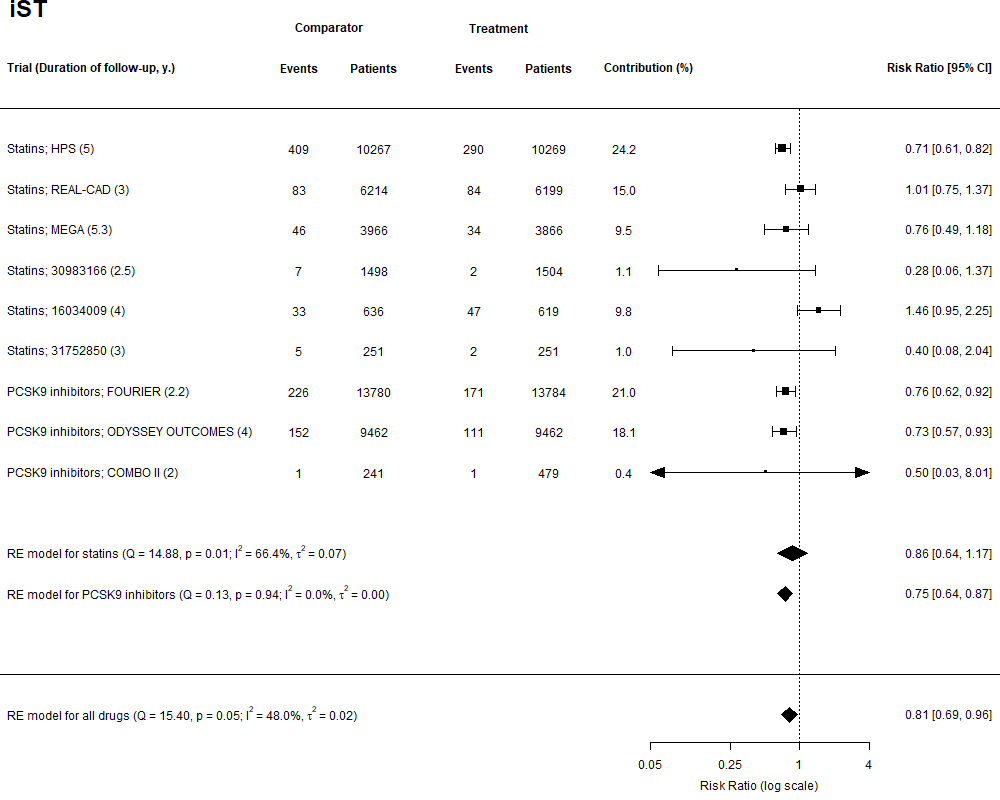

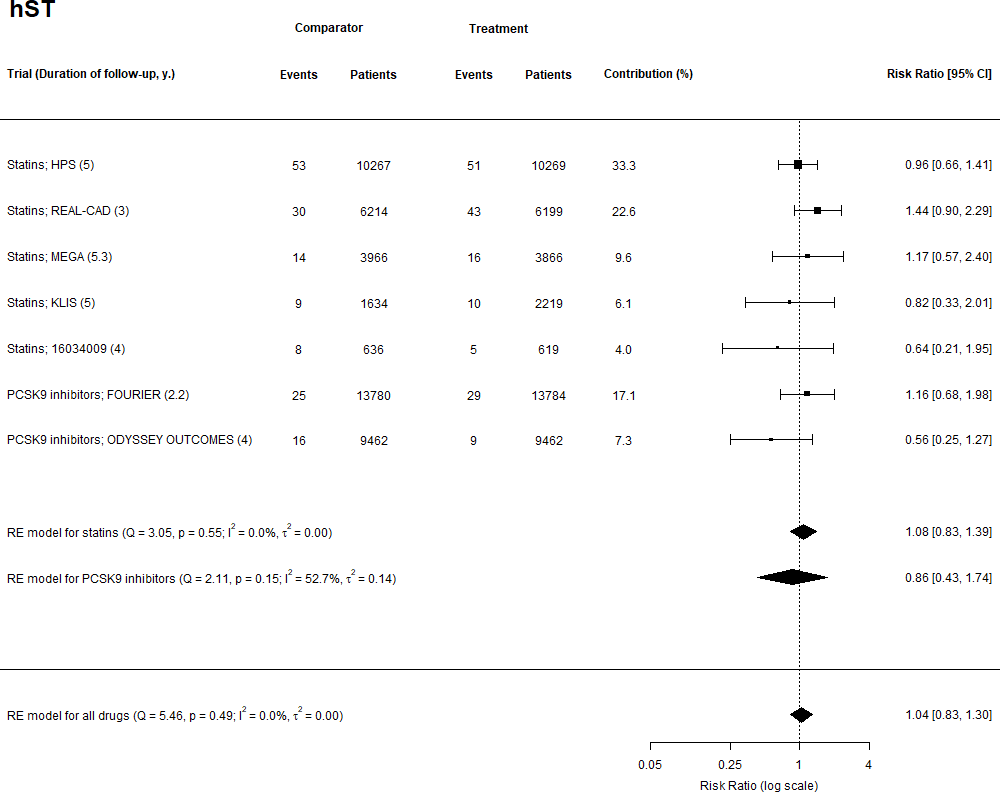

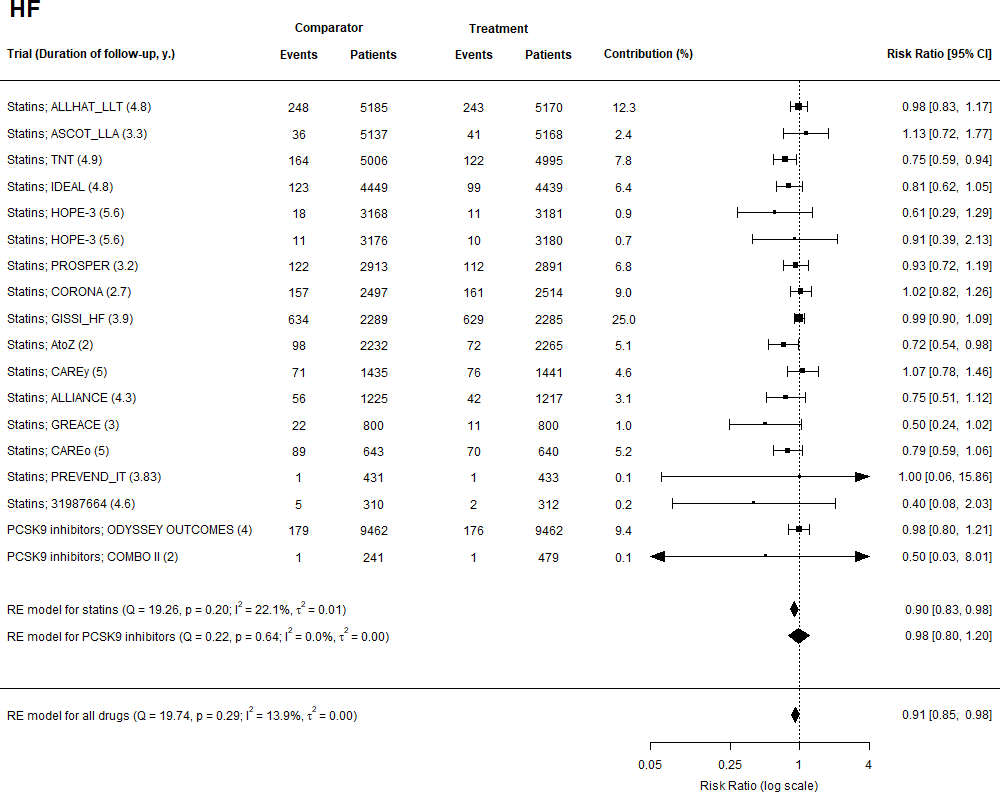

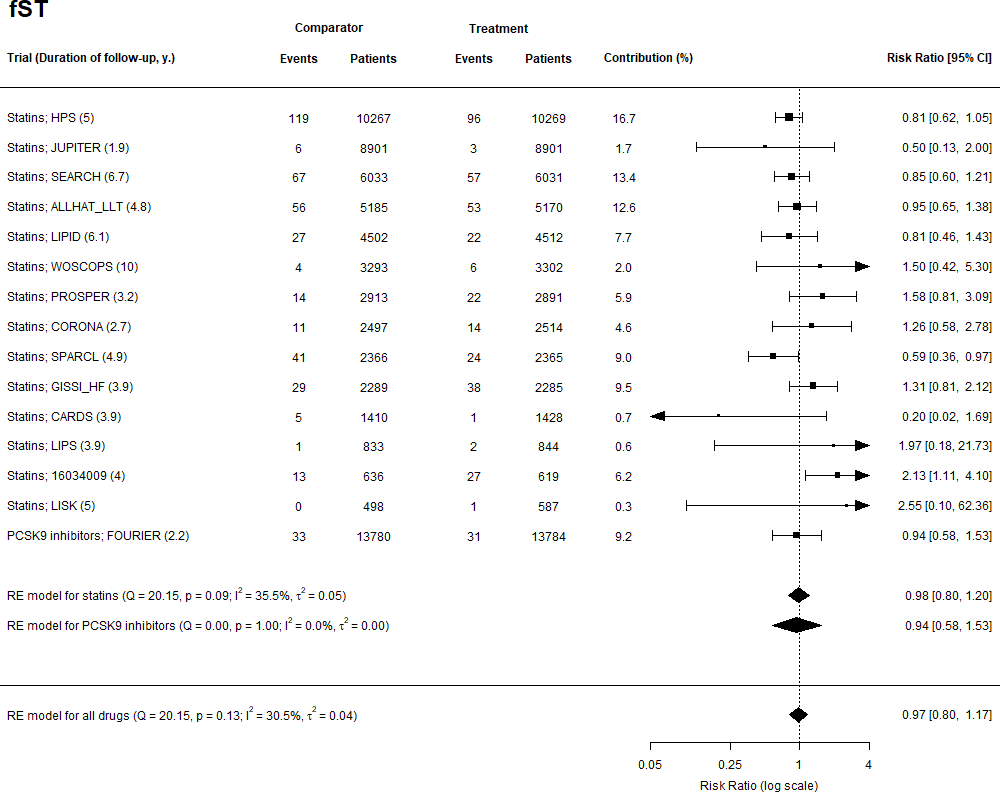

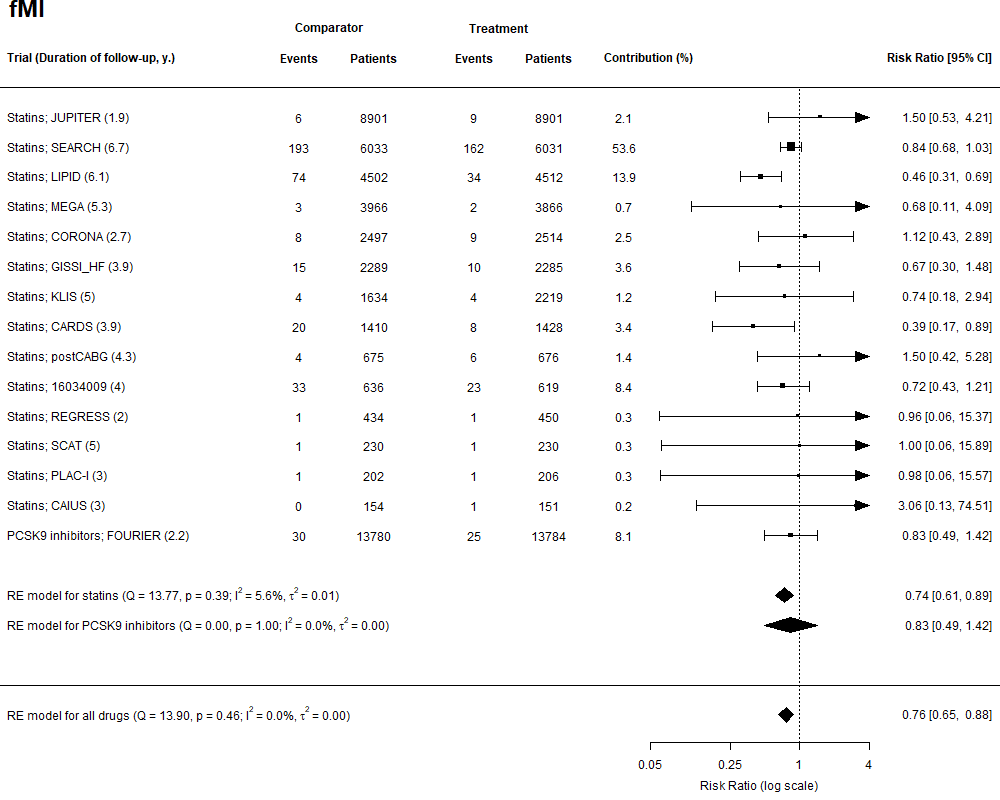

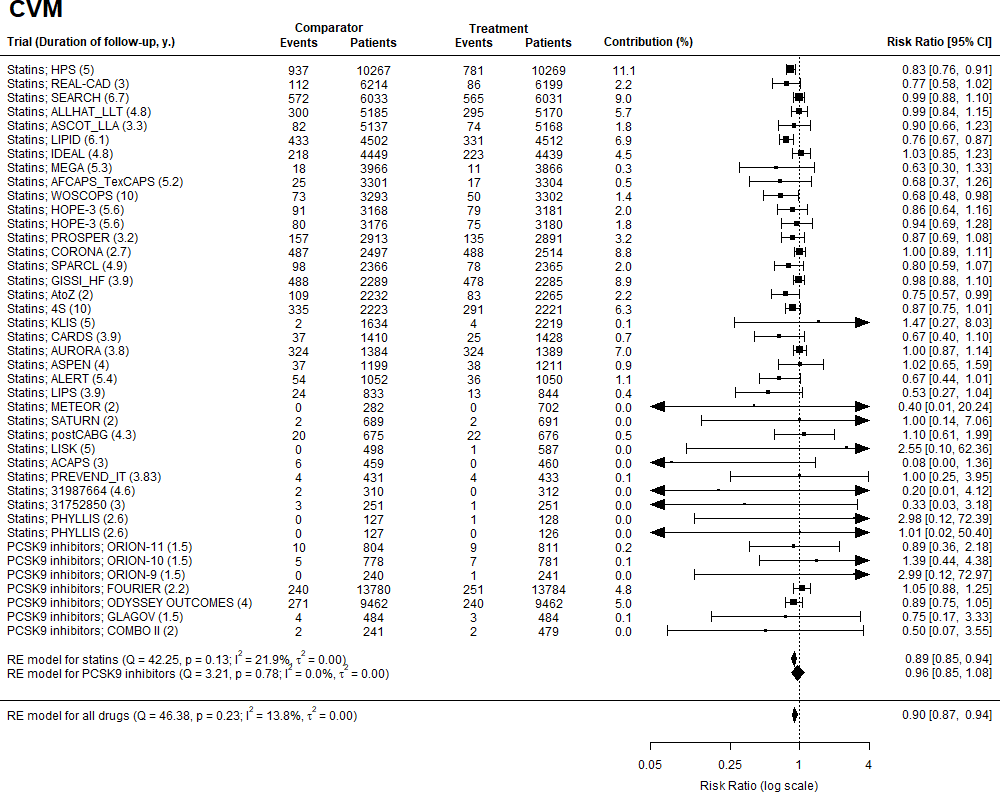

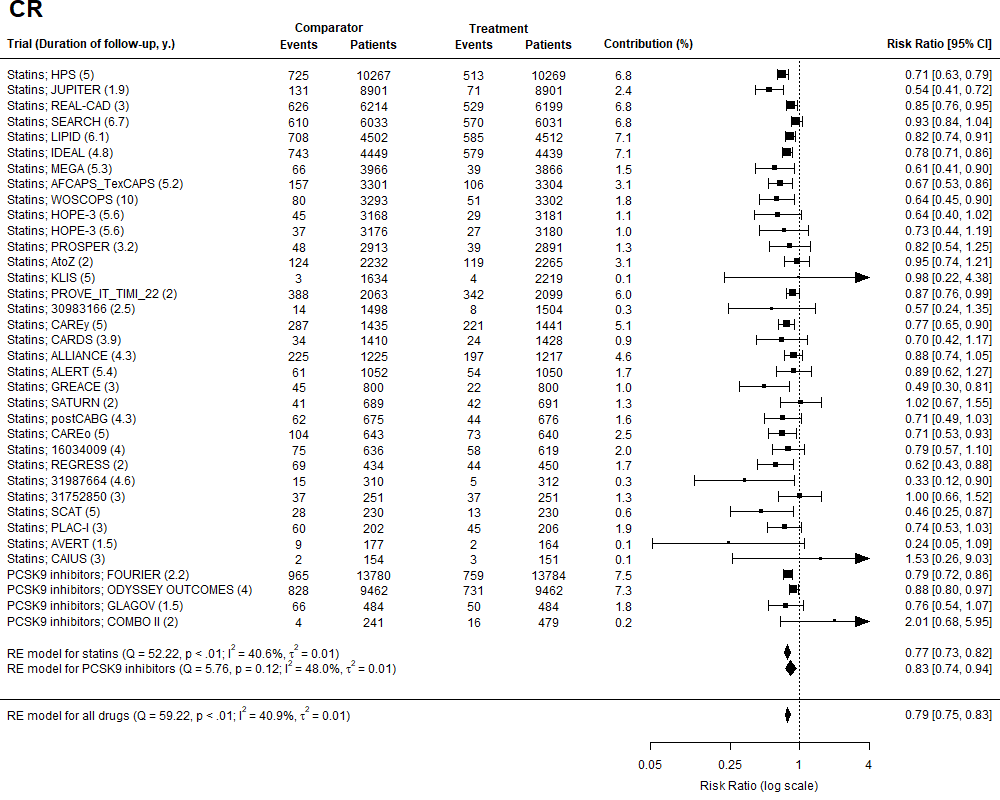

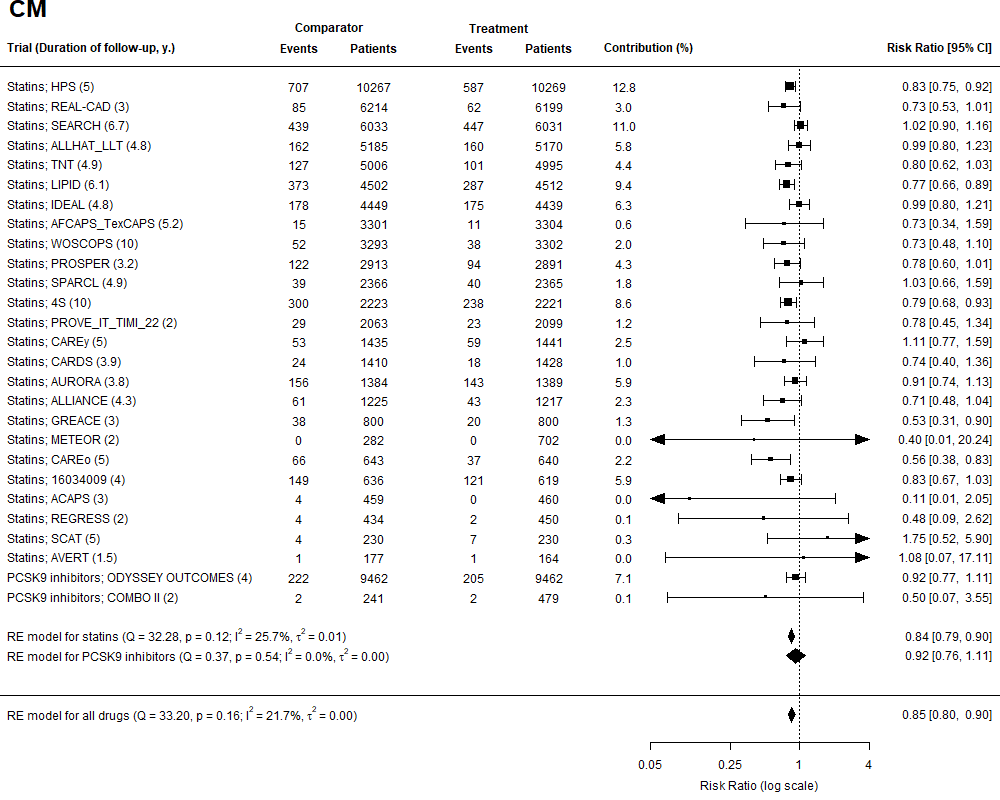

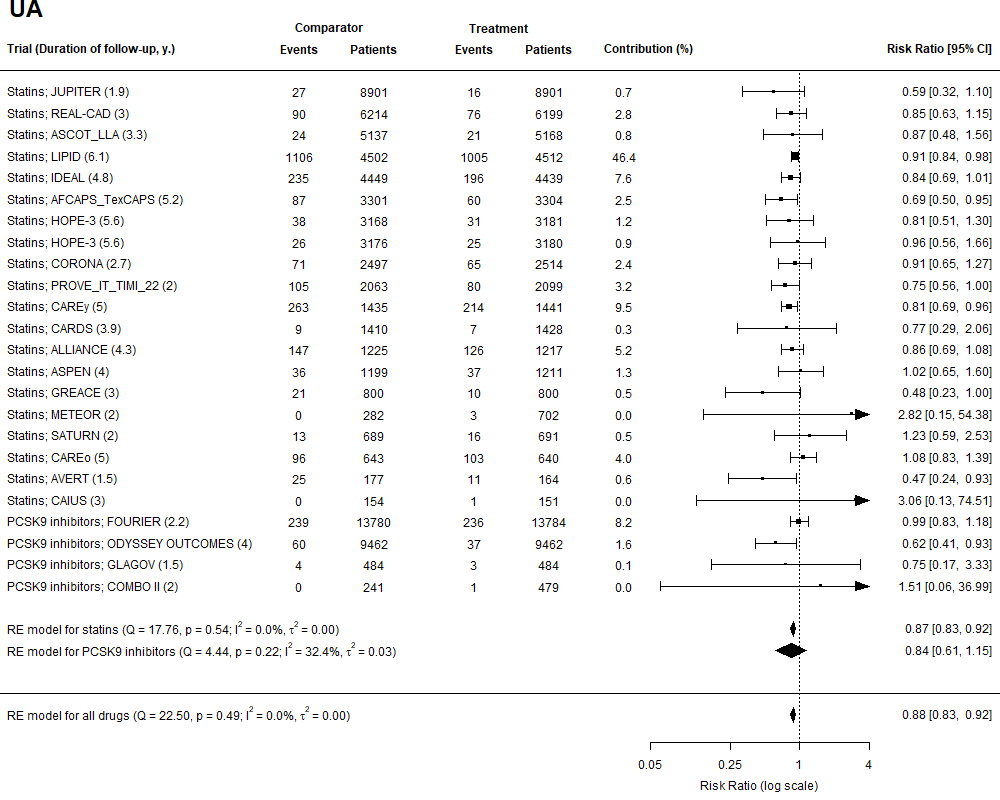


# Supplementary Figure 7. (A) Number of trials with statin and anti-PCSK9 therapies included or did not include the patients with renal disease per CV endpoint and (B) the average risk ratios (RR; mean with 95% confidence intervals) of MACE and individual MACE components before and after exclusion of studies with participants with severe renal impairment (RD)


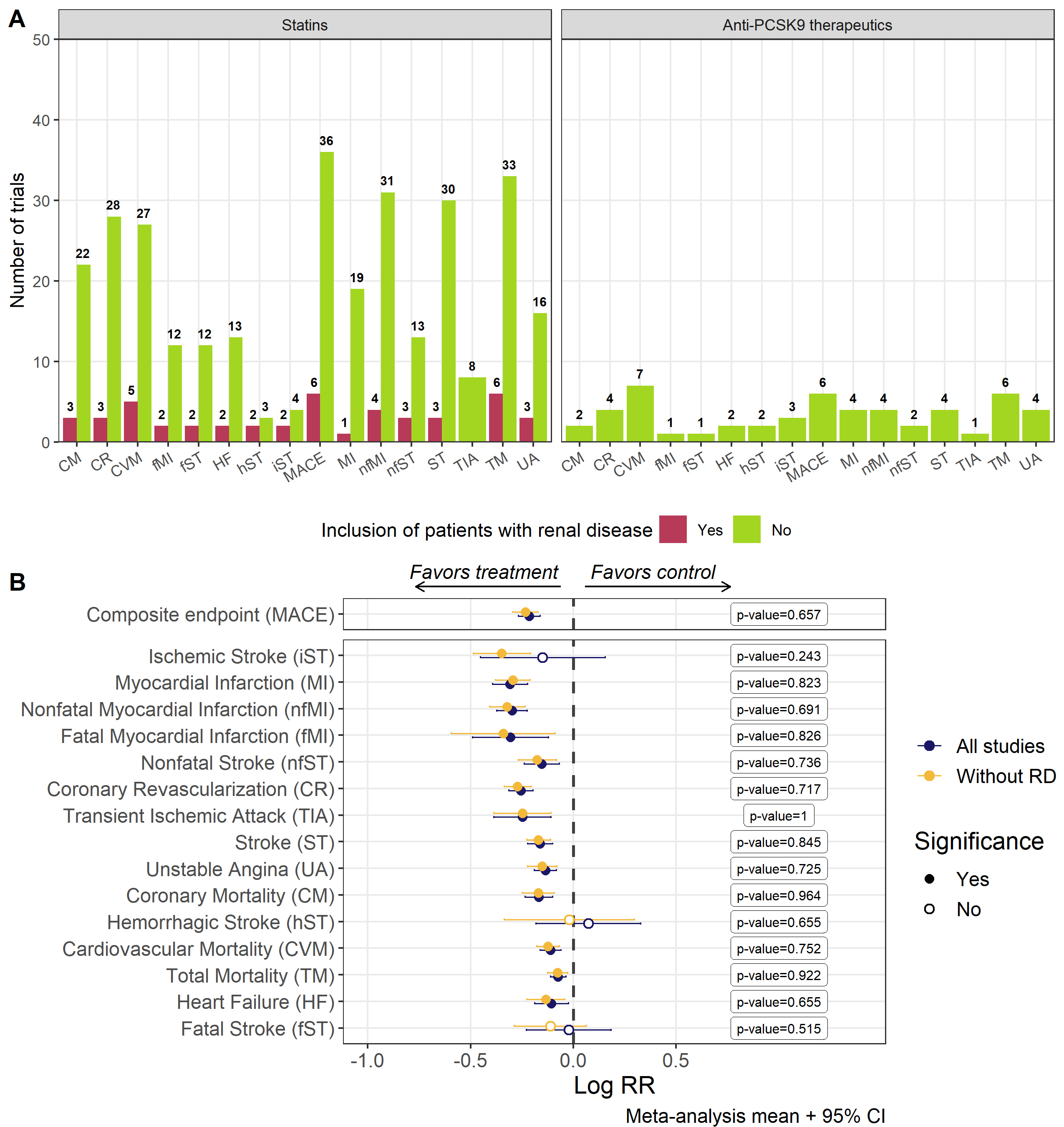


# Supplementary Figure 8. Weighted linear regression of the logarithm of RR and year of results publication, per CV event.

Red lines depict the weighted regression line with specified adjusted R2 (Adj R2) and p-value (P). Circles are sized according to the study weight.


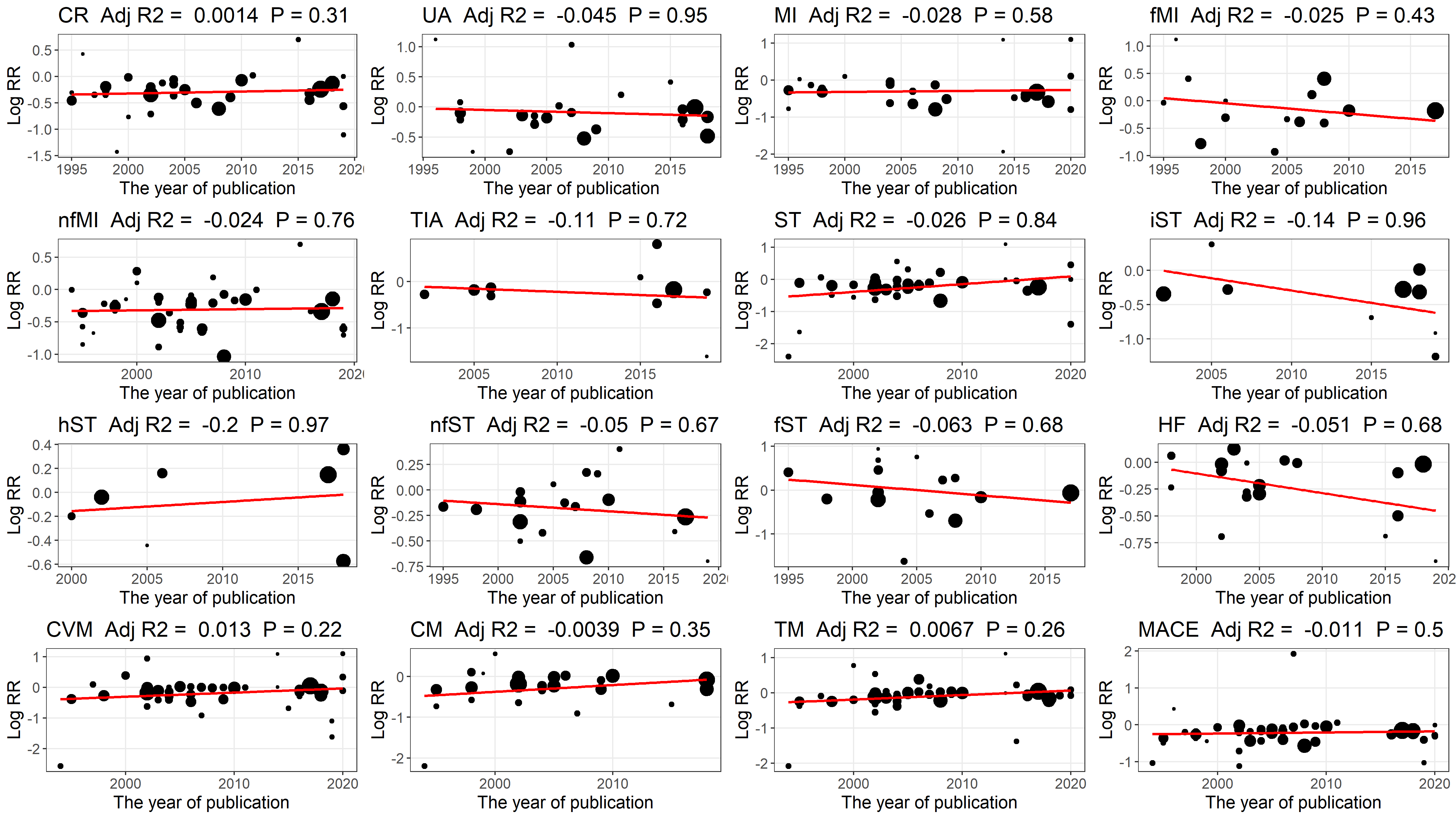

Supplement: Supplementary file 1 [file Table1.docx]
